# CACHE Challenge #3: Targeting the Nsp3 Macrodomain of SARS-CoV‑2

**DOI:** 10.1021/acs.jcim.5c02441

**Published:** 2026-01-21

**Authors:** Oleksandra Herasymenko, Madhushika Silva, Galen J. Correy, Abd Al-Aziz A. Abu-Saleh, Suzanne Ackloo, Cheryl Arrowsmith, Alan Ashworth, Fuqiang Ban, Hartmut Beck, Kevin P. Bishop, Hugo J. Bohórquez, Albina Bolotokova, Marko Breznik, Irene Chau, Yu Chen, Artem Cherkasov, Wim Dehaen, Dennis Della Corte, Katrin Denzinger, Niklas P. Doering, Kristina Edfeldt, Aled Edwards, Darren Fayne, Francesco Gentile, Elisa Gibson, Ozan Gokdemir, Anders Gunnarsson, Judith Günther, John J. Irwin, Jan Halborg Jensen, Rachel J. Harding, Alexander Hillisch, Laurent Hoffer, Anders Hogner, Ashley Hutchinson, Shubhangi Kandwal, Andrea Karlova, Kushal Koirala, Sergei Kotelnikov, Dima Kozakov, Juyong Lee, Soowon Lee, Uta Lessel, Sijie Liu, Xuefeng Liu, Peter Loppnau, Jens Meiler, Rocco Moretti, Yurii S. Moroz, Charuvaka Muvva, Tudor I. Oprea, Brooks Paige, Amit Pandit, Keunwan Park, Gennady Poda, Mykola V. Protopopov, Vera Pütter, Rahul Ravichandran, Didier Rognan, Edina Rosta, Yogesh Sabnis, Thomas Scott, Almagul Seitova, Purshotam Sharma, François Sindt, Minghu Song, Casper Steinmann, Rick Stevens, Valerij Talagayev, Valentyna V. Tararina, Olga Tarkhanova, Damon Tingey, John F. Trant, Dakota Treleaven, Alexander Tropsha, Patrick Walters, Jude Wells, Yvonne Westermaier, Gerhard Wolber, Lars Wortmann, Shuangjia Zheng, James S. Fraser, Matthieu Schapira

**Affiliations:** † Structural Genomics Consortium, 105607 University Health Network, Toronto, Ontario M5G 2C4, Canada; ‡ Department of Bioengineering and Therapeutic Sciences, University of California San Francisco, San Francisco, California 94158, United States; § Department of Chemistry and Biochemistry, University of Windsor, 401 Sunset Avenue, Windsor, Ontario N9B 3P4, Canada; ∥ Binary Star Research Services, LaSalle, Ontario N9J 3X8, Canada; ⊥ Department of Medical Biophysics, University of Toronto, Toronto, Ontario M5G 1L7, Canada; # Princess Margaret Cancer Centre, University Health Network, Toronto, Ontario M5G 2C4, Canada; ∇ Helen Diller Family Comprehensive Cancer Center, 8785 University of California, San Francisco, San Francisco, California 94143, United States; ○ Vancouver Prostate Centre, 2660 Oak Street, Vancouver, British Columbia V6H3Z6, Canada; ◆ Bayer AG, Drug Discovery Sciences, Wuppertal 42113, Germany; ¶ Drug Discovery Program, Ontario Institute for Cancer Research, Toronto, Ontario M5G 0A3, Canada; †† QuAccel, Toronto, Ontario M4M 1L8, Canada; ‡‡ Molecular Design Group, Institute of Pharmacy, Department of Biology, Chemistry & Pharmacy, Freie Universitaet Berlin, Koenigin-Luisestr. 2+4, Berlin 14195, Germany; §§ 8166 University of British Columbia, Vancouver, British Columbia V6H 3Z6, Canada; ∥∥ CZ-OPENSCREEN, Department of Informatics and Chemistry, Faculty of Chemical Technology, 52735 University of Chemistry and Technology Prague, Technická 5, 16 628 Prague 6, Czech Republic; ⊥⊥ Department of Organic Chemistry, Faculty of Chemical Technology, University of Chemistry and Technology Prague, Technická 5, 16 628 Prague 6, Czech Republic; ## Department of Physics and Astronomy, 6756 Brigham Young University, Provo 84602, Utah; ∇∇ Structural Genomics Consortium, Department of Medicine, Karolinska University Hospital and Karolinska Institutet, Stockholm 171 76, Sweden; ○○ DCU Life Sciences Institute, Dublin City University, Dublin D09 DXA0, Ireland; ◆◆ Molecular Design Group, School of Chemical Sciences, Dublin City University, Glasnevin, Dublin D09 V209, Ireland; ¶¶ Department of Chemistry and Biomolecular Sciences, 6363 University of Ottawa, Ottawa, Ontario K1N 6N5, Canada; ††† Ottawa Institute of Systems Biology, Ottawa, Ontario K1H 8M5, Canada; ‡‡‡ 2462 University of Chicago, Chicago, Illinois 60637, United States; §§§ Argonne National Laboratory, Lemont, Illinois 60439, United States; ∥∥∥ Protein, Structure and Biophysics, Discovery Sciences, BioPharmaceuticals R&D, AstraZeneca, Gothenburg 43150, Sweden; ⊥⊥⊥ Bayer AG, Drug Discovery Sciences, Berlin 13353, Germany; ### Department of Pharmaceutical Chemistry, University of California San Francisco, 1700 Fourth St, San Francisco, California 94158-2330, United States; ∇∇∇ Department of Chemistry, University of Copenhagen, 1353 Copenhagen, Denmark; ○○○ UCB BioSciences GmbH, Rolf-Schwarz-Schütte-Platz 1, 40789 Monheim am Rhein, Germany; ◆◆◆ Medicinal Chemistry, Research and Early Development, Cardiovascular, Renal and Metabolism (CVRM), BioPharmaceuticals R&D, AstraZeneca, Gothenburg 43150, Sweden; ¶¶¶ Molecular Design Group, School of Biochemistry and Immunology, Trinity Biomedical Sciences Institute, Trinity College Dublin, 152-160 Pearse St, Dublin 2 D02 R590, Ireland; †††† Trinity Biomedical Sciences Institute, School of Biochemistry and Immunology, Trinity College Dublin, 152-160 Pearse Street, Dublin 2 D02 R590, Ireland; ‡‡‡‡ Department of Computer Science, University College London, London WC1E 6BT, U.K.; §§§§ Eshelman School of Pharmacy, The University of North Carolina at Chapel Hill, Chapel Hill, North Carolina 27599, United States; ∥∥∥∥ Stony Brook University, Department of Applied Mathematics & Statistics, Stony Brook, New York 11794-3600, United States; ⊥⊥⊥⊥ Department of Molecular Medicine and Biopharmaceutical Sciences, Seoul National University, 08826 Seoul, South Korea; #### College of Pharmacy, Seoul National University, 08826 Seoul, South Korea; ∇∇∇∇ Arontier Co., 06784 Seoul, South Korea; ○○○○ Boehringer Ingelheim Pharma GmbH & Co., KG, Birkendorfer Str. 65, 88397 Biberach an der Riss, Germany; ◆◆◆◆ Institute for Drug Discovery, Faculty of Medicine, Faculty of Mathematics and Informatics, Faculty of Chemistry and Mineralogy, University Leipzig, Leipzig 04109, Germany; ¶¶¶¶ Center for Scalable Data Analytics and Artificial Intelligence ScaDS.AI Dresden/Leipzig and School of Embedded Composite Artificial Intelligence SECAI, Dresden/Leipzig 01062, Germany; ††††† Department of Chemistry, Center for Structural Biology, Vanderbilt University, South Nashville, Nashville, Tennessee 37240-0002, United States; ‡‡‡‡‡ Chemspace, Kyiv 02094, Ukraine; §§§§§ Taras Shevchenko National University of Kyiv, Kyiv 01033, Ukraine; ∥∥∥∥∥ Center for Natural Product Systems Biology, Korea Institute of Science and Technology, Gangneung 25451, Republic of Korea; ⊥⊥⊥⊥⊥ Experts System Inc.,12730 High Bluff Drive, Suite 100, San Diego, California 92130, United States; ##### AI Centre, Department of Computer Science, University College London, London WC1E 6BT, U.K.; ∇∇∇∇∇ School of Pharmacy and Technology Management, SVKM’s Narsee Monjee Institute of Management Studies (NMIMS) Deemed-to-be-University, Indore, Madhya Pradesh 453112, India; ○○○○○ Leslie Dan Faculty of Pharmacy, University of Toronto, Toronto, Ontario M5S 3M2, Canada; ◆◆◆◆◆ Nuvisan ICB GmbH, Berlin 13353, Germany; ¶¶¶¶¶ Laboratoire d’Innovation Thérapeutique, UMR7200 CNRS-Université de Strasbourg, 67400 Illkirch, France; †††††† Department of Physics and Astronomy, University College London, London WC1E 6BT, U.K.; ‡‡‡‡‡‡ UCB Pharma, Braine-L’Alleud 1420, Belgium; §§§§§§ Institute of Health and Medicine, Hefei Comprehensive National Science Center, Hefei, Anhui 230000, China; ∥∥∥∥∥∥ Department of Chemistry and Bioscience, Aalborg University, Fredrik Bajers Vej 7H, Aalborg, DK-9220, Denmark; ⊥⊥⊥⊥⊥⊥ WE-SPARK Health Institute, 401 Sunset Avenue, Windsor, Ontario N9B 3P4, Canada; ###### Conscience Medicines Network, Toronto, Ontario M5G 1L7, Canada; ∇∇∇∇∇∇ Relay Therapeutics, Cambridge, Massachusetts 02141, United States; ○○○○○○ Department of Computer Science, University College London, London WC1E 6BT, U.K.; ◆◆◆◆◆◆ Boehringer Ingelheim RCV, Dr. Boehringer-Gasse 5-11, 1121 Vienna, Austria; ¶¶¶¶¶¶ Boehringer Ingelheim Pharma GmbH & Co., KG, Birkendorfer Str. 65, Biberach an der Riss 88397, Germany; ††††††† Shanghai Jiao Tong University, Shanghai 200030, China; ‡‡‡‡‡‡‡ Department of Bioengineering and Therapeutic Sciences, University of California San Francisco, San Francisco, California 94158, United States; §§§§§§§ Princess Margaret Cancer Centre, University Health Network, Toronto, Ontario M5G 2C4, Canada; ∥∥∥∥∥∥∥ Department of Pharmacology & Toxicology, University of Toronto, Toronto, Ontario M5S 1A8, Canada; a Department of Chemistry, Department of Pharmacology, Center for Structural Biology, Institute of Chemical Biology, Center for Applied Artificial Intelligence in Protein Dynamics, Vanderbilt University, Nashville, Tennessee 37240-0002, United States

## Abstract

The third *Critical Assessment of Computational Hit-finding Experiments* (CACHE) challenged computational teams to identify chemically novel ligands targeting the macrodomain 1 of SARS-CoV-2 Nsp3, a promising coronavirus drug target. Twenty-three groups deployed diverse design strategies to collectively select 1739 ligand candidates. While over 85% of the designed molecules were chemically novel, the best experimentally confirmed hits were structurally similar to previously published compounds. Confirming a trend observed in CACHE #1 and #2, two of the best-performing workflows used compounds selected by physics-based computational screening methods to train machine learning models able to rapidly screen large chemical libraries, while four others used exclusively physics-based approaches. Three pharmacophore searches and one fragment growing strategy were also part of the seven winning workflows. While active molecules discovered by CACHE #3 participants largely mimicked the adenine ring of the endogenous substrate, ADP-ribose, preserving the canonical chemotype commonly observed in previously reported Nsp3-Mac1 ligands, they still provide novel structure–activity relationship insights that may inform the development of future antivirals. Collectively, these results show that multiple molecular design strategies can efficiently converge on similar potent molecules.

## Graphical abstract

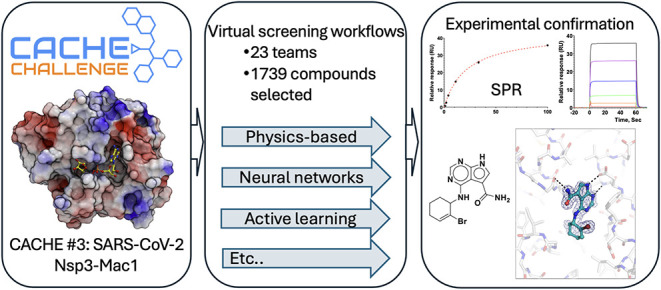

## Introduction

The critical assessment of computational hit-finding experiments (CACHE) is a series of benchmarking challenges to objectively delineate the state-of-the-art in computational hit-finding,^1,2^ assess the impact of machine learning (ML) in the field, and inform future developments through direct head-to-head time-restricted competition. A novel protein target is nominated every few months and applications from computational chemists and ML experts are then evaluated via a double-blinded peer-review process, where each applicant blindly scores the computational methods proposed by five other applicants. The resulting ranking is reviewed and approved by an independent Applications Review Committee (Table S1) with minor adjustments, and the top 25 applicants are invited to submit, within 3 months, a list of 100 drug-like ligand candidates from Enamine REAL. Physical compounds are procured and tested experimentally. Participants with at least one compound of interest (i.e., with a binding signal observed biophysically) are invited to select up to 50 close commercially available synthetically accessible analogs of their experimental hits that are again procured and tested. The goal is to confirm binding of multiple compounds sharing a given molecular scaffold and thus establish high-confidence chemical series against the target protein. This effort collectively seeks to demonstrate the true current capabilities of computational drug discovery in a blinded fashion. The first two CACHE challenges reflected a great diversity in computational workflows and revealed a few recurrently successful strategies, including fragment-based design approaches and ultrafast screening with ML models trained on docking scores.^3,4^

The third CACHE challenge was focused on macrodomain 1 of SARS-CoV-2 Nsp3 (Mac1, Figure 1a). Mac1 is an emerging antiviral target due to the antagonistic role of its enzymatic ADP-ribosylhydrolase activity in interferon response.^5^ Mutational studies, first in SARS-CoV-1 ^6^ and then in SARS-CoV-2,^5,7^ have validated Mac1 as a drug target. Starting from a fragment screen that identified more than 200 binders,^8^ distinct efforts from the ASAP^9^ and QCRG^10,11^ Antiviral Drug Discovery (AViDD) Centers have identified molecules with nanomolar potency. Importantly, the QCRG AVIDD molecule has shown *in vivo* protection in a lethal animal model of SARS CoV 2 infection.^10^ At the outset of CACHE #3, the original fragments^8^ and an initial series of ligands^12^ were in the public domain, most of which either bound with moderate potency (*K_i_* > 30 μM) or featured a carboxylic moiety that was deemed a chemical liability in cellular assays^8,12^ (Figure 1b). Importantly, these active compounds shared a chemical scaffold mimicking the adenine bicyclic ring of ADP ribose (ADPr), the endogenous substrate (Figure 1b). CACHE #3 participants were asked to discover compounds binding Nsp3-Mac1 satisfying two properties: (1) a novel chemical scaffold and (2) no carboxylic acid.

**Figure 1:**
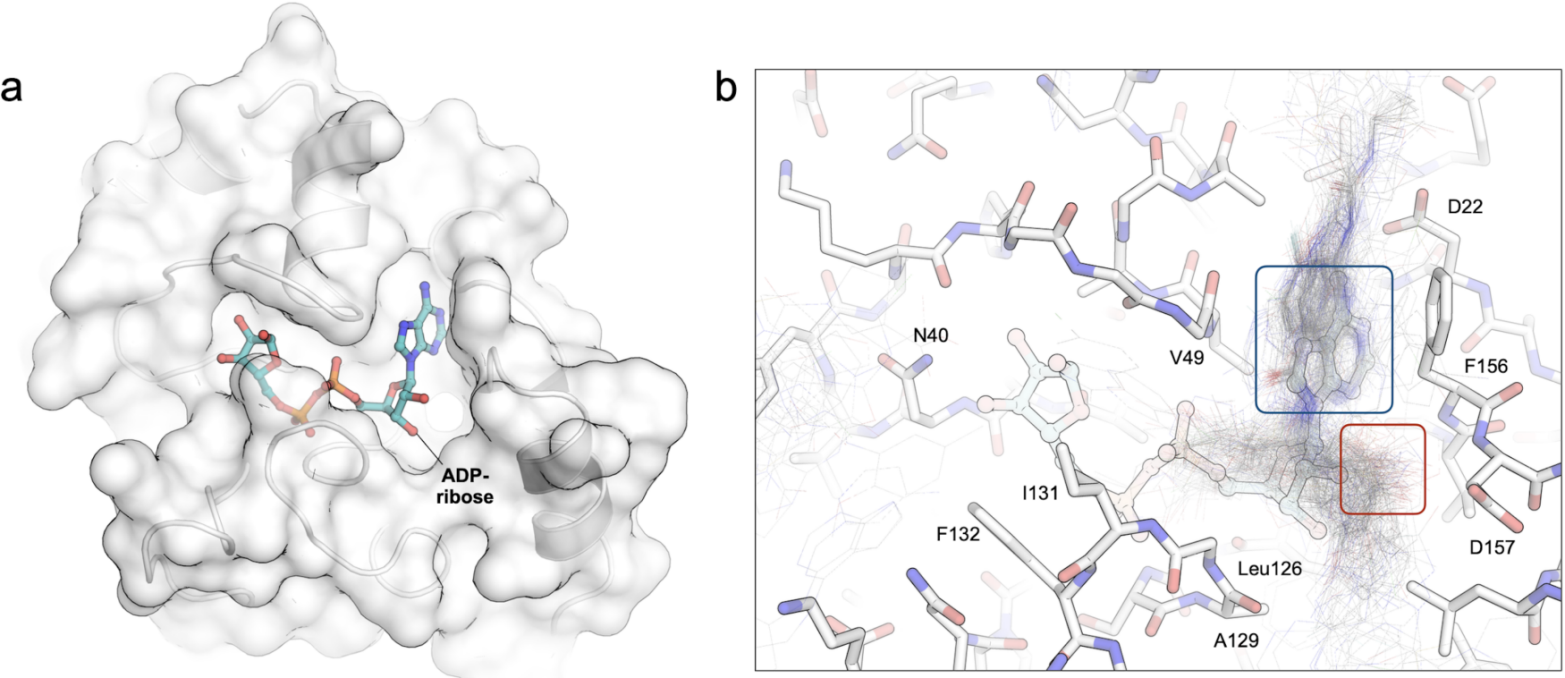
Active site of Macro domain 1 of SARS-CoV-2 Nsp3 (Mac1). (A) The domain of Mac1 showing the ADP-ribose (hydrolysis product) binding cavity from the PDB structure 7KQP. (B) A zoom into the adenosine portion of the active site with molecules (fragments and hit molecules) that had been published prior to the CACHE challenge^8,12,13^ shows the prevalence of adenine (blue box) and carboxylic acids (red box) in existing compounds.

The vast majority of compounds submitted by the competitors were chemically novel, but the molecules that were experimentally confirmed to bind tended to be structurally similar to the previously published inhibitors, or contained unwanted carboxylic acids observed in previous structures (Figure 1). This suggests that either the Enamine REAL database lacks alternative templates that are able to efficiently engage the targeted site, or that *in silico* workflows, regardless of the approach, cannot identify the ones that are present. Two of the seven best performing methods included a deep learning step, one adopted a random forest classifier as a preliminary filter, while the last four were purely physics-based. As in the first two CACHE challenges, CACHE #3 again suggests that there is no single best method for rapid computational hit-finding.

## Results

### Computational Workflows Were Diverse

Twenty-three of the 25 CACHE #3 participants submitted their predictions within the required two-month time frame. The participants employed computational workflows that greatly varied in their selection strategy, tools, and techniques (Figure 2). Seventeen groups implemented a ML step in their selection process, eight used molecular dynamics to account for protein flexibility, five applied binding free energy prediction methods to refine their selection, four used information from multiple bound molecules in the PDB to guide pharmacophore-based searches, and two included quantum mechanics calculations to increase precision. All participants used docking at some point in their workflow.

**Figure 2:**
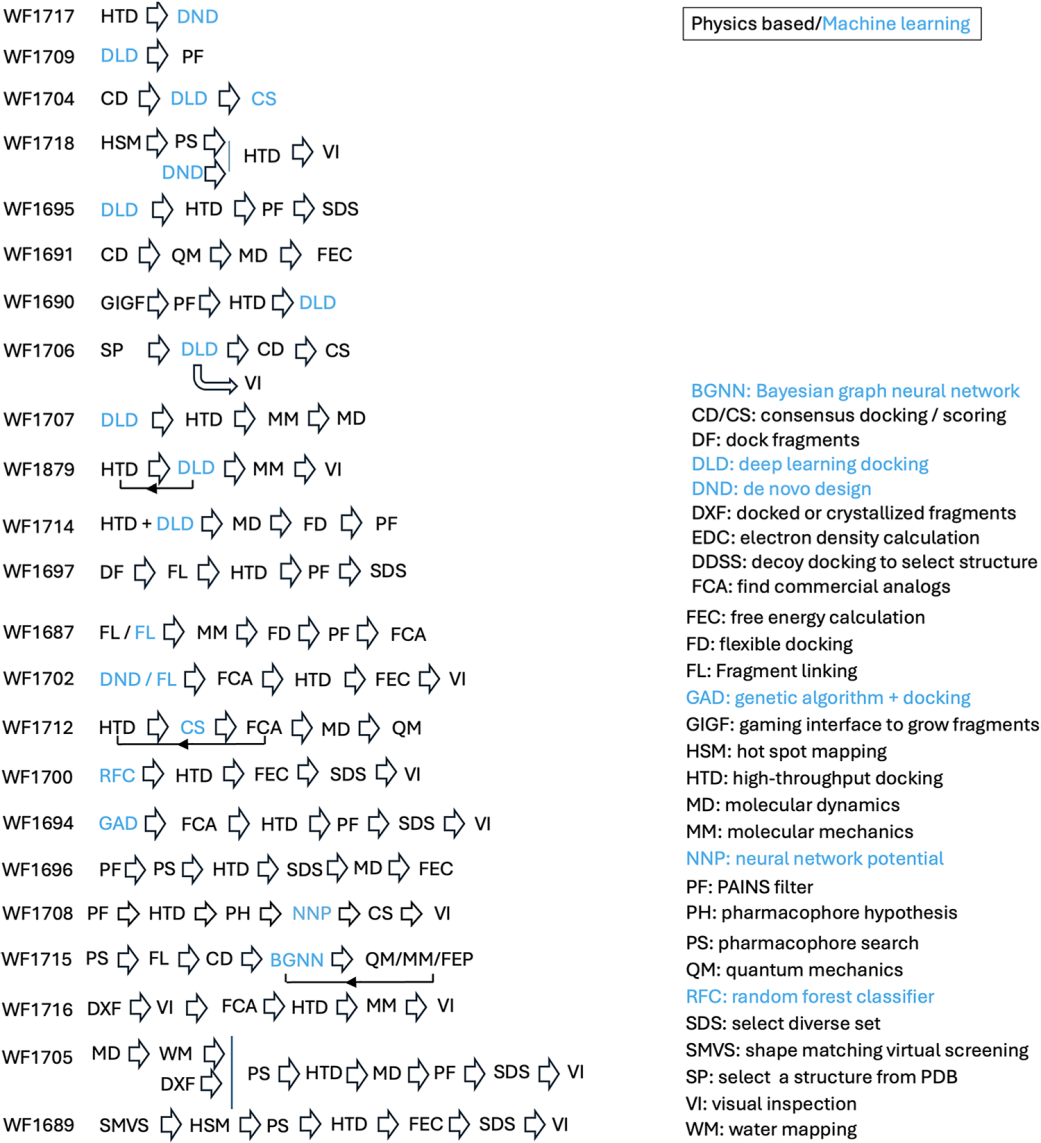
CACHE #3 computational workflows. The workflows integrate various ML and physics-based computational techniques in varying orders and combinations, strongly implying that the field has not yet identified a universal recipe for success.

For example, WF1706 used a Deep Docking algorithm,^14^ where a small subset of a larger library is docked with Glide (Schrödinger, Inc.) to generate a training set for a deep neural network that can then rapidly predict scores for billions of compounds. The top predicted molecules are further assessed via consensus docking with Glide and ICM (Molsoft L.L.C.). WF1705 adopted an entirely different and purely physics-based approach, where PyRod/LigandScout (Inte:Ligand GmbH) pharmacophore models were applied to filter docking poses from GOLD (The Cambridge Crystallographic Data Center, CCDC) followed by structure refinement and visual inspection with LigandScout. These diverse methods both placed in the top four in CACHE #3, and Deep Docking was also one of the best performing methods in CACHE #1.^3^

### Compounds Were Drug-like and Chemically Diverse

In the hit identification round (Round 1), participants were each able to select up to 100 commercially available compounds. Collectively, CACHE #3 participants selected 1739 compounds that were successfully synthesized by Enamine and shipped for experimental testing (Table S2). The compounds were reasonably well distributed across participants, with selections ranging from 41 to 99 molecules (Figure 3a). Overall, compounds were drug-like and satisfied Lipinski’s rule of five (Figure 3b). Participants were also asked to verify that their selected molecules passed the chemical liability filter badapple,^15^ though this was not mandatory.

**Figure 3:**
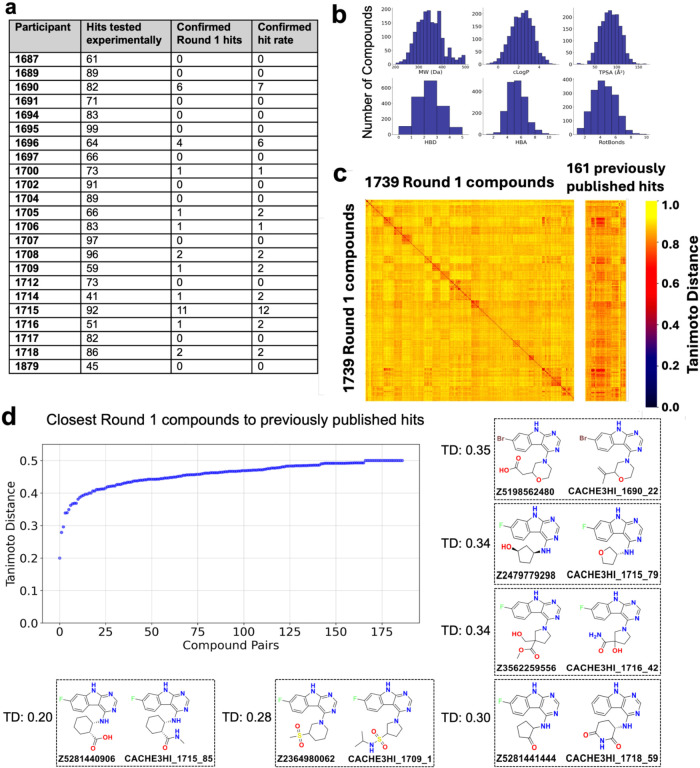
Drug-likeness, diversity, and novelty of CACHE #3 Round 1 compounds. (a) Distribution of tested and experimentally confirmed compounds across participants. (b) Distribution of physicochemical descriptors calculated with RDKit. TPSA: total polar surface area; HBA/HBD: H-bond acceptors/donors; Rot Bonds: number of rotatable bonds. (c) Tanimoto distance matrix of Round 1 compounds versus themselves (left) and versus 161 previously published Nsp3-Mac1 ligands.^12^ (d) Distance distribution and structures of the closest analogs to published molecules. TD: Tanimoto distance calculated with RDKit ECFP4 fingerprints.

Round 1 compounds were chemically diverse, as reflected by a Tanimoto distance matrix determined using RDKit 2048-bit ECFP4 fingerprints (Figure 3c). 1298 compounds were separated from molecules selected by other participants by a Tanimoto distance greater than 0.6. The diversity within each participant’s selection is highlighted by darker squares along the diagonal of the distance matrix. Importantly, predicted molecules were overall chemically distinct from the 161 Nsp3-Mac1 ligands previously published,^12^ reflected by Tanimoto distances greater than 0.6 for 1592 Round 1 compounds (Figure 3C). This is emphasized by the fact that only six Round 1 compounds had a Tanimoto distance less than 0.35 (Figure 3D).

### Discovering Chemically Novel Ligands Was Challenging

All 1739 compounds were first tested at 100 μM in a homogeneous time-resolved fluorescence (HTRF) biophysical assay, measuring the displacement of an ADPr peptide^12^ (Figure 4). A total of 302 compounds reduced the binding signal by 30% or more in at least one of two duplicates, or produced ambiguous data, for instance due to interference with the fluorescence signal, and were tested in a limited 3-points dose response at 25, 50, and, 100 μM, leading to 166 molecules tested in an orthogonal surface plasmon resonance (SPR) binding assay (see Methods section for details). In the end, 28 compounds selected by nine participants were confirmed in a 6-point SPR-dose response assay (Figure 4 and Tables S2 and S3) and advanced to the hit expansion phase (Round 2).

**Figure 4:**
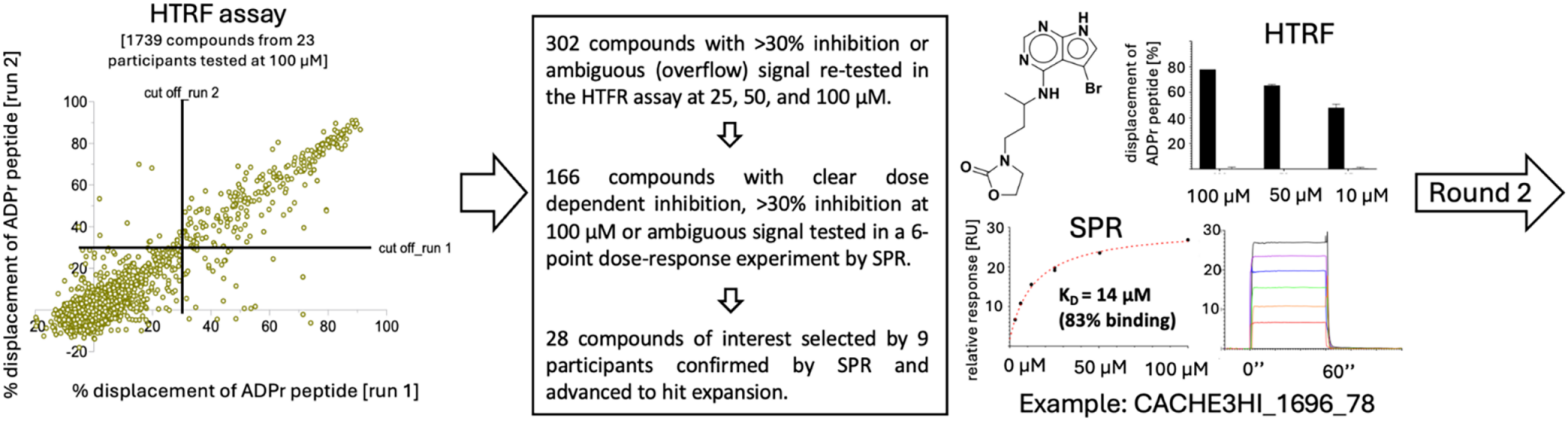
Round 1 experimental pipeline. Primary screening data by homogeneous time-resolved fluorescence (HTRF, left), pipeline progression statistics (middle), and data for one of the hits (right) are shown.

In parallel, all 1739 compounds were used in independent soaking experiments with crystallized Nsp3-Mac1, leading to complex structures for 44 of them, all binding at the ADPr site (Table S4). Twenty-two crystallized compounds previously showed dose-dependent binding by SPR. The rest did not show significant binding by SPR or did not show a dose-dependent inhibition in the preliminary 3-point dose response HTRF assay, possibly because their binding affinity was insufficient to be detected. Nevertheless, all 44 compounds were retested in an SPR dose–response experiment, and three were rescued as hits, but not advanced to Round 2 due to time constraints.

At the outset of CACHE #3, an explicit goal for participants was to find chemically novel ligands with high affinity that did not have a carboxylic acid. The 10 previously published ligands with a *K_i_* < 100 μM and no carboxylic acid all featured a bi- or tricyclic ring system shown crystallographically to recapitulate critical interactions engaged by the adenine ring of the endogenous substrate, ADPr (Figure 1b).^8,12^ Despite this being an explicit condition, 15% of the CACHE #3 Round 1 compounds still featured carboxylates. Strikingly, 27 of the 28 Round 1 compounds that advanced to Round 2 featured the canonical adenine mimic, clearly demonstrating that finding chemically novel ligands was challenging. The exception was CACHE3HI_1700_52, which had a *K*_D_ of 28 μM (Figure 5 and Table 1).

**Figure 5:**
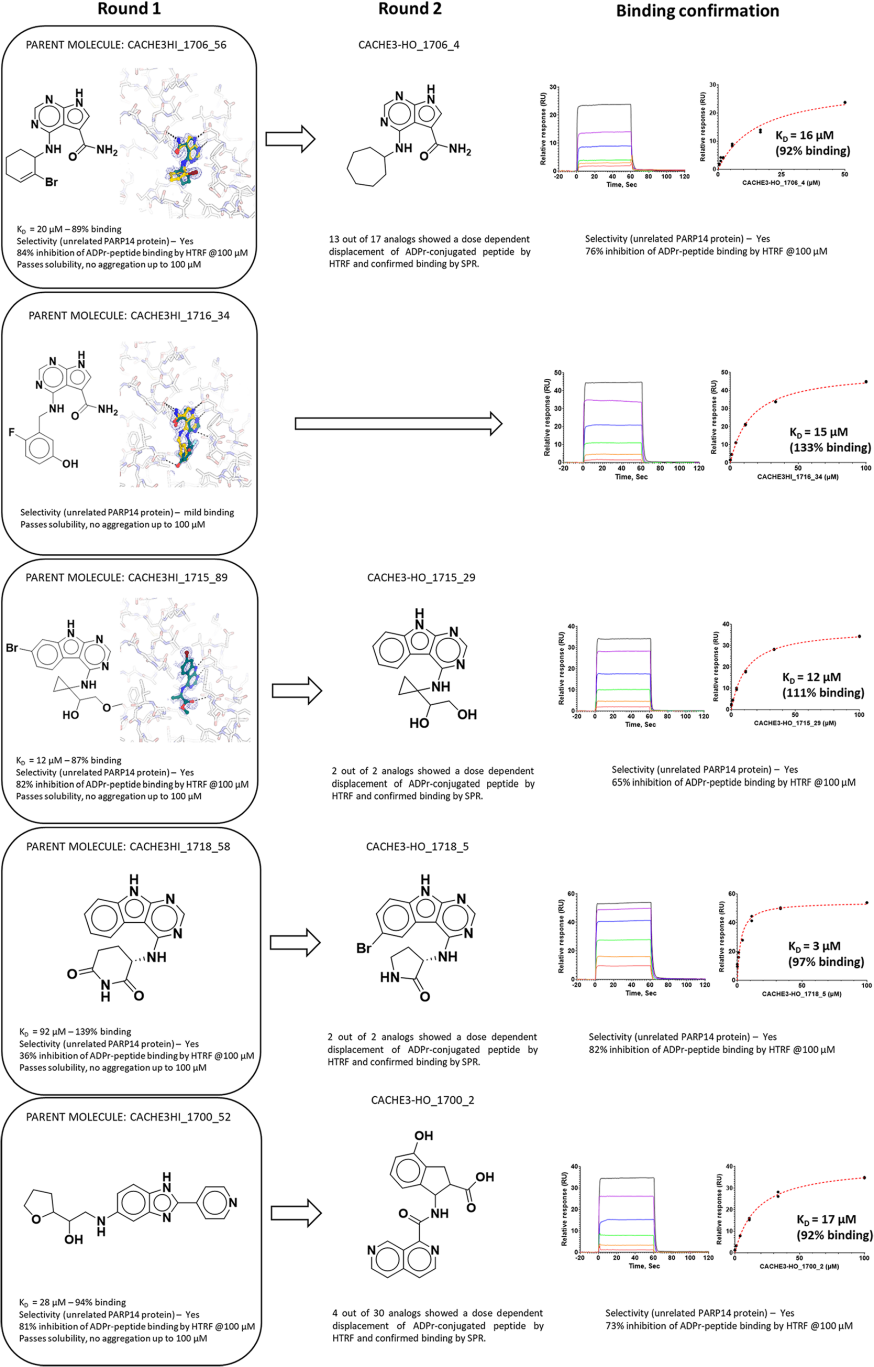
Sample CACHE #3 chemical series. Chemical structures, activity data, and crystal structures, where available, are shown for parent (Round 1) molecules (left) and active analogs (Round 2, right). CACHE3HI_1700_52 (bottom) was the most chemically novel molecule but could not be confirmed in Round 2 as selected follow-up compounds were not analogs. CACHE3HI-1716_34 was rescued by crystallography and no analogs were selected in Round 2. Similar data on all CACHE #3 chemical series are available in the Supporting Information.

**Table 1:**
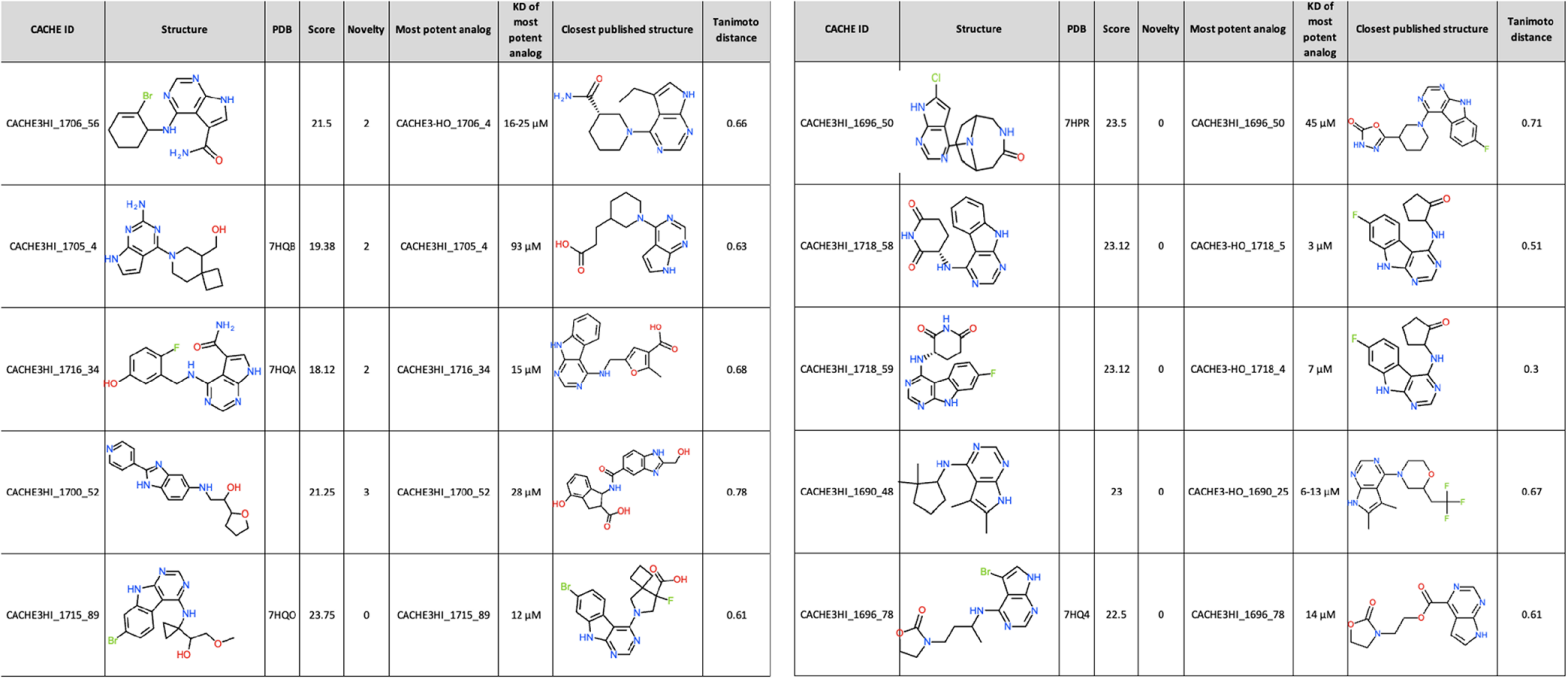
Best Scoring CACHE #3 Compounds^a^

a For each of the 10 top-scoring Round 1 compounds, their CACHE IDs, structure, activity & novelty scores are listed alongside the *K*_D_ of the most potent Round 1 or Round 2 molecule and the structure and Tanimoto distance from the closest previously published analog.

### Selection and Experimental Testing of Round 2 Compounds

In the hit expansion phase of the CACHE challenges (Round 2), computational teams can select up to 50 close commercial analogs of their compounds experimentally confirmed in Round 1 (compounds of interest) to verify that the binding signal is not limited to a single batch of a single molecule, and therefore at risk of being spurious. A total of 296 analogs of the 28 compounds from nine computational teams advanced to Round 2 were successfully synthesized and tested experimentally. As in Round 1, the experimental pipeline was HTRF (Table S5) followed by SPR (Table S6).

Overall, 114 compounds, including 17 resupplied parent molecules, showed clear dose-dependent inhibition in the HTRF assay and were advanced to the next step. Another 44 compounds that showed >30% inhibition at 100 μM with no dose-dependent inhibition, or >17% inhibition at 100 μM with a dose-dependent inhibition were also advanced. The maximum compound concentration that could be used in the following SPR dose response experiment was based on compound solubility evaluated by dynamic light scattering (DLS). The resulting 158 compounds, in addition to 16 Round 1 molecules that produced artifact signals in HTRF but were rescued by X-ray crystallography, were tested in a 6-points dose–response by SPR, leading to 84 confirmed hits. All hits were then retested by SPR against Nsp3 and the macro 1 domain of the antitarget PARP14 (selected because it also binds ADPr) to verify that binding was specific (Table S6). This experimental workflow delivered a data package for each chemical series (Supporting Information and Figure 5) that was evaluated by an independent hit evaluation committee composed of biophysicists as well as medicinal and computational chemists from the pharmaceutical industry (Table S1).

### Evaluation of Experimental Data and Computational Workflows

Each of the 28 Round 1 hits as well as the three Round 1 compounds rescued by crystallography that had a measurable *K*_D_ were assigned a score reflecting the quality and consistency of the binding signals and their chemical soundness, including absence of undesirable groups (Table S7). The three chemists from the Hit Evaluation Committee also independently evaluated the chemical novelty of the molecules, resulting in a novelty score between 0 (not novel) and 3 (unanimously voted as novel) (Tables S7 and 1).

Eight of the 10 top scoring chemical series (activity score >15) included molecules with *K*_D_ values ranging from 3 to 28 μM, demonstrating that CACHE #3 participants could identify reasonably potent hits for the target that were devoid of carboxylates (Table 1). We note that five of the previously published carboxylate-free ligands had *K_i_* values in a similar range.^12^ CACHE #3 hits were overall not chemically novel. Only four had a novelty score >0, including three featuring the previously published adenine ring mimic decorated with a nonobvious amide group (CACHE3HI_1706_56 and CACHE3HI_1716_34) or linked to a spirocyclic 7-(azaspiro[3.5]­nonan-5-yl)­methanol (CACHE3HI_1705_4). Some compounds from Round 2 also converged toward this nonobvious amide group (ex: CACHE3-HO_1708_5). No active analogs of the only significantly novel compound (CACHE3HI_1700_52; novelty score = 3) were found in Round 2 (Figure 5) and attempts to solve the crystal structure of the complex were unsuccessful. This suggests that finding novel chemical scaffolds targeting the ADPr binding pocket of Nsp3-Mac1 is a challenging endeavor. Indeed, to the best of our knowledge, all ligands with clear experimental confirmation publicly reported to date are based on templates originally published by Schuller et al.^8^ (Figure 1). Exploring chemical libraries from other sources may reveal novel favorable scaffolds.

Overall, nine of the 23 workflows delivered at least one high-scoring Round 1 compound (score >15), though molecules were chemically novel for only four of them (Figure 6a). Workflow WF1715 not only produced the top scoring compound (CACHE3HI_1715_89) but also identified significantly more hits than others, as reflected by the aggregated score of Round 1 molecules (Figure 6b), though none of the compounds were deemed novel by the Hit Evaluation Committee. Computational teams were also asked to predict active molecules from the merged list of all Round 1 compounds, which is a complementary evaluation scheme, as here, all computational workflows are applied onto the same chemical library. To evaluate predictions, experimentally confirmed hits with a score greater than 15 were clustered into two tiers: four “Tier 1” compounds that were chemically novel and 25 “Tier 2” compounds judged not chemical novel. Workflow WF1696, a purely physics-based method, retrieved significantly more hits than any of the other workflows, including both novel and non-novel examples (Figure 6c).

**Figure 6:**
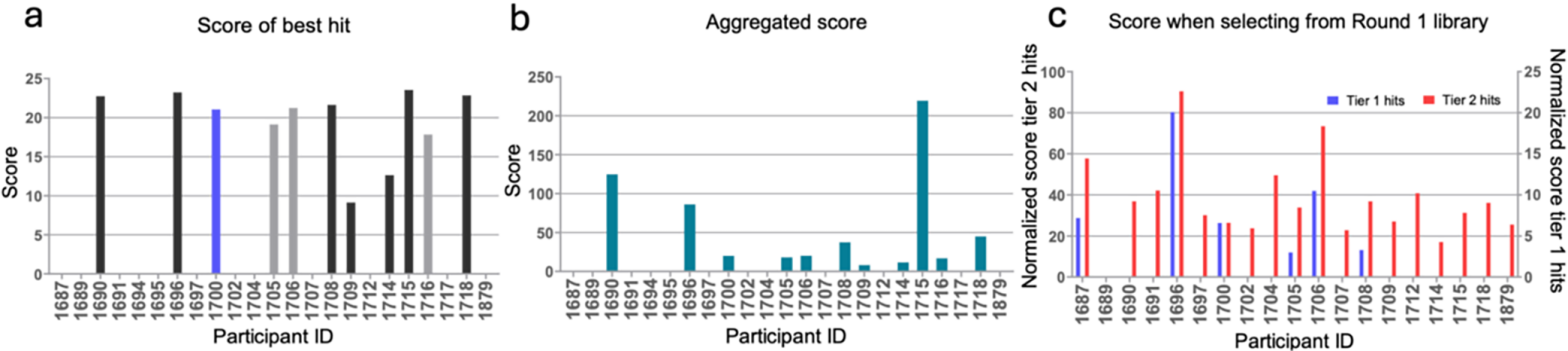
Scores of CACHE #3 participants. For each participating team, the score of the best Round 1 hit (a) and the aggregated score of all Round 1 hits (b) are plotted. (c) The normalized number of experimentally confirmed Tier 1 hits (chemically novel) and Tier 2 hits (not novel) predicted active compounds when screening all 1739 Round 1 compounds (calculated as the aggregated score of all compounds predicted active divided by the number of compounds predicted to be actives). Color coding blue, gray, and black in (a) indicates chemical novelty scores of 3, 2, and 0, respectively. Details are provided in Table S8.

### Trends and Strategies from the Best Performing Computational Workflows

The computational workflows producing top scoring compounds, top hit rates, and top selections from the merged Round 1 library (Figure 6, panels a, b, and c, respectively) represented diverse screening pipelines and design strategies (Figure 7), but some trends and commonalities also emerged (Figure 8). While only seven workflows are highlighted in Figure 8, this does not imply that other workflows were not successful. For instance, WF1718 had the third best scoring compounds (though not deemed novel) and WF1708 ranked third when rescreening Round 1 compounds (Figure 7). The binding features to the ADPr site of Nsp3-Mac1 were well documented at the outset of this challenge^8,12^ and the numerous structures with bound fragments in the PDB were used by all top seven teams.

**Figure 7:**
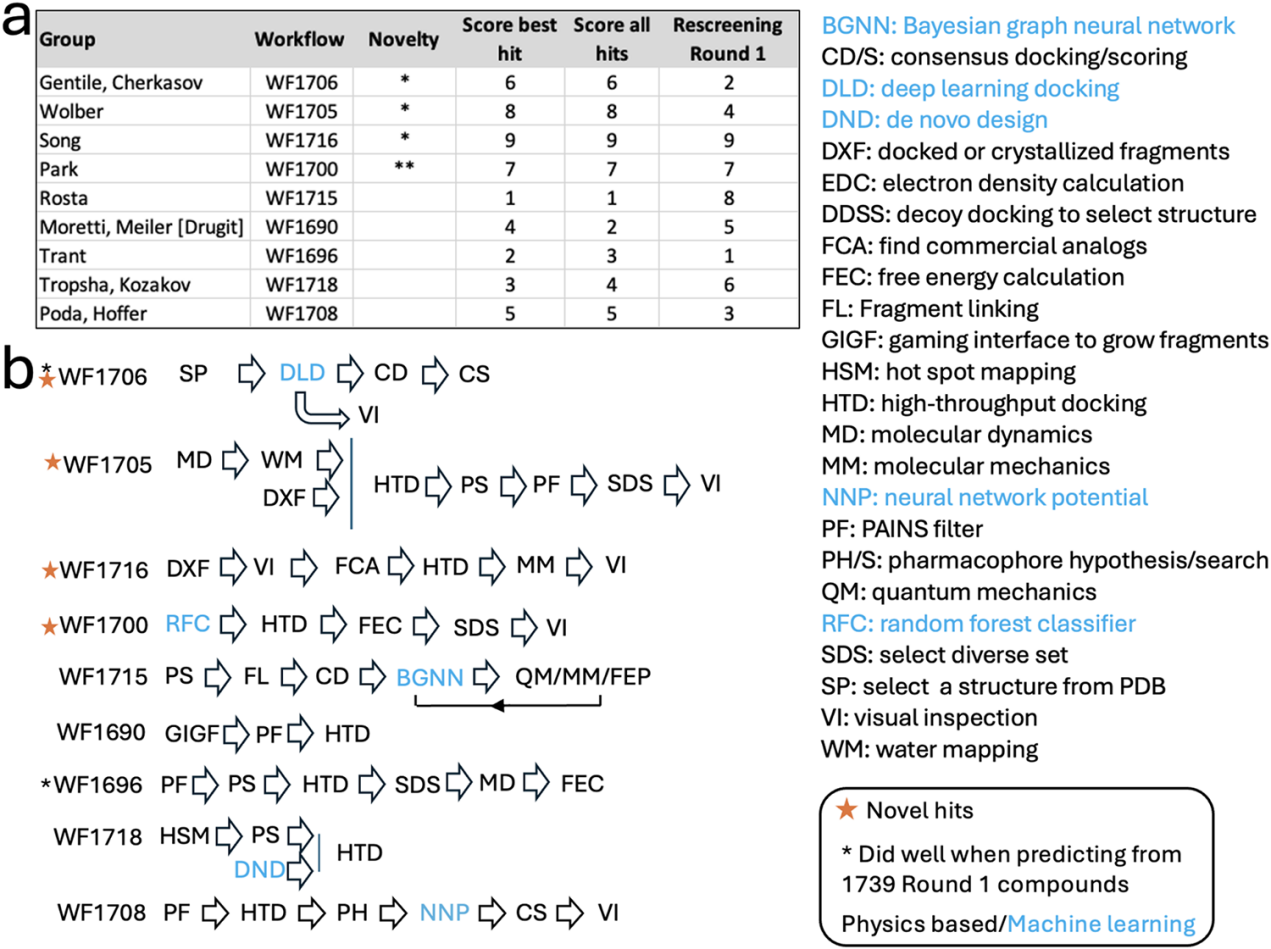
Best performing workflows. (a) Group, workflow ID, and associated ranks in three evaluation schemes. (b) Schematics of the computational workflows.

**Figure 8:**
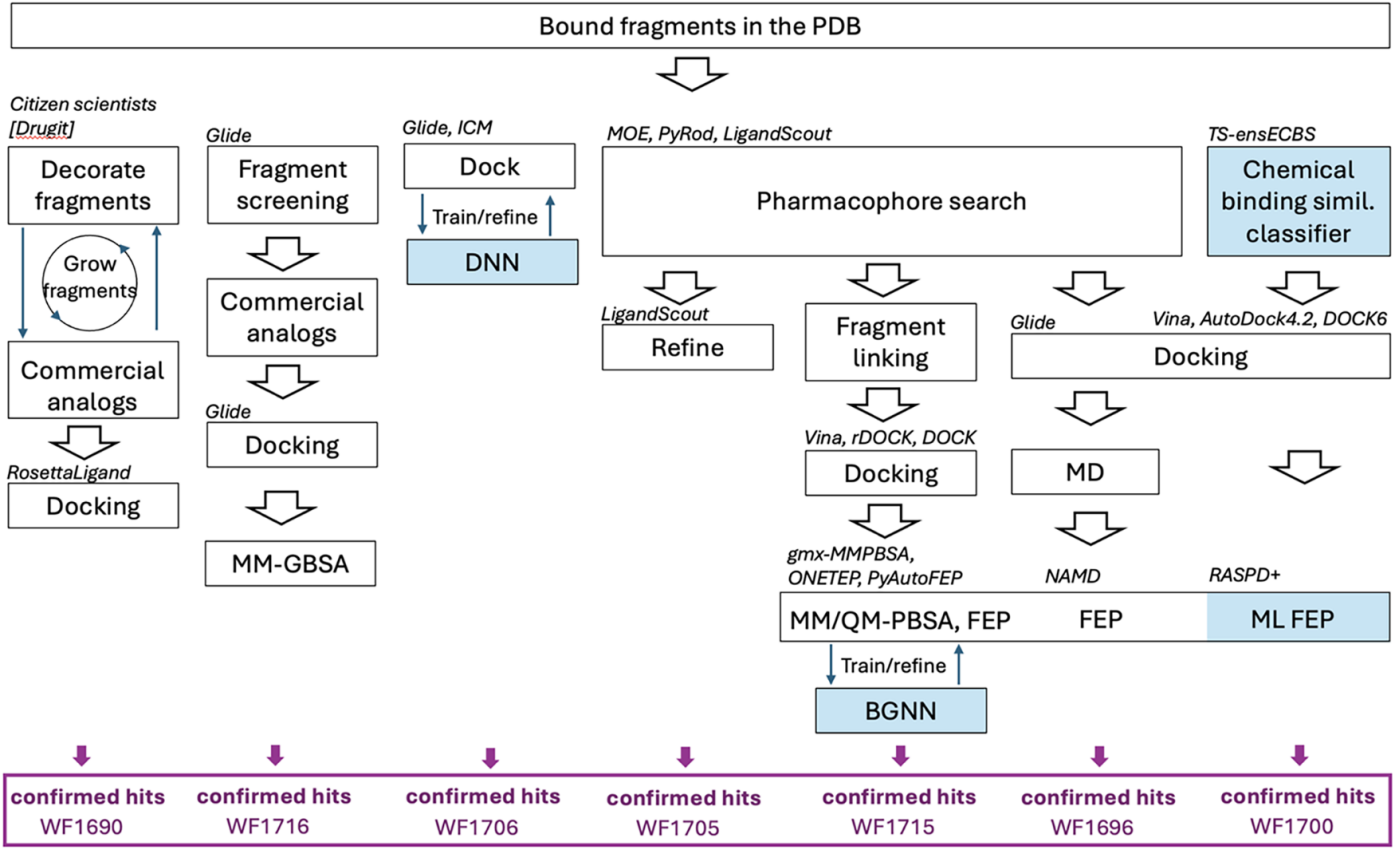
Classification of some of the most successful workflows in the CACHE #3 Challenge. Computational workflows are classified based on hit-prediction strategies. Computational steps using machine-learning are highlighted in blue. Software names are shown in italic.

Three of them used this information to guide a preliminary rapid pharmacophore search of the Enamine REAL database. Workflow WF1715 produced the most and the best scoring hits, though not novel: a pharmacophore search guided by ligands in the PDB retrieves commercial fragments that were combined based on both chemical and geometrical constraints, followed by consensus docking of assembled compounds with Vina,^16^ rDock,^17^ and DOCK v6.^18^ The full Enamine database was then screened with a Bayesian Graph Neural Network binding affinity predictor iteratively trained on a growing data set where, at each cycle, the absolute free energy of binding of the top few hundred molecules was calculated using gmx-MMPBSA,^19^ semiempirical QM/MMPBSA,^20^ and free energy perturbation with PyAutoFEP.^21^ WF1705, a purely physics-based approach, predicted CACHE3HI_1705_4, a compound with a very good score and judged sufficiently novel by two of the three chemists in the Hit Evaluation Committee (Tables S1 and S7): Here, MD-based water mapping and a pharmacophore search with PyRod^22^ and LigandScout was used to filter GOLD docking poses. WF1696, another robust, purely physics-based approach produced the second-best scoring compound. It also did better than any other method in retrieving hits from the collection of all molecules selected in Round 1 (Figure 6 and Table 1): following a filter to remove compounds with chemical liability alerts and carboxylic acids (as requested by the organizers), a rapid pharmacophore search was followed by high-throughput docking with Glide. Top scoring molecules were clustered and the conformational stability of a representative set was evaluated with MD simulations, followed by free energy perturbation with an extension of the NAMD2.14 molecular dynamics program.^23^

Another four workflows adopted different strategies to leverage Nsp3-bound fragments in the PDB. WF1690 relied on the gaming challenge Drugit within the online platform Foldit.^24^ Here, citizen scientists interactively grow fragments inside a 3D rendering of the binding pocket, employing a tool which leverages the ZINC API^25^ to then find the closest commercial analog. The resulting designs are then redocked with RosettaLigand,^26^ and ranked based on the quality of the redocking.

WF1716 and WF1706 produced the well-scoring CACHE3HI_1716_34 and CACHE3HI_1706_56, deemed mildly novel due to an amide group on the canonical adenine-mimicking bicyclic ring (Table 1 and Figure 5), but the workflows were drastically different: WF1716 designed novel compounds based on docked fragments. Commercial analogs were docked with Glide and evaluated with MM-GBSA to produce a final selection. WF1706 started with identifying Nsp3-Mac1 structures in the PDB that were suitable for virtual screening. The team’s Deep Docking protocol was then applied to the most promising complex, where Glide docking scores of thousands of compounds serve as the training set for a deep neural network that is then used to rapidly screen 23 million compounds from the Diversity Set of Enamine REAL Database.^14^ Visual inspection of poses, in parallel with physics-based consensus docking with ICM and Glide produced the refined selection.

Finally, WF1700 used a random forest classifier that had learned target-specific ensemble evolutionary chemical binding similarity (TS-ensECBS^27^) to identify commercial compounds likely to recapitulate atomic interactions of bound fragments in the PDB. This rapid preliminary screen was followed by high-throughput docking with Vina,^16^ AutoDock4.2, DOCK6 for consensus scoring, and rapid ML-based binding free energy prediction with RASPD+,^28^ leading to CACHE3HI_1700_52, the most novel hit, for which the follow-up compounds in Round 2 were unfortunately not close analogs.

## Discussion

Based on the observation that previously published Nsp3-Mac1 ligands all contained a canonical bicyclic system mimicking the adenine ring of the endogenous substrate or featured an undesired carboxylic acid,^8,12^ CACHE #3 proposed a dual design challenge: discovering drug-like ligands for Nsp3-MAC1 that (i) were chemically novel and (ii) did not include a carboxylic acid. Overall, participants met the second goal, but not the first. Two of the workflows delivering the most potent but not novel compounds employed design strategies that implied the preservation of chemical scaffolds already in the PDB: fragment growing (WF1690) and fragment linking (WF1716). But other workflows only indirectly leveraged structural data from previous compounds, for instance through pharmacophore searches (WF1696, WF1705, and WF1715), or structure selection based on previously cocrystallized small molecules (WF1706).

Overall, 85% of the Round 1 compounds selected by computational teams were indeed chemically novel, but 27 of the 28 molecules that advanced to Round 2 and all but one of the top-ranking compounds featured the canonical adenine mimic, suggesting that alternative chemotypes for this pocket, which was selected through evolution to recognize the adenine ring of ADPr, are rare. Some of the computational workflows deployed in CACHE #3 did not include machine learning, discounting the possibility that the only reason methods failed to find new chemotypes is a lack of adequate training data.

The crystal structures of a few compounds that did not contain the canonical scaffold were solved in complex with Nsp3-Mac1 (Figure 9). These compounds showed no measurable or weak binding by SPR (*K*_D_ > 200 μM), and all but one failed to recapitulate a previously described critical bidentate hydrogen-bond formed by the adenine mimetic with Asp22 and Ile23.^8,12^ This suggests that these binding poses, predicted computationally or observed fortuitously, are poor starting points for further optimization. The only exception is CACHE3HI_1879_22 (PDB code: 7HPV) where a thiazole-acetamide system preserved this critical dual hydrogen-bond, but binding was too weak to be quantified by SPR. This latter crystal structure suggests that Nsp3-Mac1-binding chemotypes distinct from the adenine-like scaffold exist in the Enamine collection, but that computational methods were not able to identify sufficiently potent compounds. This is an agreement with results from a previous fragment screen by crystallography that identified noncanonical chemotypes binding the adenine site but did not yield optimized ligands.^8^

**Figure 9:**
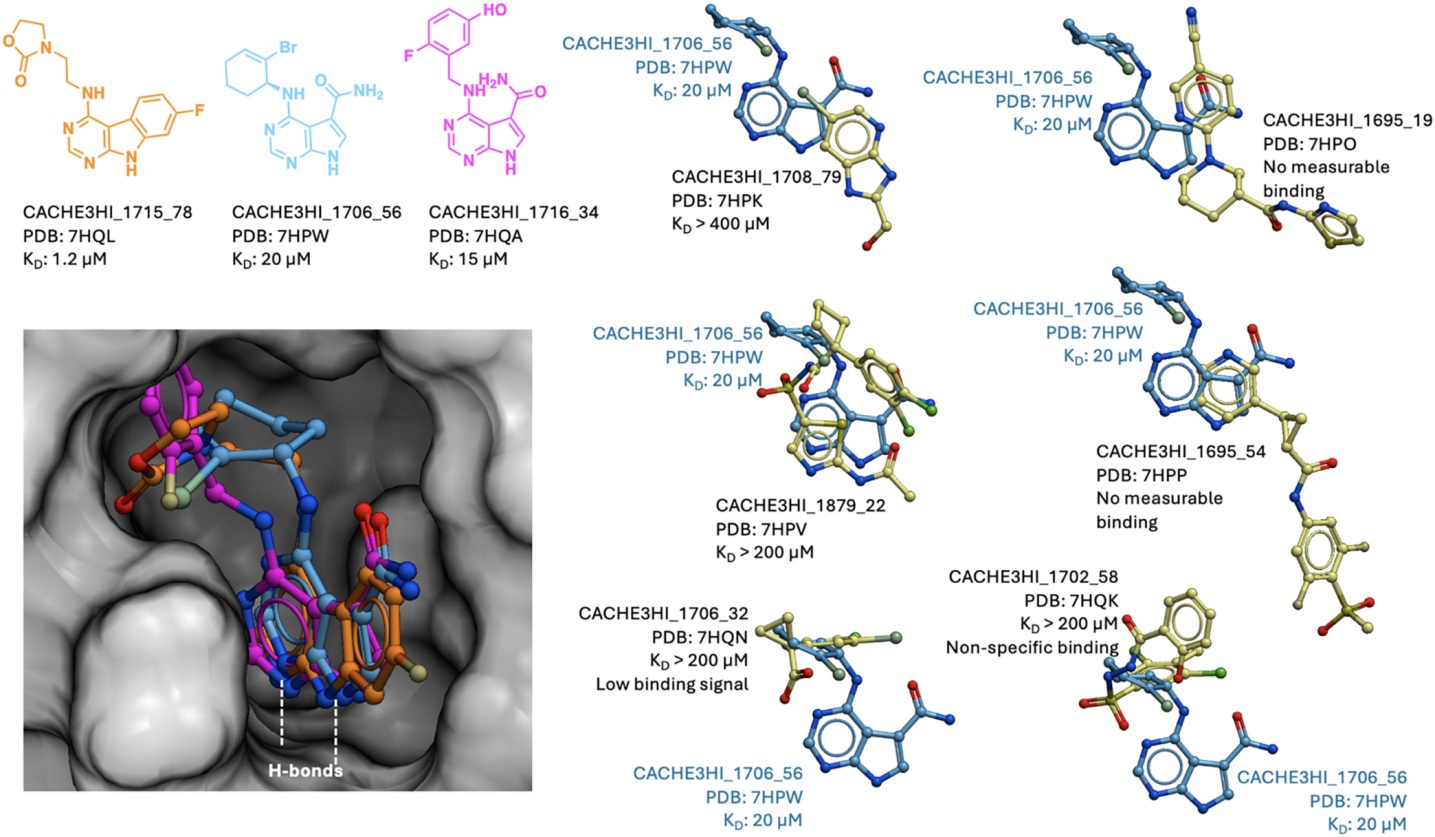
Crystal structures captured diverse chemotypes. The most potent CACHE #3 hits engage in a bidentate hydrogen-bond with Nsp3-Mac1 (left) also observed with the endogenous adenine ring. Weaker crystallized ligands typically lose this interaction (right).

Some of the successful workflows deployed in CACHE #3 incorporated methods and tools that were also successful in previous CACHE challenges. Deep docking,^14^ the platform where a million diverse compounds are screened with a physics-based docking algorithm to train a deep neural network that can then rapidly predict docking scores for billions of molecules, delivered experimentally confirmed hits for the WDR domain of LRRK2 (CACHE #1, WF1209) and for Nsp3-Mac1 (CACHE #3, WF1706). Similarly, training neural networks with docking scores from Glide (CACHE #1, WF1193), Vina (CACHE #2 WF1154), or GNINA^29^ (CACHE #2, WF1156) to rapidly screen large chemical libraries, sometimes within active learning cycles, performed well in previous challenges. Active learning cycles to iteratively improve neural networks with training data from computationally demanding binding free energy calculations was also successfully implemented to refine the selection of hit candidates in CACHE #1 (WF1209) and CACHE #3 (WF1715).

Generative models were successfully applied in four CACHE #1 workflows but only one in CACHE #3 and were absent in CACHE #2, which may reflect the fact that no ligand was known for LRRK2, the CACHE #1 target, while fragments or ligands were available in the PDB for Nsp13 (CACHE #2) and Nsp3-Mac1 (CACHE #3). Fragment-based techniques appear as versatile hit discovery tools in CACHE across a variety of targets as they were applied in 40% of the top workflows across the three challenges (Table 2).

**Table 2:** Successful Strategies across the First Three CACHE Challenges^a^

	CACHE #1	CACHE #2	CACHE #3
all top performing methods	7	6	7
machine learning	6	5	3
deep learning	5	5	2
active learning	1	2	2
generative models	4	0	1
fragment-based	3	2	3
pharmacophore	0	0	3

a Most workflows combined two or more of the listed strategies.

As in CACHE #2 (WF1414), a citizen scientist using the Drugit platform to grow fragments bound to the target structure designed an excellent compound in CACHE #3 (WF1690): CACHE3HI_1690_48 binds Nsp3-MAC1 with a *K*_D_ ∼ 10 μM (Table 1). This result is sobering, as design strategies developed by computational chemistry experts may not outperform the creation of online gamers (though Drugit participants may be experienced medicinal chemists), but also up-lifting, as so far, human neural networks are at least as creative as artificial ones. One should also keep in mind that the molecules invented by Drugit citizen scientists were further evaluated with specialized software (see above) before being submitted to CACHE #3.

Human judgment may also have played an important role in the final evaluation step of computationally selected molecules: while 78% (seven out of nine) of the top-performing teams explicitly specified in their workflows that visual inspection of the computationally selected molecules would be applied to finalize their hit list (Figure 7), only 43% (10 out of 23) of all CACHE #3 participants did so (Figure 2). While these numbers on their own are not sufficient to draw a clear conclusion, it will be interesting to see whether a similar trend takes shape across multiple CACHE challenges.

A trend that is new in CACHE #3 is the recurrent use of free energy perturbation techniques, implemented with GROMACS in PyAutoFEP^21^ (WF1715), with CHARMM in NAMD^23^ (WF1696), or predicted by a machine learning model in RASPD+r,^28^ to refine hit lists generated by high-throughput screening platforms. As access to GPUs increases, we expect that binding free energy calculations will become increasingly ubiquitous in molecular design, whether they are purely physics-based, or augmented by, or even replaced with, machine learning potentials.^30,31^

While the CACHE #3 challenge was ongoing, an independent hit-to-lead optimization effort led to AVI-4206, a potent and selective Nsp3-Mac1 ligand that reduces viral replication, potentiates innate immune response, leads to a survival benefit in animal models and has antiviral activity in human airway organoids. This strongly supports the hypothesis that Nsp3-Mac1 is a valid target for drugs that could restore the host innate immune response and be combined with existing or future COVID-19 drugs targeting viral assembly, replication or transcription.^10^ While CACHE #3 participants were constrained to select molecules only within the boundary of the (admittedly still vast) Enamine catalog, and overall failed to reveal novel chemical scaffolds, they did provide novel chemical insight around the canonical Nsp3-Mac1 targeting chemical template, which may inform the design and optimization of more advanced compounds.

## Conclusion

As with its predecessors, the third CACHE challenge reflects the engagement and diversity of a vibrant and creative computational drug design community. CACHE #3 highlights particularly how lightning-fast advances in machine learning can be exploited to sample the rapidly growing accessible chemistry space with increased speed and precision. Training artificial neural networks with synthetic data from physics-based virtual screens has been successfully implemented in all CACHE challenges so far. This strategy comes with the risk that approximate docking poses and scoring functions may lead to unacceptably noisy training data sets, and active ‘unlearning’ loops. It will be interesting to see whether increased availability of ever faster GPUs enables the deployment of more robust and accurate physics-based methods to produce cleaner data sets and train better-performing machine learning models. While this is one of the paths illuminated by CACHE challenges, CACHE #3 as its predecessors shows that, so far, machine learning augments but does not consistently outperform physics-based methods. Clearly, a breakthrough driven by artificial intelligence remains to be seen.

## Methods

### Protein Expression and Purification

DNA fragments encoding SARS-CoV-2 Nsp3Macro 1 domain (residues 205–380), Nsp3Macro 1 domain (206–374), and human PARP14 (residues 711–913) were subcloned into expression vectors with N-terminal His tags. Specifically, Nsp3 (205–380) was cloned into pDEST17, while Nsp3 (206–374) and PARP14 (residues 711–913) were inserted into pNicBio3 and pNIC-Bio2, respectively, with C-terminal AviTags for biotinylation.

Protein constructs were expressed in *Escherichia coli* BL21 (DE3) or BL21 (DE3)-BirA (for AviTag constructs) in Terrific Broth supplemented with antibiotics. Cultures were induced at OD_600_ 0.8–1.5 with 0.5 mM IPTG and incubated overnight at 18 °C. For AviTag constructs, D-Biotin (10–100 μM) was added to the media.

Cells were harvested and lysed in Tris-HCl buffer (pH 7.5) containing 500 mM NaCl, imidazole, glycerol, and a protease inhibitor cocktail (Aprotinin, Leupeptin, Pepstatin A, E-64). Chemical lysis was performed using CHAPS, TCEP, PMSF/Benzamidine, and Benzonase, followed by sonication (5–10 min, Sonicator 3000, Misoni). The lysates were clarified by centrifugation (36,000*g*, 60 min, 4 °C).

Purification was performed as follows: Nsp3 (205–380), Ni-NTA affinity chromatography eluted with imidazole, followed by gel filtration (HiLoad Superdex75 26/600, ÄKTA Pure) in 50 mM Tris (pH 7.5), 250 mM NaCl, 0.5 mM TCEP, and 5% glycerol; Nsp3 (206–374), Ni-NTA affinity chromatography with a prebiotin wash and eluted with imidazole, followed by gel filtration under the same conditions; and PARP14 (residues 711–913) Ni-NTA affinity chromatography with a prebiotin wash and eluted with imidazole, followed by dialysis in 20 mM Tris, pH 8, 500 mM NaCl, 1 mM TCEP.

All three proteins were purified to 95% purity, assessed by SDS-PAGE, pooled, concentrated, snap-frozen, and stored at −80 °C. Protein identity was confirmed by LC-MS.

### Homogeneous Time-Resolved Fluorescence (HTRF)

Binding affinity of the tested compounds to SARS2-CoV-2 Nsp3Mac1 (residues 205–380) protein was assessed by the displacement of an ADP-ribose conjugated biotin peptide from His6-tagged protein using HTRF-based assay. Compounds were dispensed into ProxiPlate-384 Plus (PerkinElmer) assay plates using an Echo 650 acoustic liquid handler (Beckman Coulter). The binding assay was conducted in a final volume of 20 μL with 12.5 nM NSP3Mac1 protein, 200 nM peptide ARTK­(Bio)­QTARK­(Aoa-RADP)­S (Cambridge Peptides), 0.031 nM Terbium-cryptate anti-His Mab (HTRF donor, PerkinElmer) and Streptavidin-XL665 (HTRF acceptor, PerkinElmer) in assay buffer (25 mM HEPES pH 7.0, 20 mM NaCl, 0.05% bovine serum albumin, and 0.05% Tween-20). Assay reagents were dispensed into plates using a Multidrop Combi (ThermoFisher Scientific). Macrodomain protein and peptide were first dispensed and incubated with the tested compounds for 30 min at room temperature, followed by addition of the HTRF reagents and incubation at room temperature for 1 h. Fluorescence was measured using a Synergy H1 microplate reader (Biotek) with the HTRF filter set (A = excitation 330/80 nm, emission of 620/10 nm, and B = excitation of 330/80 nm and emission of 665/8 nm). Obtained HTRF ratio values were used to estimate the percentage of ADP-r peptide binding inhibition/displacement using a positive control (4% DMSO solution in the presence of protein and peptide) and a negative control (4% DMSO solution in the presence of peptide) value from each screening plate.

### Surface Plasmon Resonance (SPR)

Orthogonal binding confirmation was assessed by SPR using streptavidin-conjugated (SA) chips. The assay was conducted using a Biacore 8K (Cytiva) instrument at 20 °C. Biotinylated SARS2-Cov2 NSP3 (residues 206–374) protein was immobilized onto the flow cell two of the SA chip following the manufacturer’s protocol reaching approximately 2800–3200 response units (RU). For the counter screen phase half of the channels were charged with the unrelated negative control protein PARP14 (residues 711–913) reaching approximately 2800–3000 RU. All flow cells were kept empty and served as a reference for subtraction for each channel. Compounds were initially dissolved in 100% DMSO to create 10 mM stock solutions, which were subsequently serially diluted (factor: 0.5) to obtain six concentration points in 100% DMSO. For the SPR run, these serially titrated compound stocks were diluted 1:25 in HBS-EP+ buffer (10 mM Hepes pH7.4, 150 mM NaCl, 3 mM EDTA, 0.05% (v/v) Tween20) supplemented with 0.5 mM reducing agent (TCEP) to achieve a final concentration of 4% DMSO. Binding experiments used multicycle kinetics with a contact time of 60 s and a dissociation time of 120 s at a flow rate of 40 μL/min at 20 °C. The dissociation constant (*K*_D_) values were determined using steady-state affinity 1:1 binding with the Biacore Insight Evaluation software (Cytiva).

### Dynamic Light Scattering

The solubility of compounds was estimated by DLS that directly measures compound aggregation via scattering intensity and size distribution. Compounds were prepared at 2.5 mM and 1.25 mM directly from DMSO stocks, then diluted 25× into filtered 10 mM Hepes pH7.4, 150 mM NaCl, 3 mM EDTA, 0.5 mM TCEP (4% DMSO final). The resulting samples were then distributed into 384-well plates (Corning, Cat# 3540), with 20 μL in each well. The sample plate was centrifuged at 3500 rpm for 5 min before loading into DynaPro DLS Plate Reader III (Wyatt Technology).

### Crystallography

The construct of Mac1 that crystallizes in P4_3_ was expressed, purified, and crystallized as described previously.^8^ Ligands prepared in DMSO at 50 mM were soaked into crystals achieving a nominal concentration of 5–10 mM using acoustic dispensing.^32^ After 2–4 h at room temperature, crystals were vitrified in liquid nitrogen with assistance from a Crystal Shifter.^33^ X-ray diffraction data were collected at beamline 8.3.1 (supplementary X-ray statistics). Data were indexed, integrated, and scaled using XDS^34^ and merged with Aimless.^35^ After initial rigid body refinement with phenix.refine,^36^ coordinates were refined using Refmac5 ^37^ as described previously.^11^ Ligands were identified and modeled using PanDDA^38^ run in CCP4 version 7.0.^39^ Ligand coordinates and restraints for refinement were generated with LigPrep (version 2022–1, Schrödinger, Inc.) and phenix.elbow^40^ or grade2 (version 1.7.0, Global Phasing Limited). Because of the low occupancy of many of the ligands, coordinates were refined using phenix.refine as a multistate model with the apo state assigned alternative location identified altloc A and the ligand-bound state assigned altloc B as described previously.^11^ Coordinates, structure factors, and PanDDA event maps have been deposited in the Protein Data Bank under group deposition G_1002327 with the following accession codes: 7HPI, 7HPJ, 7HPK, 7HPL, 7HPM, 7HPN, 7HPO, 7HPP, 7HPQ, 7HPR, 7HPS, 7HPT, 7HPU, 7HPV, 7HPW, 7HPX, 7HPY, 7HPZ, 7HQ0, 7HQ1, 7HQ2, 7HQ3, 7HQ4, 7HQ5, 7HQ6, 7HQ7, 7HQ8, 7HQ9, 7HQA, 7HQB, 7HQC, 7HQD, 7HQE, 7HQF, 7HQG, 7HQH, 7HQI, 7HQJ, 7HQK, 7HQL, 7HQM, 7HQN, 7HQO, and 7HQP.

## Supplementary Material







## Data Availability

All molecular structures and their properties and activities are available in Tables S2, S3, S5 and S6 in a machine-readable format. Software is open source or commercial and specified throughout the article and on https://cache-challenge.org/challenges/finding-ligands-targeting-the-macrodomain-of-sars-cov-2nsp3/computational-methods.

## Notes

SPR dose response curves, crystal structures and SAR of CACHE #3 compound, all CACHE #3 chemical series (PDF)
Composition of the CACHE Scientific Committees (Table S1); CACHE #3 Round 1 HTRF and DLS experimental screening data (Table S2); CACHE #3 Round 1 SPR dose response and counter-screening with solubility data (Table S3); CACHE #3 PDB codes (Table S4); CACHE #3 Round 2 HTRF three concentrations experimental screening data (Table S5); CACHE #3 Round 2 SPR dose response and counter-screening with solubility data (Table S6); annotations from the CACHE #3 hit evaluation committee (Table S7); scoring of computational workflows (Table S8) (XLSX)
X-ray statistics (XLSX)
