# Supplementary material for: CACHE Challenge #3: Targeting the Nsp3 Macrodomain of SARS-CoV‑2
Source: J Chem Inf Model. 2026 Jan 21;66(3):1566–81. doi: 10.1021/acs.jcim.5c02441 (PMC12892310; doi:10.1021/acs.jcim.5c02441)
Supplement: Supplementary file 1 [file ci5c02441_si_001.pdf]

CACHE Challenge #3: Targeting the Nsp3 Macrodomain of SARS-CoV-2

Oleksandra Herasymenko[1], Madhushika Silva[1], Galen J. Correy[2], Abd Al-Aziz A. Abu-Saleh[3,4], Suzanne Ackloo[1], Cheryl Arrowsmith[1,5,6], Alan Ashworth[7] Fuqiang Ban[8], Hartmut Beck[9], Kevin P. Bishop[10,11], Hugo J. Bohórquez[10,11], Albina Bolotokova[1], Marko Breznik[12], Irene Chau[1], Yu Chen[12], Artem Cherkasov[13], Wim Dehaen[14,15], Dennis Della Corte[16], Katrin Denzinger[12], Niklas P. Doering[12], Kristina Edfeldt[17], Aled Edwards[1], Darren Fayne[18,19], Francesco Gentile[20,21], Elisa Gibson[1], Ozan Gokdemir[22,23], Anders Gunnarsson[24], Judith Günther[25], John J. Irwin[26]Jan Halborg Jensen[27], Rachel J. Harding[1], Alexander Hillisch[28], Laurent Hoffer[10,11], Anders Hogner[29], Ashley Hutchinson[1], Shubhangi Kandwal[18,19,30,31], Andrea Karlova,[32] Kushal Koirala[33], Sergei Kotelnikov[34], Dima Kozakov[34] Juyong Lee[35,36,37], Soowon Lee[36], Uta Lessel[38], Sijie Liu[12], Xuefeng Liu[22,23], Peter Loppnau[1], Jens Meiler,[39,40,41], Rocco Moretti[41], Yurii S. Moroz[42,43] Charuvaka Muvva[44], Tudor I. Oprea[45], Brooks Paige[46], Amit Pandit[12,47], Keunwan Park[44], Gennady Poda[10,11,48], Mykola V. Protopopov[42,43] Vera Pütter[49], Rahul Ravichandran[20], Didier Rognan[50], Edina Rosta[51], Yogesh Sabnis[52], Thomas Scott[41], Almagul Seitova[1], Purshotam Sharma[3,4], François Sindt[50], Minghu Song[53],Casper Steinmann[54] Rick Stevens[22,23], Valerij Talagayev[12], Valentyna V. Tararina[43], Olga Tarkhanova[40] Damon Tingey[16],John F. Trant[3,4,55] Dakota Treleaven[56], Alexander Tropsha[33], Patrick Walters[57], Jude Wells[58], Yvonne Westermaier[59], Gerhard Wolber[12], Lars Wortmann[60], Shuangjia Zheng[61], James S. Fraser[62*] and Matthieu Schapira[1,63,64*]

[1]Structural Genomics Consortium, University Health Network, Toronto, Ontario M5G 2C4, Canada
[2] Department of Bioengineering and Therapeutic Sciences, University of California San Francisco, San Francisco, CA 94158, USA
[3]Department of Chemistry and Biochemistry, University of Windsor, 401 Sunset Avenue, Windsor, ON, Canada, N9B 3P4
[4]Binary Star Research Services, LaSalle, ON, N9J 3X8, Canada
[5]Department of Medical Biophysics, University of Toronto, Toronto, Ontario M5G 1L7, Canada
[6]Princess Margaret Cancer Centre, University Health Network, Toronto, Ontario M5G 2C4, Canada
[7]Helen Diller Family Comprehensive Cancer Center, University of California, San Francisco, San Francisco, CA, USA
[8]Vancouver Prostate Centre, 2660 Oak Street, Vancouver, British Columbia, V6H3Z6, Canada
[9]Bayer AG, Drug Discovery Sciences, Wuppertal, 42113, Germany
[10]Drug Discovery Program, Ontario Institute for Cancer Research, Toronto, Ontario M5G 0A3, Canada
[11]QuAccel, Toronto, Ontario, Canada
[12]Molecular Design Group, Institute of Pharmacy, Department of Biology, Chemistry & Pharmacy, Freie Universitaet Berlin, Koenigin-Luisestr. 2+4, 14195 Berlin, Germany
[13]University of British Columbia, Vancouver, British Columbia V6H 3Z6, Canada
[14]CZ-OPENSCREEN, Department of Informatics and Chemistry, Faculty of Chemical Technology, University of Chemistry and Technology Prague, Technická 5, 16 628 Prague 6, Czech Republic
[15]Department of Organic Chemistry, Faculty of Chemical Technology, University of Chemistry and Technology Prague, Technická 5, 16 628 Prague 6, Czech Republic
[16]Department of Physics and Astronomy, Brigham Young University, Provo, Utah
[17]Structural Genomics Consortium, Department of Medicine, Karolinska University Hospital and Karolinska Institutet, Stockholm 171 76, Sweden
[18]DCU Life Sciences Institute, Dublin City University, Dublin, D09 DXA0, Ireland
[19]Molecular Design Group, School of Chemical Sciences, Dublin City University, Glasnevin, Dublin D09 V209, Ireland
[20]Department of Chemistry and Biomolecular Sciences, University of Ottawa, Ottawa, ON K1N 6N5, Canada

[21]Ottawa Institute of Systems Biology, Ottawa, ON K1H 8M5, Canada
[22]University of Chicago, Chicago, IL, USA
[23]Argonne National Laboratory, Lemont, IL, USA
[24]Protein, Structure and Biophysics, Discovery Sciences, BioPharmaceuticals R&D, AstraZeneca, Gothenburg, Sweden
[25]Bayer AG, Drug Discovery Sciences, Berlin 13353, Germany
[26] Department of Pharmaceutical Chemistry, University of California San Francisco, 1700 4th St, San Francisco California 94158-2330, United States
[27]Department of Chemistry, University of Copenhagen, Copenhagen, Denmark
[28]UCB BioSciences GmbH, Rolf-Schwarz-Schütte-Platz 1, 40789 Monheim am Rhein, Germany
[29]Medicinal Chemistry, Research and Early Development, Cardiovascular, Renal and Metabolism (CVRM), BioPharmaceuticals R&D, AstraZeneca, Gothenburg, Sweden
[30]Molecular Design Group, School of Biochemistry and Immunology, Trinity Biomedical Sciences Institute, Trinity College Dublin, 152-160 Pearse St, Dublin 2, D02 R590, Ireland
[31]Trinity Biomedical Sciences Institute, School of Biochemistry and Immunology, Trinity College Dublin, 152-160 Pearse Street, Dublin 2, D02 R590, Ireland
[32] Department of Computer Science, University College London
[33]Eshelman School of Pharmacy, The University of North Carolina at Chapel Hill, Chapel Hill, North Carolina, USA
[34] Stony Brook University, Department of Applied Mathematics & Statistics, Stony Brook, NY 11794-3600.
[35]Department of Molecular Medicine and Biopharmaceutical Sciences, Seoul National University, 08826, Seoul, South Korea
[36]College of Pharmacy, Seoul National University, 08826, Seoul, South Korea
[37]Arontier co., 06784, Seoul, South Korea
[38]Boehringer Ingelheim Pharma GmbH & Co. KG, Birkendorfer Str. 65, 88397 Biberach an der Riss, Germany
[39]Institute for Drug Discovery, Faculty of Medicine, Faculty of Mathematics and Informatics, Faculty of Chemistry and Mineralogy, University Leipzig, Leipzig, Germany

[40]Center for Scalable Data Analytics and Artificial Intelligence ScaDS.AI Dresden/Leipzig and School of Embedded Composite Artificial Intelligence SECAI, Dresden/Leipzig, Germany
[41]Center for Structural Biology, Vanderbilt University, South Nashville, Nashville, Tennessee 37240-0002, United States of America
[42]Chemspace, Kyiv 02094, Ukraine
[43]Taras Shevchenko National University of Kyiv, Kyiv, Ukraine
[44]Center for Natural Product Systems Biology, Korea Institute of Science and Technology, Gangneung 25451, Republic of Korea
[45]Experts System Inc.,12730 High Bluff Drive, Suite 100, San Diego, CA 92130, United States
[46]AI Centre, Department of Computer Science, University College London
[47] School of Pharmacy and Technology Management. SVKM's Narsee Monjee Institute of Management Studies (NMIMS) Deemed University, Indore, Madhya Pradesh 453112, India
[48]Leslie Dan Faculty of Pharmacy, University of Toronto, Toronto, Ontario M5S 3M2, Canada
[49]Nuvisan ICB GmbH, Berlin 13353, Germany
[50]Laboratoire d'innovation thérapeutique, UMR7200 CNRS-Université de Strasbourg, 67400 Illkirch, France
[51]Department of Physics and Astronomy, University College London, London WC1E 6BT, United Kingdom
[52]UCB Pharma, Braine-L'Alleud, Belgium
[53]Institute of Health and Medicine, Hefei Comprehensive National Science Center, Hefei, Anhui, China
[54]Department of Chemistry and Bioscience, Aalborg University, Fredrik Bajers Vej 7H, DK-9230, Denmark
[55]WE-SPARK Health Institute, 401 Sunset Avenue, Windsor, ON, Canada, N9B 3P4
[56]Conscience Medicines Network, Toronto, Ontario M5G 1L7 Canada
[57]Relay Therapeutics, Cambridge, Massachusetts 02141, United States
[58]Department of Computer Science, University College London, London WC1E 6BT, United Kingdom
[59]Boehringer Ingelheim RCV, Dr. Boehringer-Gasse 5-11, 1121 Vienna, Austria
[60]Boehringer Ingelheim Pharma GmbH & Co. KG, Birkendorfer Str. 65, Biberach an der Riss 88397, Germany

[61]Shanghai Jiao Tong University, Shanghai 200030, China
[62]Department of Bioengineering and Therapeutic Sciences, University of California San Francisco, San Francisco, CA 94158, USA
[63]Princess Margaret Cancer Centre, University Health Network, Toronto, Ontario M5G 2C4, Canada
[64]Department of Pharmacology & Toxicology, University of Toronto, Toronto, Ontario M5S 1A8, Canada

*jfraser@fraserlab.com
*matthieu.schapira@utoronto.ca

[illegible]Department of Pharmacology & Toxicology, University of Toronto, Toronto, Ontario M5S 1A8, Canada

*jfraser@fraserlab.com
*matthieu.schapira@utoronto.ca

SPR dose response curves, crystal structures and SAR of CACHE #3 compounds ……... p.6 [this document]

Summary of crystal soaking, data collection and PanDDA analysis for X-ray datasets…. File “Supplementary Xray statistics” -A
Summary of ligand modeling …………………………………………………………………………… File “Supplementary Xray statistics” -B
X-ray data collection and refinement statistics ………………………………………………….. File “Supplementary Xray statistics” –C
CACHE #3 Committees …………………………………………………………………………………….. File “Supplementary Tables” – S1
CACHE3 Round 1 HTRF and DLS experimental screening ………………………………………. File “Supplementary Tables” – S2
CACHE3 Round 1 SPR dose response and counter-screening with solubility data …..…. File “Supplementary Tables” – S3
CACHE3 PDB codes ……………………………………………………………………………………………. File “Supplementary Tables” – S4
CACHE3 Round 2 HTRF three concentrations experimental screening…………..…………. File “Supplementary Tables” – S5
CACHE3 Round 2 SPR dose response and counter-screening with solubility data …..…. File “Supplementary Tables” – S6
Annotated hit evaluation form from the Hit Evaluation Committee ……………………….. File “Supplementary Tables” – S7
Aggregated scores of CACHE3 computational workflows ………………………………………. File “Supplementary Tables” – S8

SPR dose response curves, crystal structures and SAR of CACHE #3 compounds

CACHE#3 – SARS-CoV2 Nsp3 macrodomain
Participant 1690

9 analogs, including a parent molecule of <u>CACHE3HI_1690_36 chemotype</u> were submitted for round 2.
8 compounds, including a re-supplied parent molecule showed a dose depended displacement of ADP-ribose peptide by HTRF. **5 compounds** showed a dose dependent binding affinity by SPR.

PARENT MOLECULE

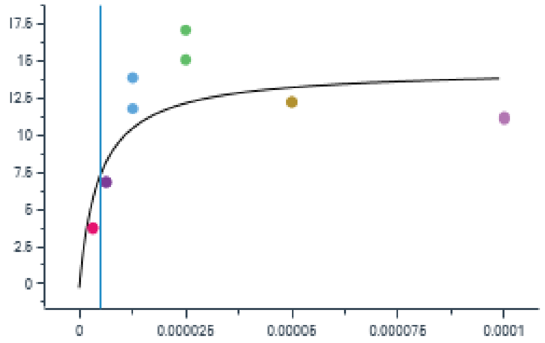

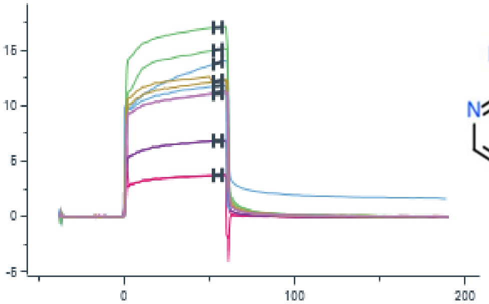

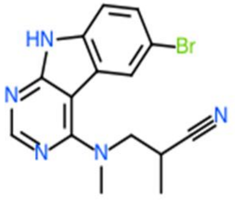

CACHE3HI_1690_36
K_D = 5 µM (poor fit) – 47% binding
Selectivity for NSP3 (against PARP14a protein) – Yes
DLS (solub@100 µM)
HTRF_displacement:
%inh@100 uM = 83
%inh@50 uM = 78
%inh@25 uM = 67

CACHE3-HO_1690_45
K_D = 2 µM – 37% binding
DLS (solub@100 µM)
HTRF_displacement hit confirmation (4% DMSO):
%inh@100 uM = 79
%inh@30 uM = 41
%inh@10 uM = 12

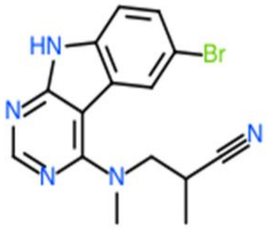

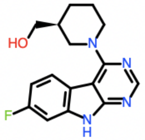

Closest published hit
dist: 0.22
Ki:78 µM

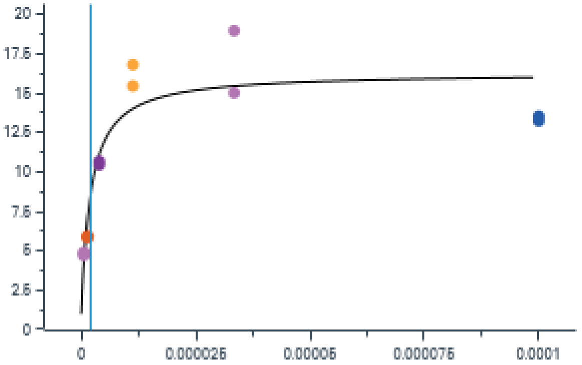

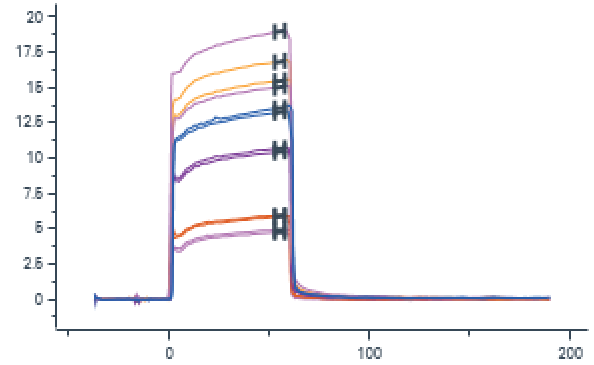

Tested analogs

confirmed

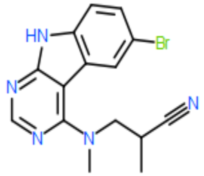

CACHE_ID CACHE3-HO_1690_45
Parent CACHE3HI_1690_36
distance 0

confirmed

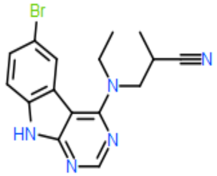

CACHE_ID CACHE3-HO_1690_18
Parent CACHE3HI_1690_36
distance 0.025

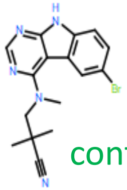

confirmed

CACHE_ID CACHE3-HO_1690_11
Parent CACHE3HI_1690_36
distance 0.02941

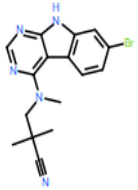

CACHE_ID CACHE3-HO_1690_29
Parent CACHE3HI_1690_36
distance 0.05417

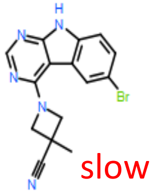

slow on/off

CACHE_ID CACHE3-HO_1690_32
Parent CACHE3HI_1690_36
distance 0.06122

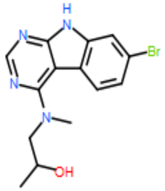

CACHE_ID CACHE3-HO_1690_23
Parent CACHE3HI_1690_36
distance 0.09796

confirmed

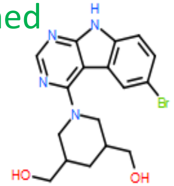

CACHE_ID CACHE3-HO_1690_16
Parent CACHE3HI_1690_36
distance 0.166

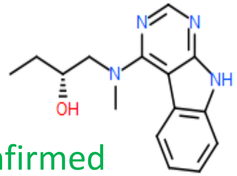

confirmed

CACHE_ID CACHE3-HO_1690_36
Parent CACHE3HI_1690_36
distance 0.176

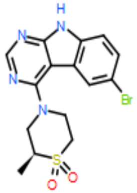

CACHE_ID CACHE3-HO_1690_14
Parent CACHE3HI_1690_36
distance 0.2058

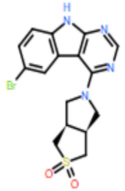

CACHE_ID CACHE3-HO_1690_35
Parent CACHE3HI_1690_36
distance 0.2755

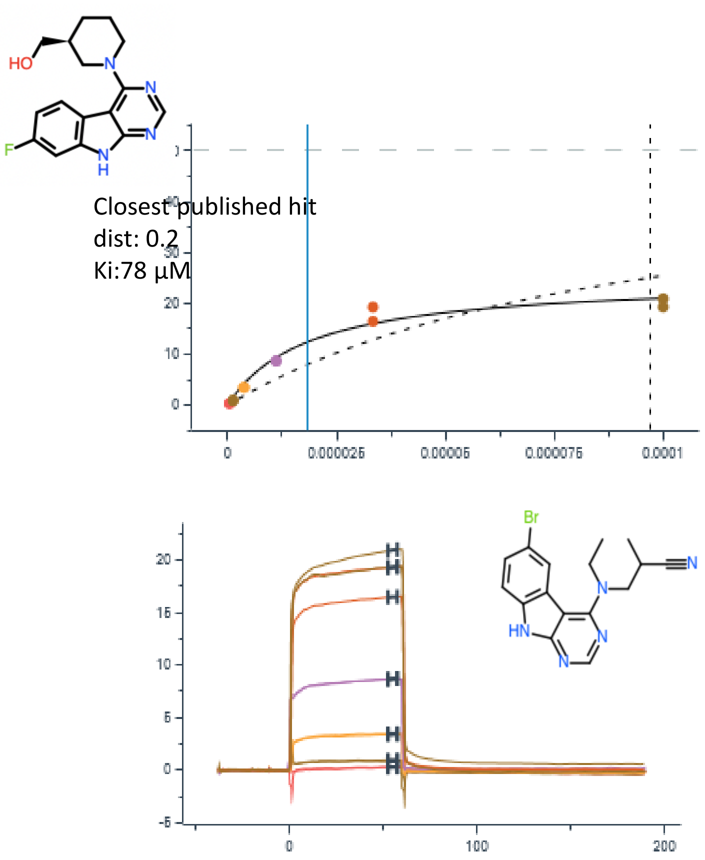

CACHE3-HO_1690_18

K_D = 18 µM – 50% binding
Selectivity for NSP3 (against PARP14a protein) – mild binding (slow off)
DLS (solub@100 µM)
HTRF_displacement hit confirmation (4% DMSO):
%inh@100 uM = 66
%inh@30 uM = 11
%inh@10 uM = 7

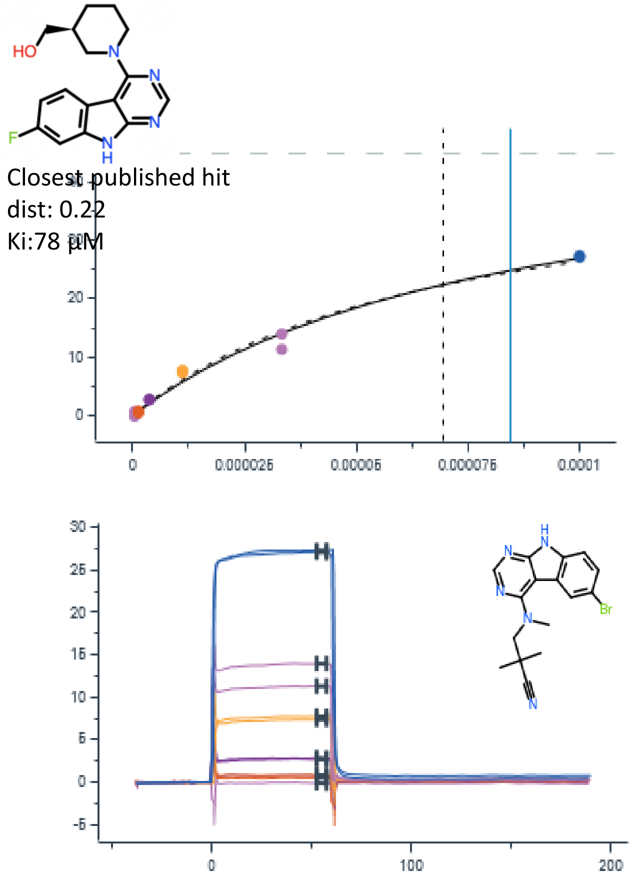

CACHE3-HO_1690_11

K_D = 85 µM – 111% binding/Saturation above 100 µM
Selectivity for NSP3 (against PARP14a protein) – Yes
DLS (solub@100 µM)
HTRF_displacement hit confirmation (4% DMSO):
%inh@100 uM = 82
%inh@30 uM = 66
%inh@10 uM = 20

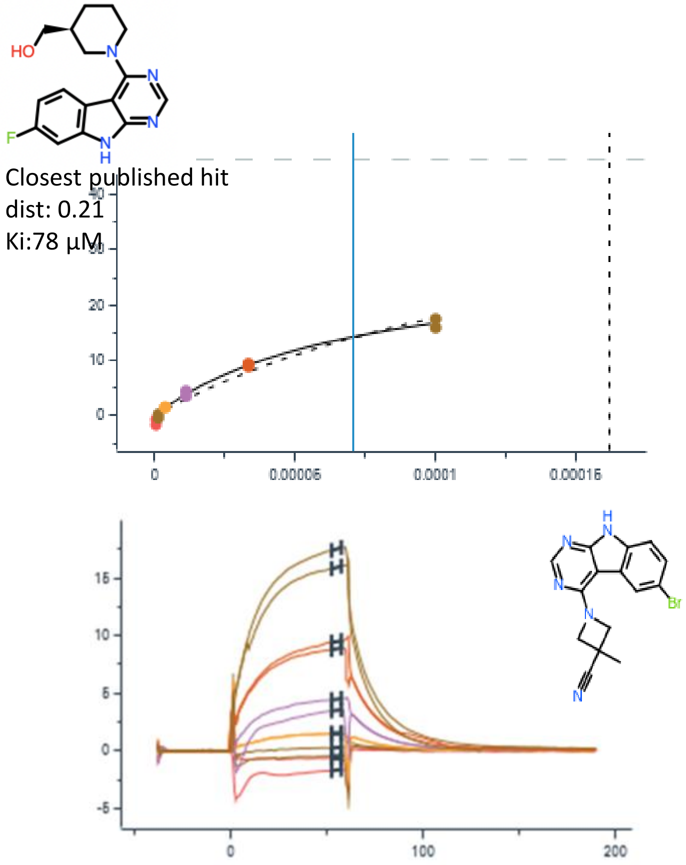

CACHE3-HO_1690_32

K_D = 71 µM (slow on/off) – 62% binding
Selectivity for NSP3 (against PARP14a protein) – 21% binding
DLS (solub@100 µM)
HTRF_displacement hit confirmation (4% DMSO):
%inh@100 uM = 83
%inh@30 uM = 74
%inh@10 uM = 44

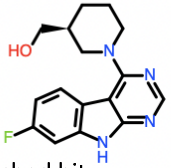

Closest published hit
dist: 0.08
Ki:78 µM

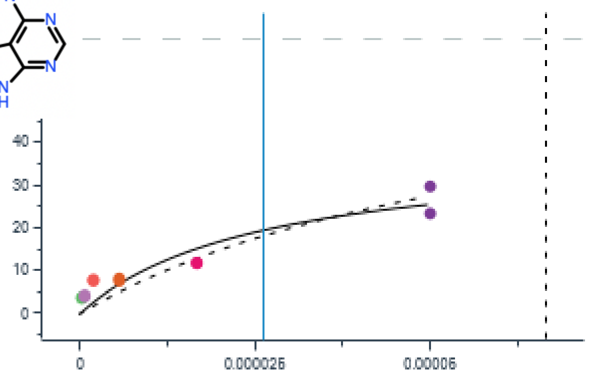

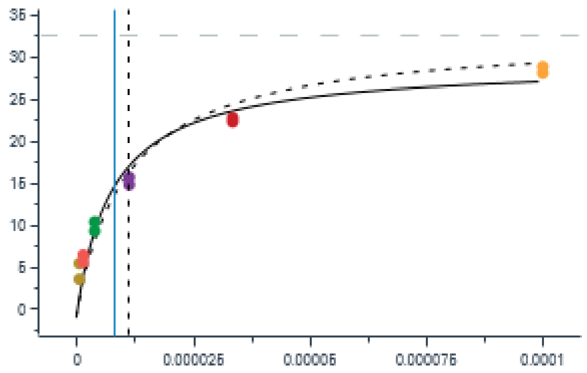

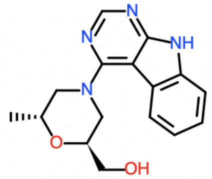

Closest published hit
dist:0.17
Ki:362 µM

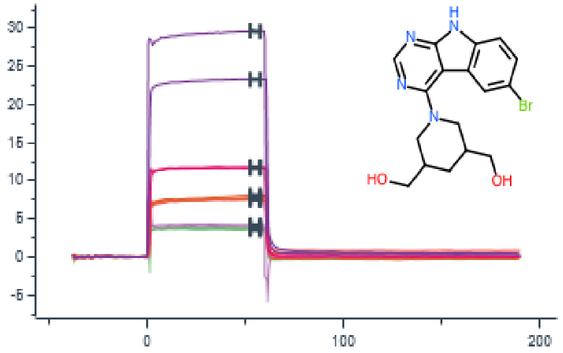

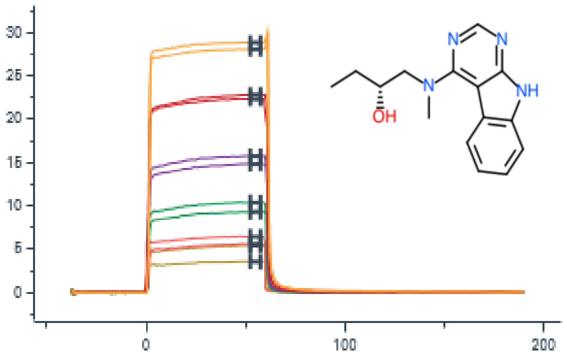

CACHE3-HO_1690_16

K_D (run 1) = 26 µM – 61%
K_D (run 2) = 55 µM – 110% binding
Selectivity for NSP3 (against PARP14a protein) – Yes
DLS (solub@50 µM)
HTRF_displacement hit confirmation (4% DMSO):
%inh@100 uM = 29
%inh@30 uM = 12
%inh@10 uM = 9

CACHE3-HO_1690_36

K_D = 8 µM – 90% binding
Selectivity for NSP3 (against PARP14a protein) – Yes
DLS (solub@100 µM)
HTRF_displacement hit confirmation (4% DMSO):
%inh@100 uM = 58
%inh@30 uM = 28
%inh@10 uM = 26

Round 1 HIT MOLECULE that was not followed up for round 2

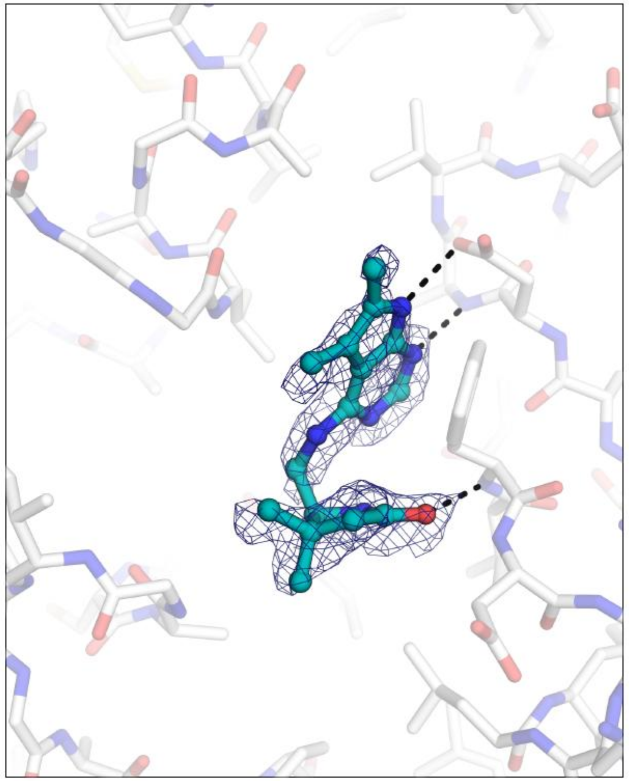

Blue mesh: PanDDA event map contoured at 2 σ

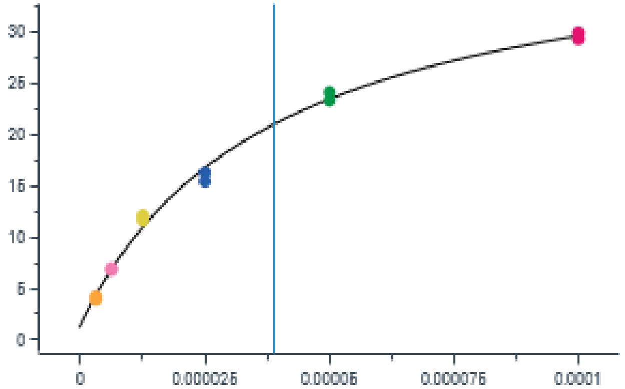

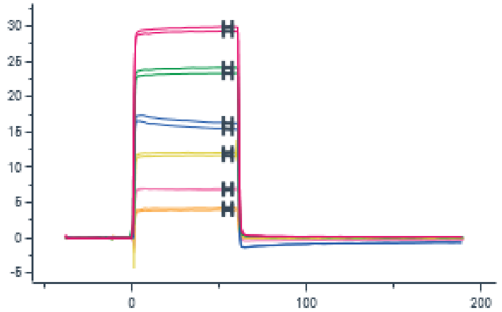

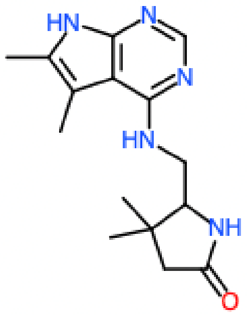

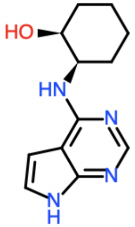

Closest published hit
dist: 0.35
Ki:114 µM

CACHE3HI_1690_45

K_D (run 1) = 39 µM – 111% binding
K_D (run 2) = 33 µM – 105% binding
Selectivity for NSP3 (against PARP14a protein) – Yes
DLS (solub@100 µM)
HTRF_displacement:
%inh@100 uM = 60
%inh@50 uM = 40
%inh@25 uM = 28

Structure – Yes !

PARENT MOLECULE

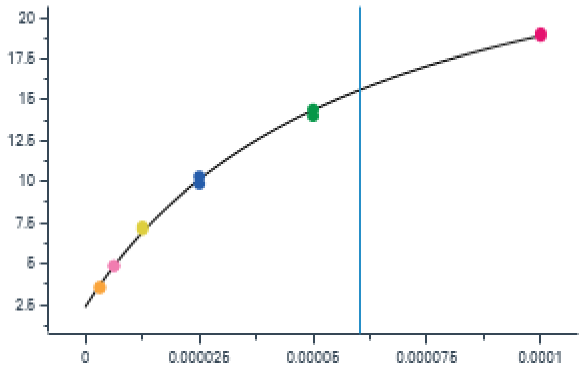

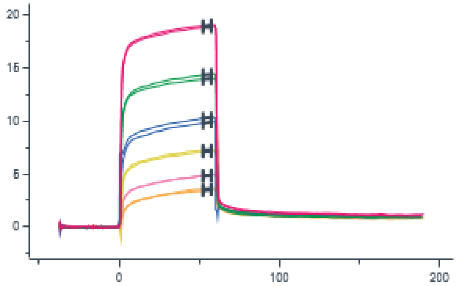

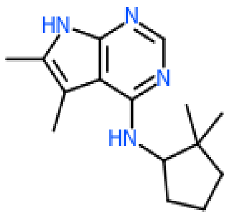

CACHE3HI_1690_48

K_D = 60 µM – 87% binding
Selectivity for NSP3 (against PARP14a protein)
DLS (solub@100 µM)
HTRF_displacement:
%inh@100 uM = 47
%inh@50 uM = 34
%inh@25 uM = 18

Structure – Yes !

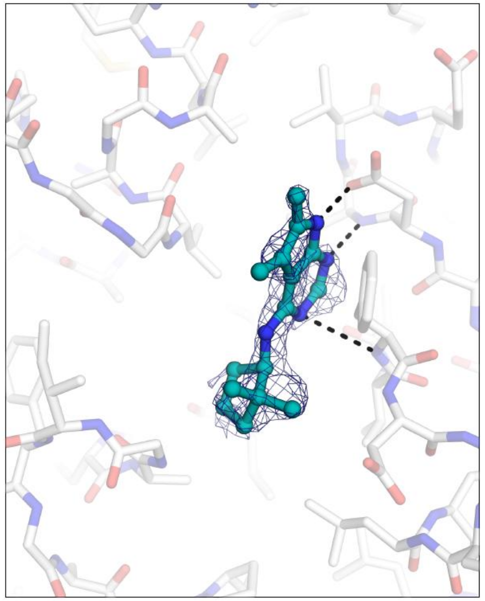

Blue mesh: PanDDA event map contoured at 2 σ

19 analogs, including a parent molecule, of CACHE3HI_1690_48 chemotype were submitted for round 2.
5 compounds, including a re-supplied parent molecule showed a dose depended displacement of ADP-ribose peptide by HTRF. Among them **3 compounds** confirmed binding affinity by SPR.

CACHE3-HO_1690_46

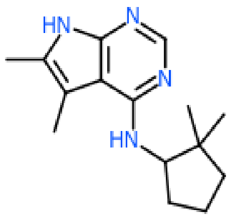

K_D = 84 µM – 145% binding
DLS (solub@30 µM)
HTRF_displacement hit confirmation (4% DMSO):
%inh@100 uM = 58
%inh@30 uM = 29
%inh@10 uM = 15

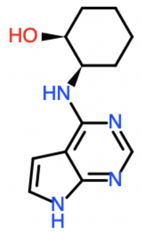

Closest published hit
dist: 0.26
Ki:114 µM

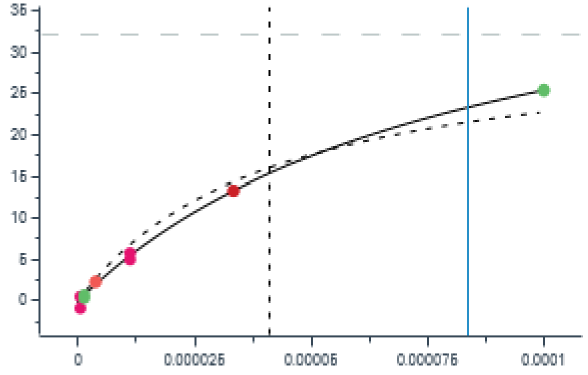

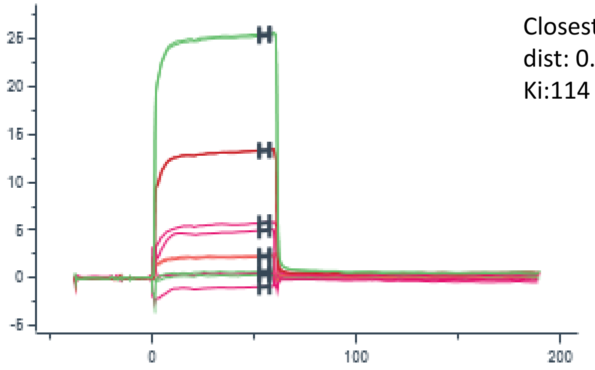

Tested analogs

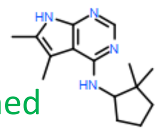<br>confirmed<br>CACHE_ID CACHE3-HO_1690_46<br>Parent CACHE3HI_1690_48<br>distance 0	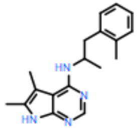<br>CACHE_ID CACHE3-HO_1690_20<br>Parent CACHE3HI_1690_48<br>distance 0.1614	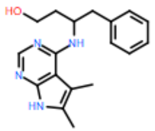<br>CACHE_ID CACHE3-HO_1690_2<br>Parent CACHE3HI_1690_48<br>distance 0.1818	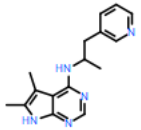<br>CACHE_ID CACHE3-HO_1690_13<br>Parent CACHE3HI_1690_48<br>distance 0.1842	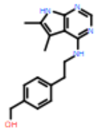<br>CACHE_ID CACHE3-HO_1690_7<br>Parent CACHE3HI_1690_48<br>distance 0.1982
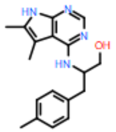<br>CACHE_ID CACHE3-HO_1690_28<br>Parent CACHE3HI_1690_48<br>distance 0.2	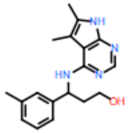<br>CACHE_ID CACHE3-HO_1690_26<br>Parent CACHE3HI_1690_48<br>distance 0.2009	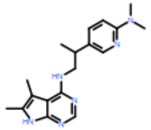<br>CACHE_ID CACHE3-HO_1690_6<br>Parent CACHE3HI_1690_48<br>distance 0.2152	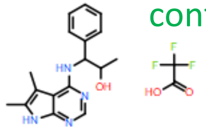<br>confirmed<br>CACHE_ID CACHE3-HO_1690_15<br>Parent CACHE3HI_1690_48<br>distance 0.2227	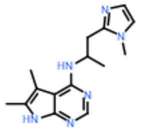<br>CACHE_ID CACHE3-HO_1690_31<br>Parent CACHE3HI_1690_48<br>distance 0.2324
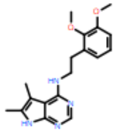<br>CACHE_ID CACHE3-HO_1690_8<br>Parent CACHE3HI_1690_48<br>distance 0.2581	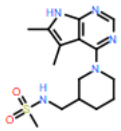<br>CACHE_ID CACHE3-HO_1690_17<br>Parent CACHE3HI_1690_48<br>distance 0.3089	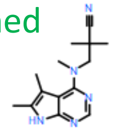<br>confirmed<br>CACHE_ID CACHE3-HO_1690_25<br>Parent CACHE3HI_1690_48<br>distance 0.3133	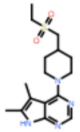<br>CACHE_ID CACHE3-HO_1690_30<br>Parent CACHE3HI_1690_48<br>distance 0.3191	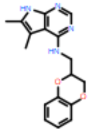<br>CACHE_ID CACHE3-HO_1690_5<br>Parent CACHE3HI_1690_48<br>distance 0.3309
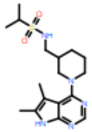<br>CACHE_ID CACHE3-HO_1690_10<br>Parent CACHE3HI_1690_48<br>distance 0.3346	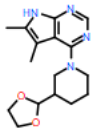<br>CACHE_ID CACHE3-HO_1690_27<br>Parent CACHE3HI_1690_48<br>distance 0.3346	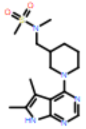<br>CACHE_ID CACHE3-HO_1690_4<br>Parent CACHE3HI_1690_48<br>distance 0.3383	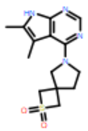<br>CACHE_ID CACHE3-HO_1690_22<br>Parent CACHE3HI_1690_48<br>distance 0.3741	

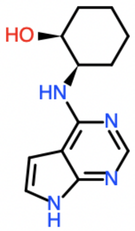

Closest published hit
dist: 0.28
Ki:114 µM

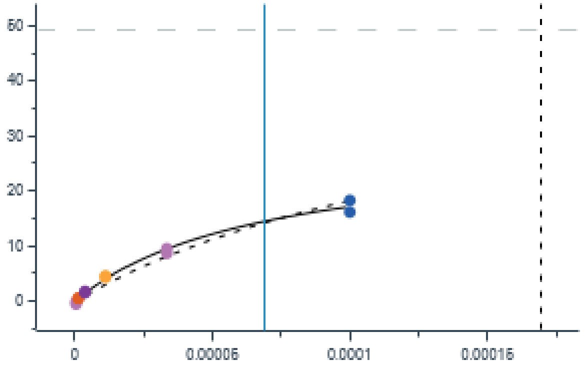

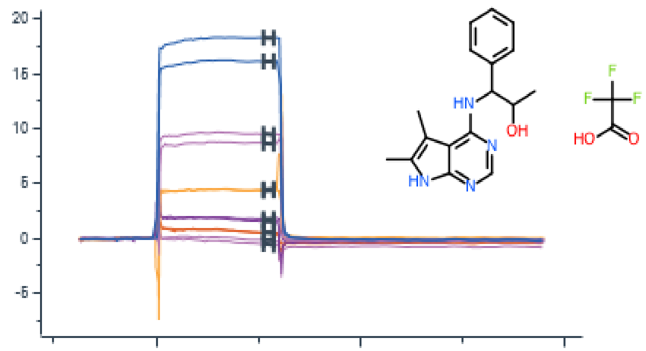

CACHE3-HO_1690_15

K_D = 69 µM – 59% binding

Selectivity for NSP3 (against PARP14a protein) – Yes

DLS (solub@100 µM)

HTRF_displacement hit confirmation (4% DMSO):

%inh@100 uM = 29

%inh@30 uM = 8

%inh@10 uM = 3

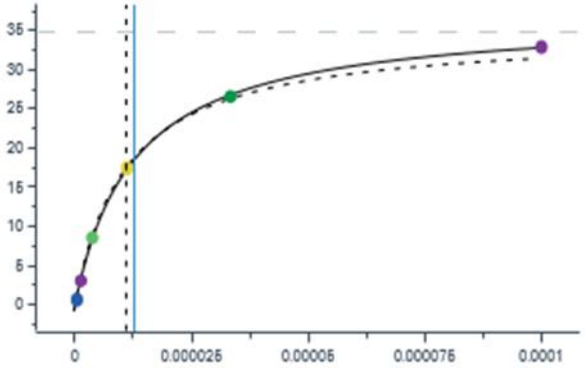

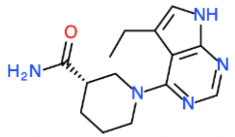

Closest published hit
dist: 0.28
Ki:177 µM

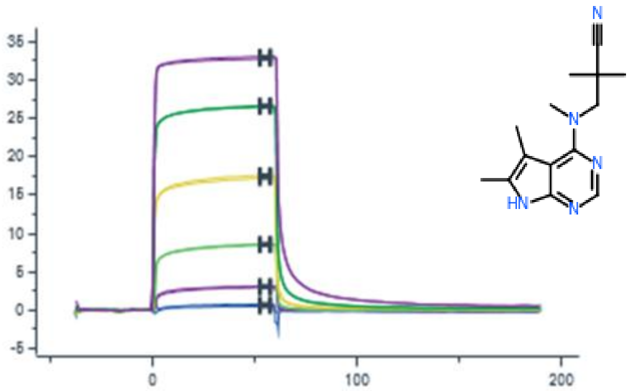

CACHE3-HO_1690_25

K_D (run 1) = 6 µM – 80% binding

K_D (run 2) = 13 µM – 106% binding

Selectivity for NSP3 (against PARP14a protein) – Yes

DLS (solub@100 µM)

HTRF_displacement hit confirmation (4% DMSO):

%inh@100 uM = 81

%inh@30 uM = 52

%inh@10 uM = 29

PARENT MOLECULE

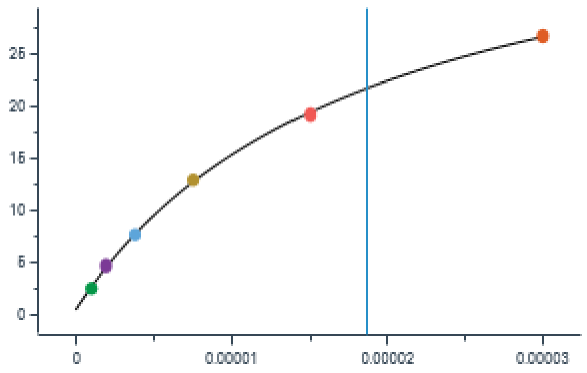

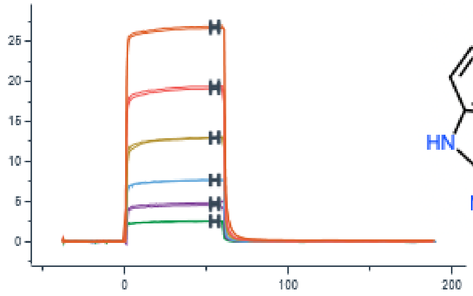

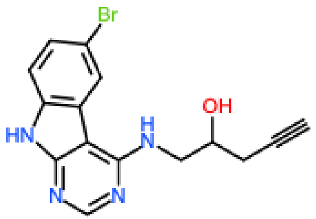

CACHE3HI_1690_63

K_D = 19 µM – 98% binding

Selectivity for NSP3 (against PARP14a protein) – Yes

DLS (solub@100 µM)

HTRF_displacement:

%inh@100 uM = 87

%inh@50 uM = 43

%inh@25 uM = 20

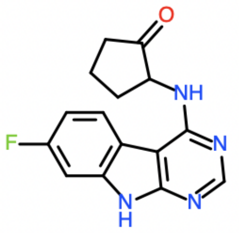

Closest published hit
dist: 0.21
Ki:11 µM

10 analogs, including a parent molecule, of CACHE3HI_1690_63 chemotype were submitted for round 2.

9 compounds, including a re-supplied parent molecule showed a dose depended displacement of ADP-ribose peptide by HTRF and binding response by SPR. Among them **7 compounds** confirmed binding affinity by SPR.

CACHE3-HO_1690_47

K_D = 36 µM – 102% binding

DLS (solub@100 µM)

HTRF_displacement hit confirmation (4% DMSO):

%inh@100 uM = 44

%inh@30 uM = 13

%inh@10 uM = 9

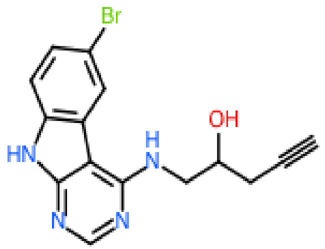

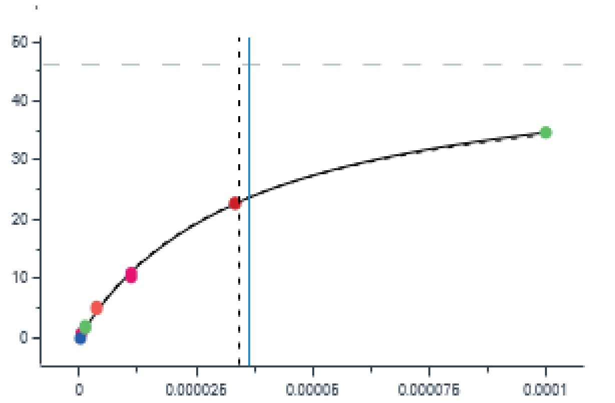

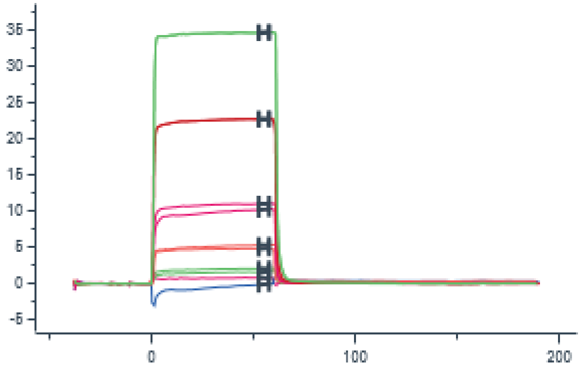

Tested analogs

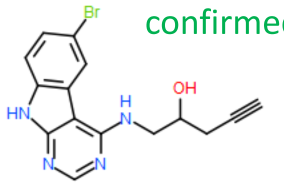

confirmed

CACHE_ID CACHE3-HO_1690_47
Parent CACHE3HI_1690_63
distance 0

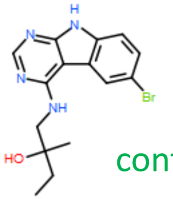

confirmed

CACHE_ID CACHE3-HO_1690_43
Parent CACHE3HI_1690_63
distance 0.08032

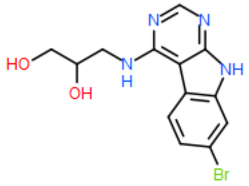

CACHE_ID CACHE3-HO_1690_44
Parent CACHE3HI_1690_63
distance 0.09639

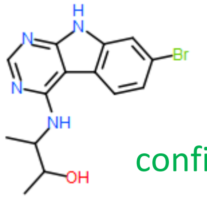

confirmed

CACHE_ID CACHE3-HO_1690_37
Parent CACHE3HI_1690_63
distance 0.1008

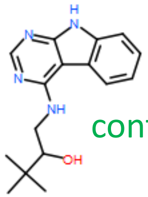

confirmed

CACHE_ID CACHE3-HO_1690_40
Parent CACHE3HI_1690_63
distance 0.1255

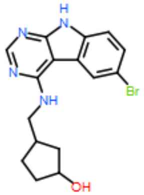

CACHE_ID CACHE3-HO_1690_38
Parent CACHE3HI_1690_63
distance 0.1269

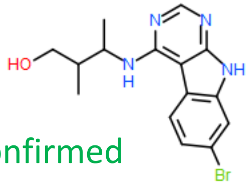

confirmed

CACHE_ID CACHE3-HO_1690_41
Parent CACHE3HI_1690_63
distance 0.149

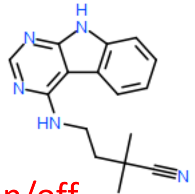

slow on/off

CACHE_ID CACHE3-HO_1690_33
Parent CACHE3HI_1690_63
distance 0.1746

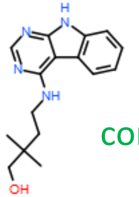

confirmed

CACHE_ID CACHE3-HO_1690_42
Parent CACHE3HI_1690_63
distance 0.1753

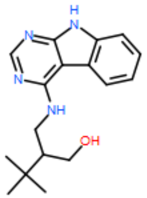

CACHE_ID CACHE3-HO_1690_39
Parent CACHE3HI_1690_63
distance 0.1811

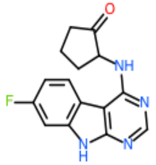

Closest
published hit
dist: 0.2
Ki:12 µM

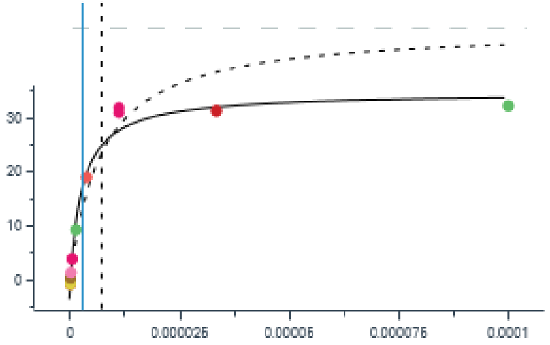

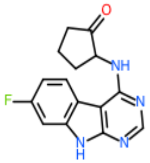

Closest
published hit
dist: 0.2
Ki:12 µM

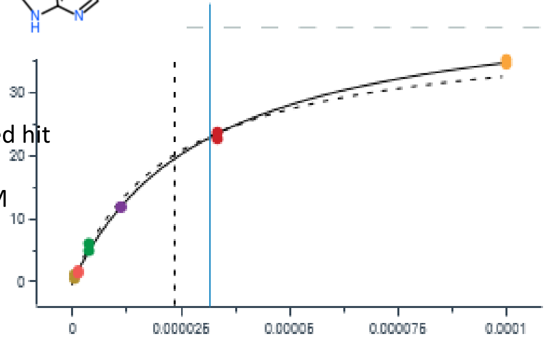

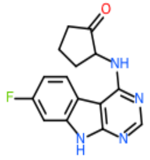

Closest
published hit
dist: 0.2
Ki:12 µM

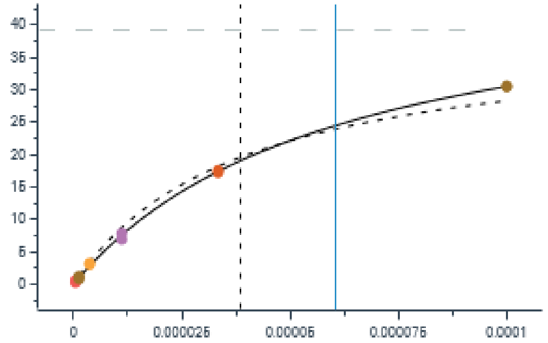

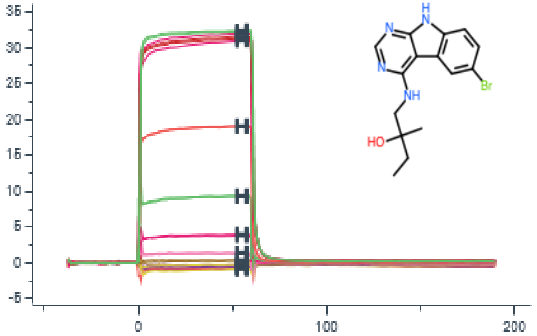

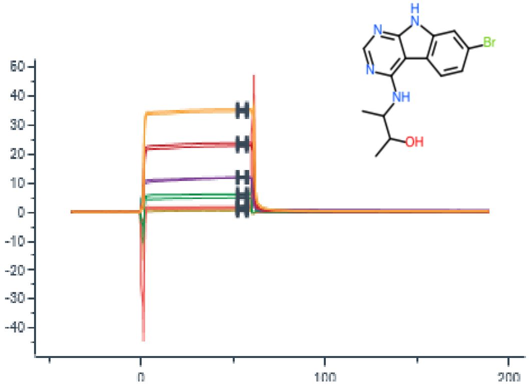

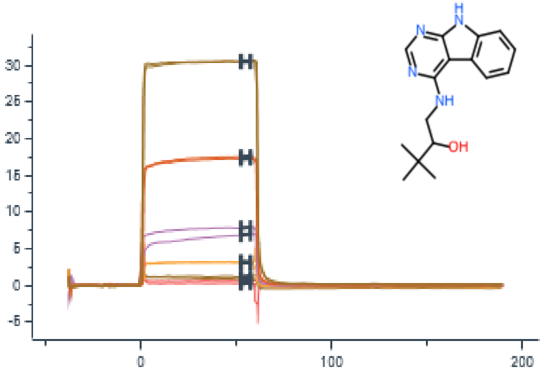

CACHE3-HO_1690_43

K_D = 3 µM – 75% binding
Selectivity for NSP3 (against PARP14a protein) – Yes
DLS (solub@100 µM)
HTRF_displacement hit confirmation (4% DMSO):
%inh@100 uM = 83
%inh@30 uM = 44
%inh@10 uM = 28

CACHE3-HO_1690_37

K_D (run 1) = 31 µM – 114% binding
K_D (run 2) = 42 µM – 137% binding
Selectivity for NSP3 (against PARP14a protein) – Yes
DLS (solub@100 µM)
HTRF_displacement hit confirmation (4% DMSO):
%inh@100 uM = 53
%inh@30 uM = 23
%inh@10 uM = 12

CACHE3-HO_1690_40

K_D (run 1) = 60 µM – 125% binding
K_D (run 2) = 51 µM – 96% binding
Selectivity for NSP3 (against PARP14a protein) – Yes
DLS (solub@100 µM)
HTRF_displacement hit confirmation (4% DMSO):
%inh@100 uM = 41
%inh@30 uM = 15
%inh@10 uM = 9

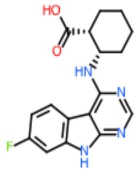

Closest published hit
dist: 0.2
Ki:1.5 µM

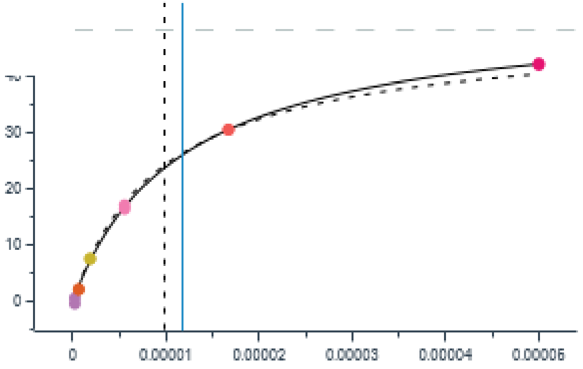

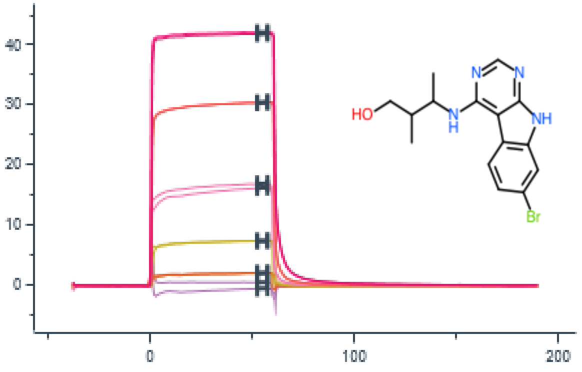

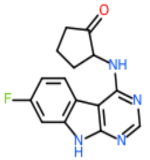

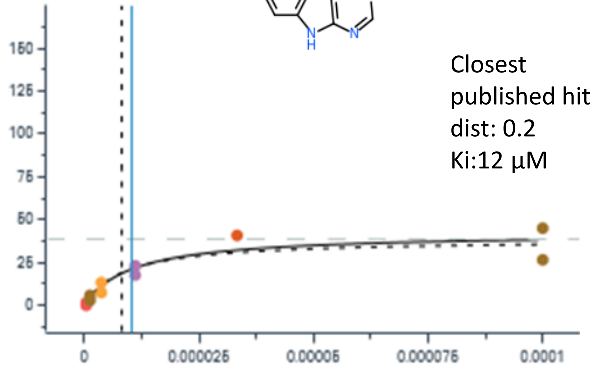

Closest published hit
dist: 0.2
Ki:12 µM

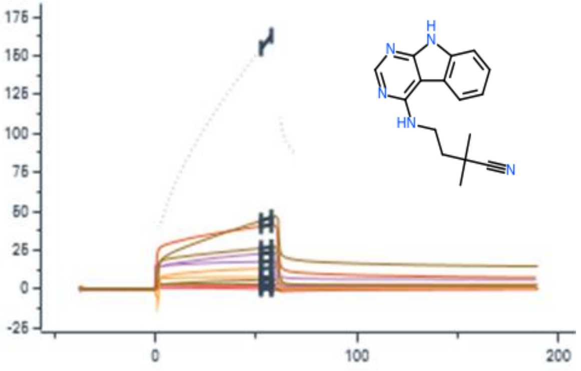

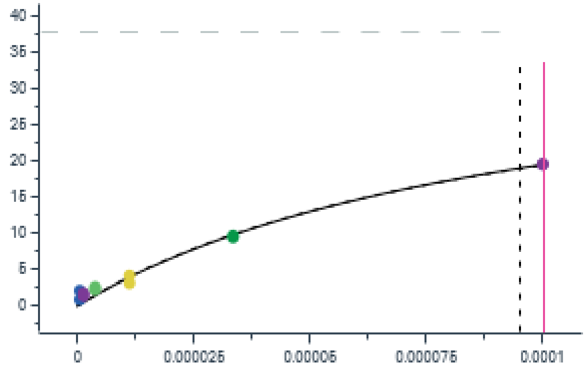

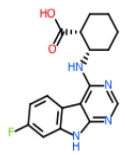

Closest published hit
dist: 0.2
Ki:1.5 µM

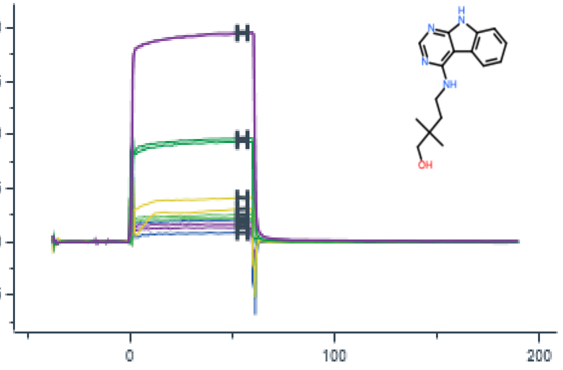

CACHE3-HO_1690_41

K_D (run 1) = 7 µM –93% binding
K_D (run 2) = 12 µM –108% binding
Selectivity for NSP3 (against PARP14a protein) – Yes
DLS (solub@50 µM)
HTRF_displacement hit confirmation (4% DMSO):
%inh@100 uM = 72
%inh@30 uM = 43
%inh@10 uM = 27

CACHE3-HO_1690_33

K_D = 10 µM (slow on/off) – 109% binding
Selectivity for NSP3 (against PARP14a protein) – Yes
DLS (solub@100 µM)
HTRF_displacement hit confirmation (4% DMSO):
%inh@100 uM = 46
%inh@30 uM = 28
%inh@10 uM = 19

CACHE3-HO_1690_42

K_D (run 1) = 38 µM – 57% binding
K_D (run 2) = 100 µM – 103% binding
Selectivity for NSP3 (against PARP14a protein) – Yes
DLS (solub@100 µM)
HTRF_displacement hit confirmation (4% DMSO):
%inh@100 uM = 31
%inh@30 uM = 17
%inh@10 uM = 17

HIT MOLECULE that was not followed up for round 2

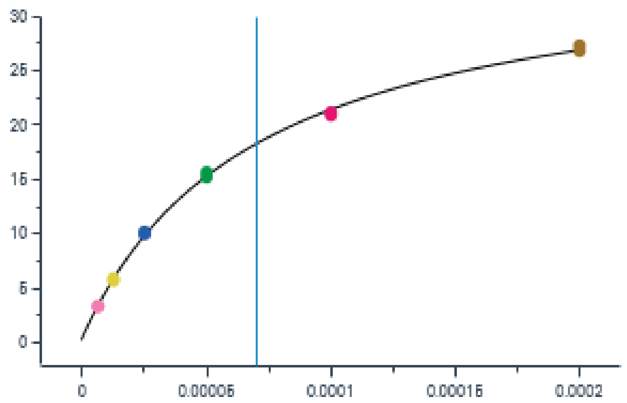

Closest
published hit
dist: 0.3
Ki:177 µM

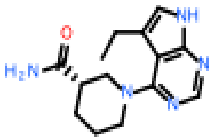

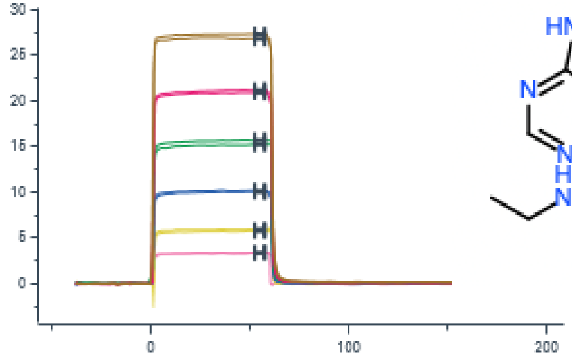

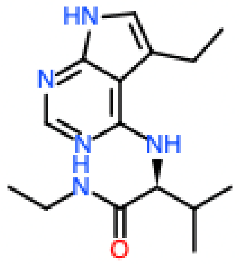

CACHE3HI_1690_87

K_D (run 1) = 70 µM – 105% binding
K_D (run 2) = 103 µM – 109% binding
Selectivity for NSP3 (against PARP14a protein) – mild binding
DLS (solub@100 µM)
HTRF_displacement:
%inh@100 uM = 48
%inh@50 uM = 36
%inh@25 uM = 20

PARENT MOLECULE

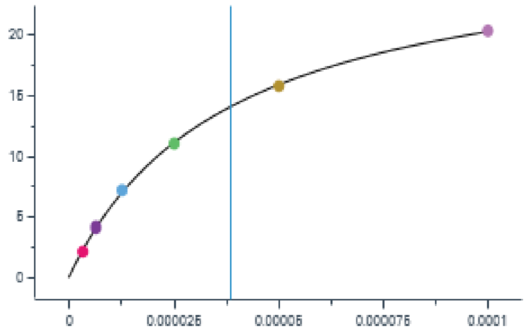

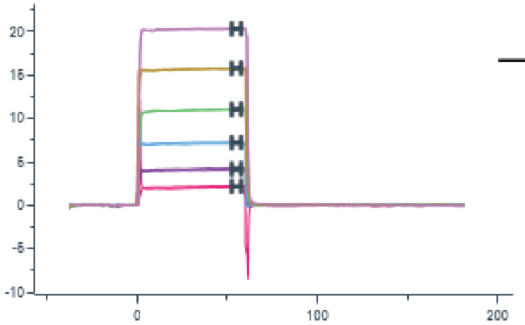

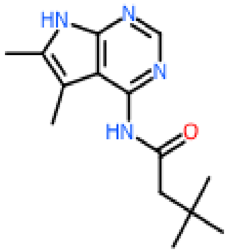

CACHE3HI_1690_92

K_D = 38 µM – 116% binding

Selectivity for NSP3 (against PARP14a protein) – Yes

DLS (solub@100 µM)

HTRF_displacement:

%inh@100 uM = 67

%inh@50 uM = 50

%inh@25 uM = 29

Structure – Yes !

6 analogs, including a parent molecule, of CACHE3HI_1690_92 chemotype were submitted for round 2.

4 compounds, including a re-supplied parent molecule showed a dose depended displacement of ADP-ribose peptide by HTRF. **3 compounds** confirmed dose dependent binding response by SPR with a tendency to reach saturation (1 compound showed stickiness)

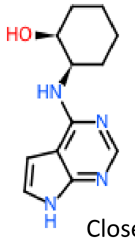

Closest published hit
dist: 0.3
Ki:114 µM

CACHE3-HO_1690_48

K_D = 92 µM – 97% binding

DLS (solub@100 µM)

HTRF_displacement hit confirmation (4% DMSO):

%inh@100 uM = 36

%inh@30 uM = 6

%inh@10 uM = 7

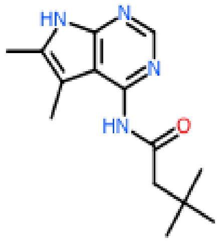

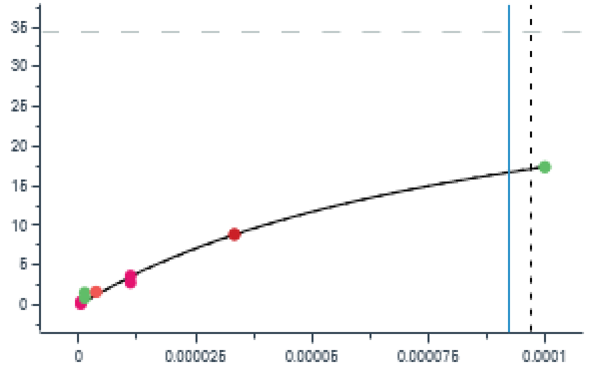

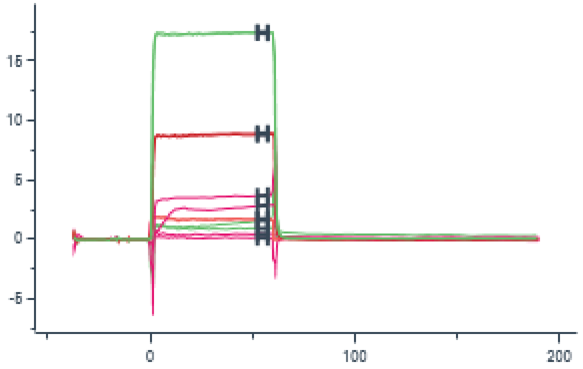

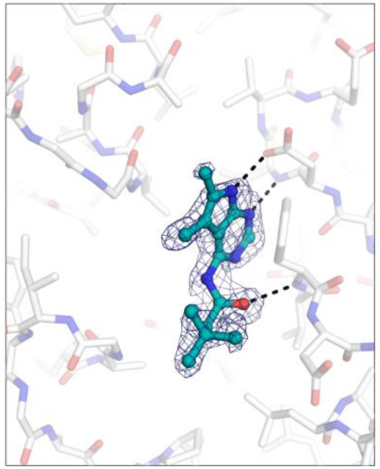

Blue mesh: PanDDA event map contoured at 2 σ

Tested analogs

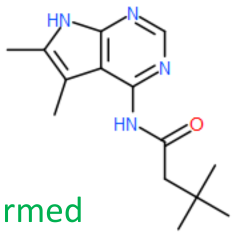 confirmed<br>CACHE_ID CACHE3-HO_1690_48<br>Parent CACHE3HI_1690_92<br>distance 0	slow on/off 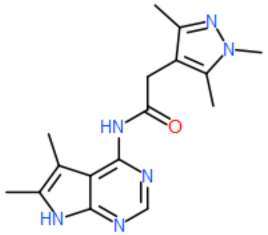<br>CACHE_ID CACHE3-HO_1690_34<br>Parent CACHE3HI_1690_92<br>distance 0.2218	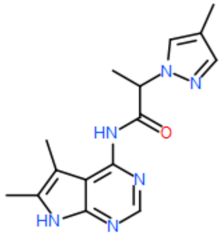<br>CACHE_ID CACHE3-HO_1690_24<br>Parent CACHE3HI_1690_92<br>distance 0.2677	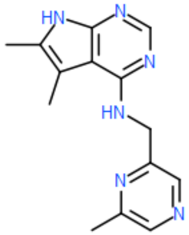<br>CACHE_ID CACHE3-HO_1690_9<br>Parent CACHE3HI_1690_92<br>distance 0.272
confirmed 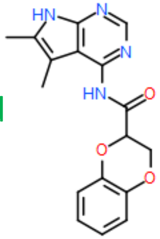<br>CACHE_ID CACHE3-HO_1690_3<br>Parent CACHE3HI_1690_92<br>distance 0.3311	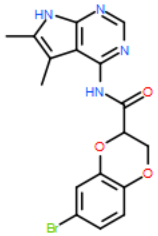<br>CACHE_ID CACHE3-HO_1690_1<br>Parent CACHE3HI_1690_92<br>distance 0.3657		

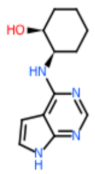

Closest
published hit
dist: 0.4
Ki:114 µM

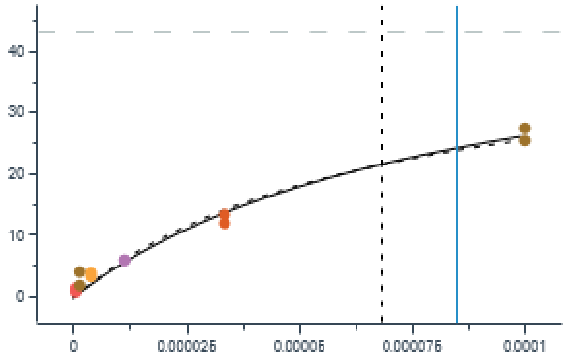

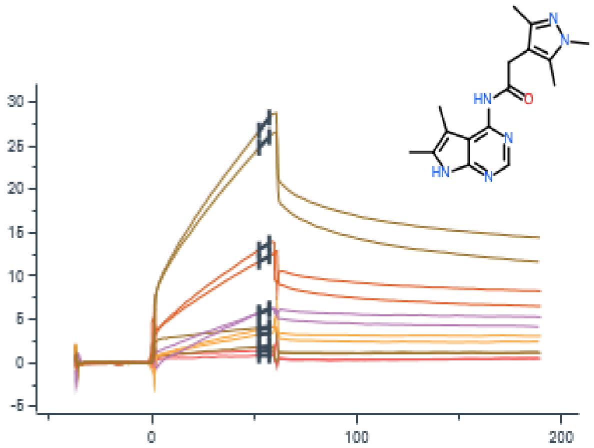

CACHE3-HO_1690_34

K_D = 85µM (slow on/off) – 112% binding
Selectivity for NSP3 (against PARP14a protein) – 147% binding
DLS (solub@100 µM)
HTRF_displacement hit confirmation (4% DMSO):
%inh@100 uM = 73
%inh@30 uM = 60
%inh@10 uM = 38

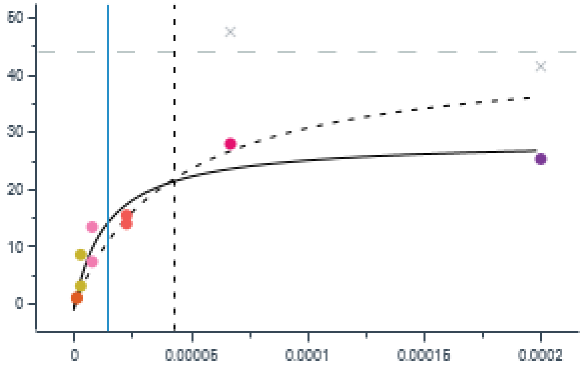

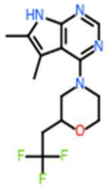

Closest
published hit
dist: 0.4
Ki:119 µM

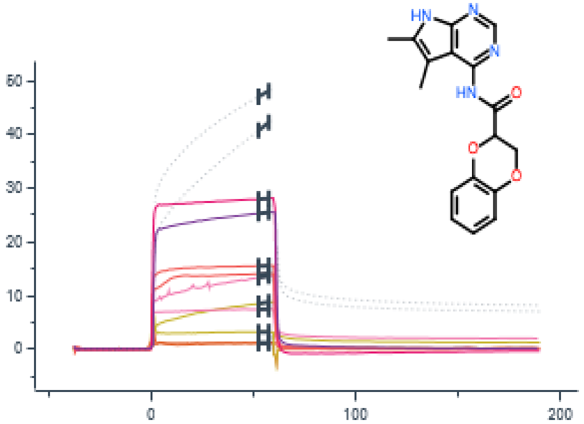

CACHE3-HO_1690_3

K_D (run 1) = 121 µM (poor fit) – 137% binding
K_D (run 2) = 14 µM (poor fit) – 65% binding
Selectivity for NSP3 (against PARP14a protein) – 25% binding
DLS (solub@100 µM)
HTRF_displacement hit confirmation (4% DMSO):
%inh@100 uM = 44
%inh@30 uM = 9
%inh@10 uM = 10

CACHE#3 – SARS-CoV2 Nsp3 macrodomain
Participant 1696

PARENT MOLECULE

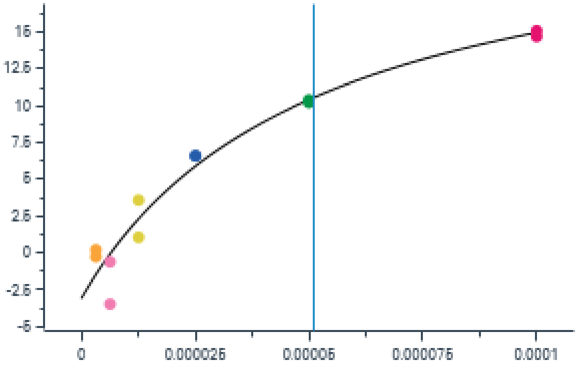

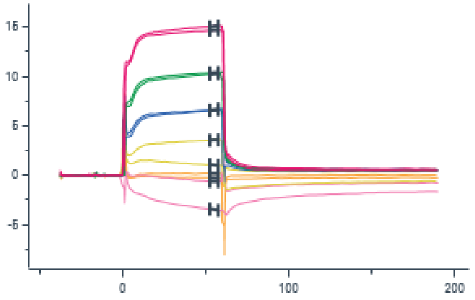

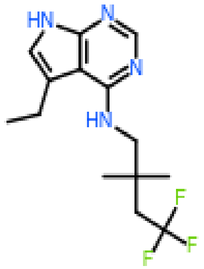

CACHE3HI_1696_6

K_D = 51 µM – 73% binding

Selectivity for NSP3 (against PARP14a protein) – Yes

DLS (solub@100 µM)

HTRF_displacement:

%inh@100 uM = 45

%inh@50 uM = 25

%inh@25 uM = 10

Structure – Yes !

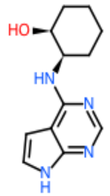

Closest published hit
dist: 0.3
Ki:114 µM

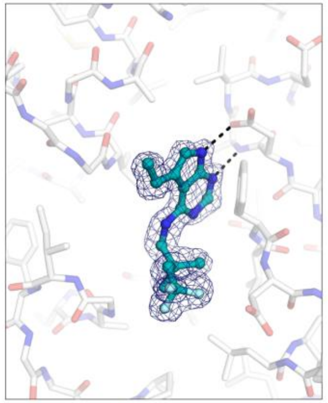

Blue mesh: PanDDA event map contoured at 2 σ

14 analogs, including a parent molecule, of CACHE3HI_1696_6 chemotype were submitted for round 2.
6 compounds, including a re-supplied parent molecule showed a dose depended displacement of ADP-ribose peptide by HTRF. **3 compounds** showed a dose dependent binding response, however, 2 of them showed a tendency to reach saturation

CACHE3-HO_1696_45

K_D = 17 µM – 88% binding

DLS (solub@30 µM)

HTRF_displacement hit confirmation (4% DMSO):

%inh@100 uM = 38

%inh@30 uM = 16

%inh@10 uM = 14

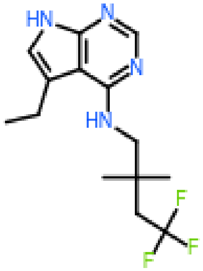

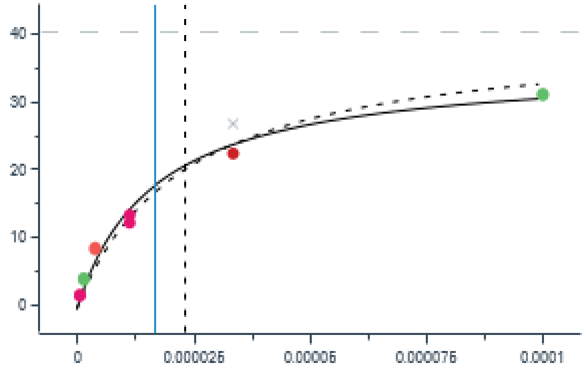

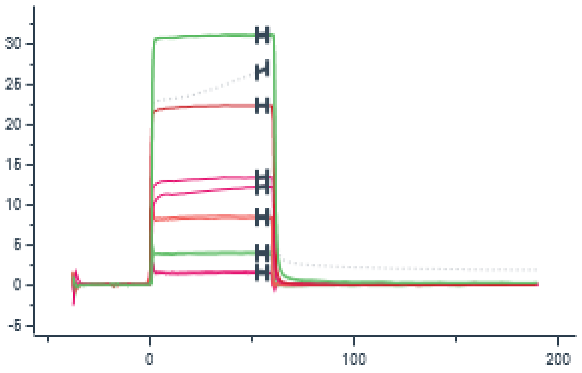

Tested analogs

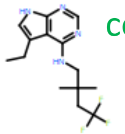

confirmed

CACHE_ID CACHE3-HO_1696_45
Parent CACHE3HI_1696_6
distance 0

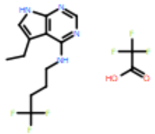

CACHE_ID CACHE3-HO_1696_7
Parent CACHE3HI_1696_6
distance 0.02525

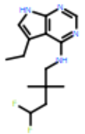

confirmed

CACHE_ID CACHE3-HO_1696_11
Parent CACHE3HI_1696_6
distance 0.0398

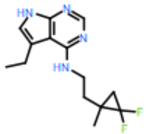

CACHE_ID CACHE3-HO_1696_3
Parent CACHE3HI_1696_6
distance 0.07109

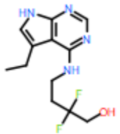

CACHE_ID CACHE3-HO_1696_5
Parent CACHE3HI_1696_6
distance 0.1

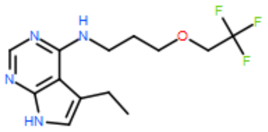

CACHE_ID CACHE3-HO_1696_9
Parent CACHE3HI_1696_6
distance 0.1349

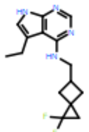

CACHE_ID CACHE3-HO_1696_12
Parent CACHE3HI_1696_6
distance 0.1511

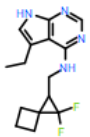

confirmed

CACHE_ID CACHE3-HO_1696_4
Parent CACHE3HI_1696_6
distance 0.1941

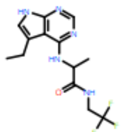

CACHE_ID CACHE3-HO_1696_1
Parent CACHE3HI_1696_6
distance 0.2213

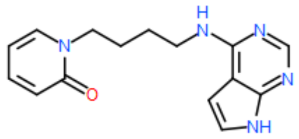

CACHE_ID CACHE3-HO_1696_39
Parent CACHE3HI_1696_6
distance 0.3444

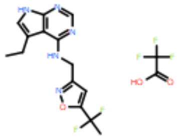

CACHE_ID CACHE3-HO_1696_6
Parent CACHE3HI_1696_6
distance 0.3521

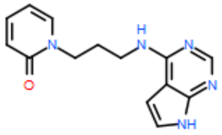

CACHE_ID CACHE3-HO_1696_41
Parent CACHE3HI_1696_6
distance 0.3663

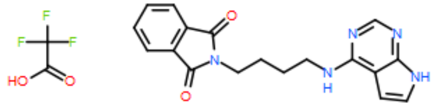

CACHE_ID CACHE3-HO_1696_37
Parent CACHE3HI_1696_6
distance 0.3855

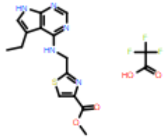

CACHE_ID CACHE3-HO_1696_8
Parent CACHE3HI_1696_6
distance 0.4161

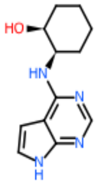

Closest published hit dist: 0.3 Ki:114 µM

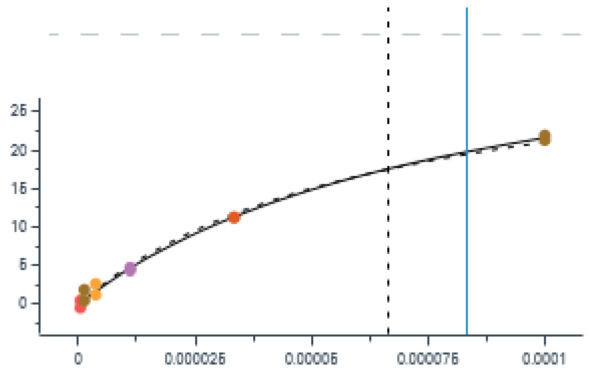

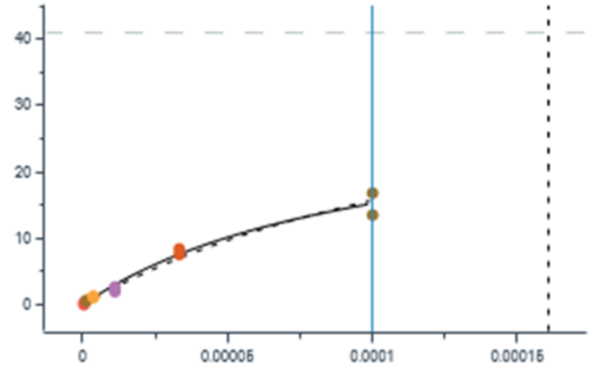

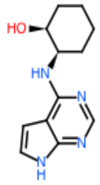

Closest published hit dist: 0.37 Ki:114 µM

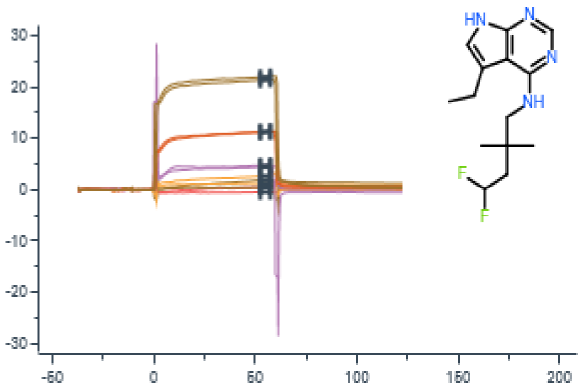

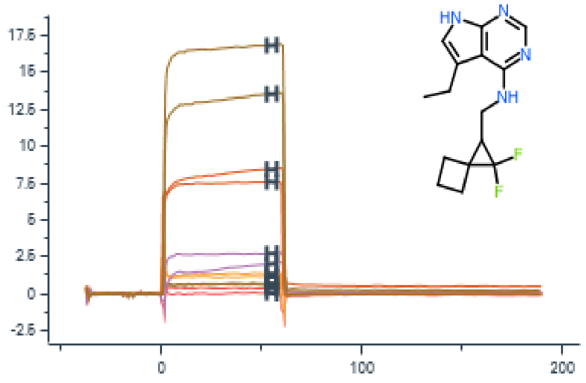

CACHE3-HO_1696_11

K_D (run 1) = 86 µM (predicted) – 94% binding
K_D (run 2) = 83 µM – 113% binding
Selectivity for NSP3 (against PARP14a protein) – 106% binding
DLS (solub@50 µM)
HTRF_displacement hit confirmation (4% DMSO):
%inh@100 uM = 51
%inh@30 uM = 21
%inh@10 uM = 8

CACHE3-HO_1696_4

K_D = 100 µM – 74% binding
Selectivity for NSP3 (against PARP14a protein) – linear response – 267% binding
DLS (solub@30 µM)
HTRF_displacement hit confirmation (4% DMSO):
%inh@100 uM = 30
%inh@30 uM = 37
%inh@10 uM = 16

PARENT MOLECULE

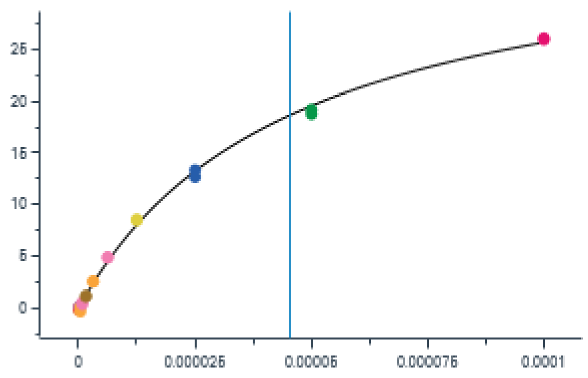

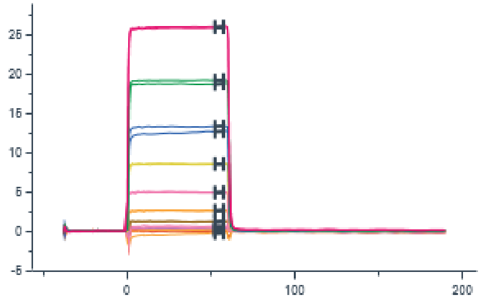

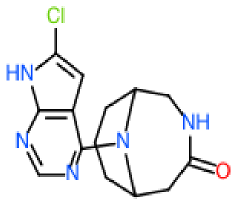

CACHE3HI_1696_50

K_D = 45 µM – 104% binding
Selectivity for NSP3 (against PARP14a protein) – Yes
DLS (solub@100 µM)
HTRF_displacement:
%inh@100 uM = 57
%inh@50 uM = 40
%inh@25 uM = 26

Structure – Yes !

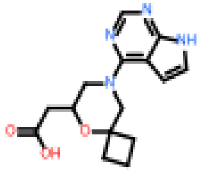

Closest published hit
dist: 0.3
Ki:114 µM

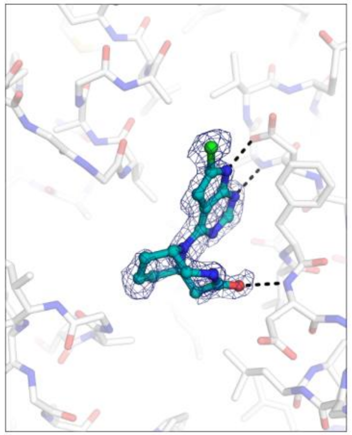

Blue mesh: PanDDA event map contoured at 2 σ

11 analogs, including a parent molecule, of CACHE3HI_1696_50 chemotype were submitted for round 2.
6 compounds, including a re-supplied parent molecule showed a dose depended displacement of ADP-ribose peptide by HTRF. **2 compounds** confirmed binding affinity by SPR.

CACHE3-HO_1696_46

K_D = 87 µM –137% binding
DLS (solub@100 µM)
HTRF_displacement hit confirmation (4% DMSO):
%inh@100 uM = 43
%inh@30 uM = 15
%inh@10 uM = 5

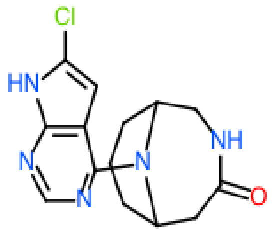

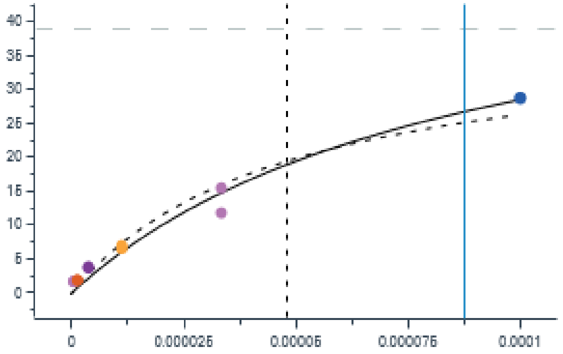

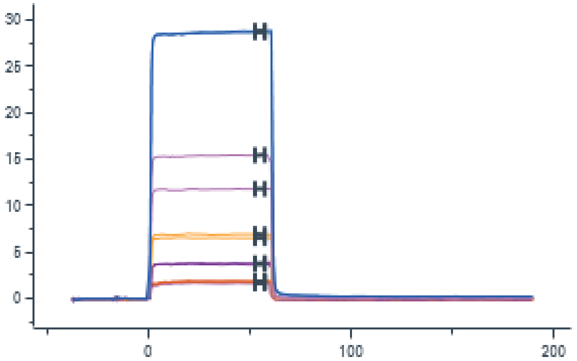

Tested analogs

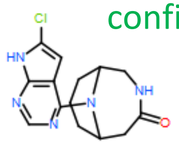

confirmed

CACHE_ID CACHE3-HO_1696_46
Parent CACHE3HI_1696_50
distance 0

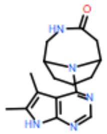
confirmed

CACHE_ID CACHE3-HO_1696_18
Parent CACHE3HI_1696_50
distance 0.1409

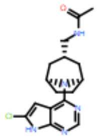

CACHE_ID CACHE3-HO_1696_23
Parent CACHE3HI_1696_50
distance 0.2483

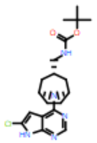

CACHE_ID CACHE3-HO_1696_13
Parent CACHE3HI_1696_50
distance 0.3103

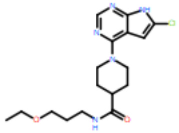

CACHE_ID CACHE3-HO_1696_21
Parent CACHE3HI_1696_50
distance 0.355

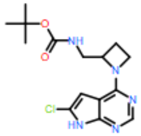

CACHE_ID CACHE3-HO_1696_20
Parent CACHE3HI_1696_50
distance 0.3703

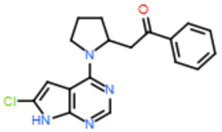

CACHE_ID CACHE3-HO_1696_17
Parent CACHE3HI_1696_50
distance 0.3871

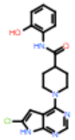

CACHE_ID CACHE3-HO_1696_15
Parent CACHE3HI_1696_50
distance 0.3944

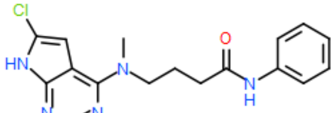

CACHE_ID CACHE3-HO_1696_16
Parent CACHE3HI_1696_50
distance 0.402

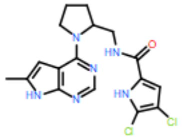

CACHE_ID CACHE3-HO_1696_14
Parent CACHE3HI_1696_50
distance 0.4419

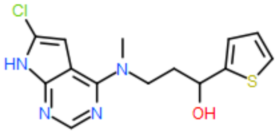

CACHE_ID CACHE3-HO_1696_19
Parent CACHE3HI_1696_50
distance 0.4941

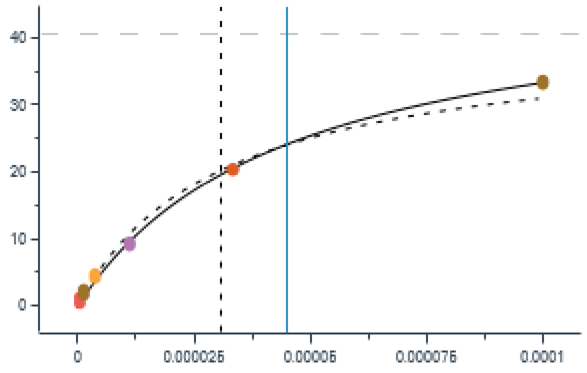

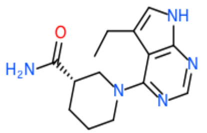

Closest
published hit
dist: 0.4
Ki:177 µM

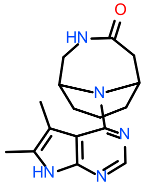

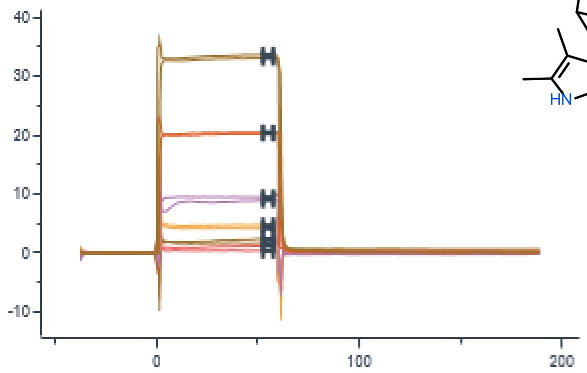

CACHE3-HO_1696_18

K_D (run 1) = 44 µM – 121% binding
K_D (run 2) = 45 µM – 119% binding
Selectivity for NSP3 (against PARP14a protein) – Yes
DLS (solub@100 µM)
HTRF_displacement hit confirmation (4% DMSO):
%inh@100 uM = 60
%inh@30 uM = 25
%inh@10 uM = 7

PARENT MOLECULE

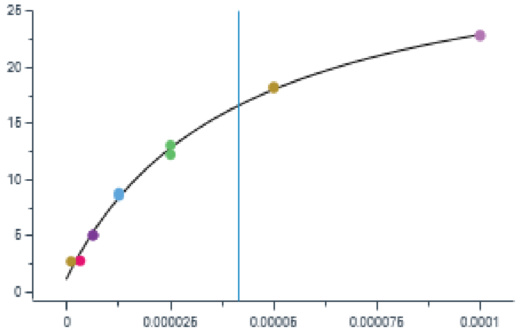

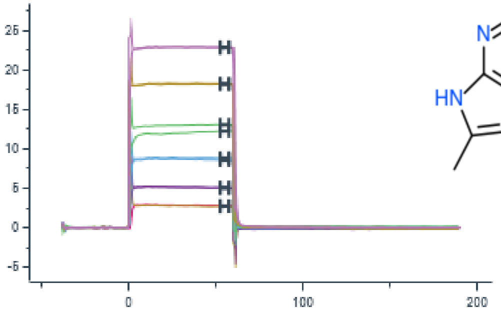

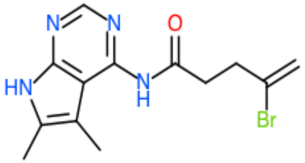

CACHE3HI_1696_67

K_D = 42 µM – 103% binding

Selectivity for NSP3 (against PARP14a protein) – Yes

DLS (solub@100 µM)

HTRF_displacement:

%inh@100 uM = 56

%inh@50 uM = 40

%inh@25 uM = 26

Structure – Yes !

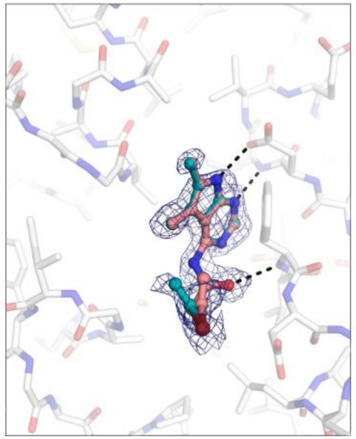

Blue mesh: PanDDA event map contoured at 2 σ

7 analogs, including a parent molecule, of CACHE3HI_1696_67 chemotype were submitted for round 2.
5 compounds, including a re-supplied parent molecule showed a dose depended displacement of ADP-ribose peptide by HTRF. **2 compounds** showed dose dependent binding response by SPR

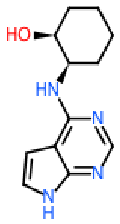

Closest published hit
dist: 0.4
Ki:114 µM

CACHE3-HO_1696_47

K_D = 64 µM – 111% binding

DLS (solub@30 µM)

HTRF_displacement hit confirmation (4% DMSO):

%inh@100 uM = 52

%inh@30 uM = 17

%inh@10 uM = 11

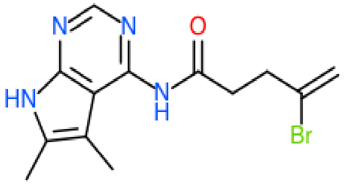

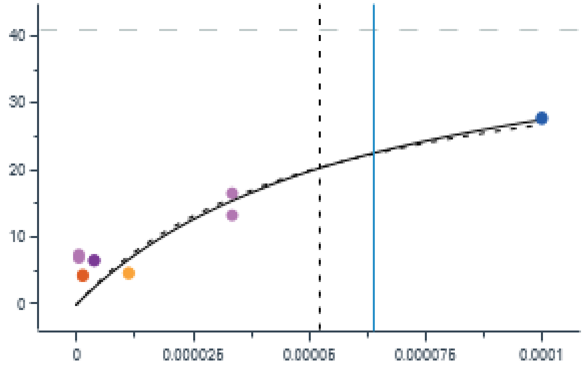

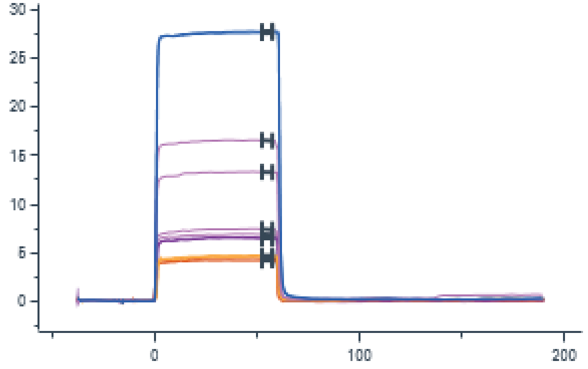

Tested analogs

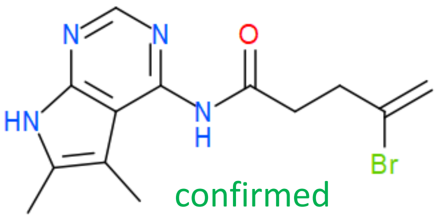

CACHE_ID CACHE3-HO_1696_47
Parent CACHE3HI_1696_67
distance 0

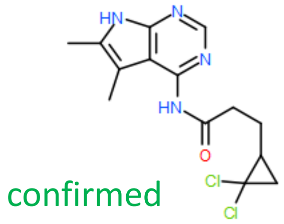

CACHE_ID CACHE3-HO_1696_30
Parent CACHE3HI_1696_67
distance 0.1475

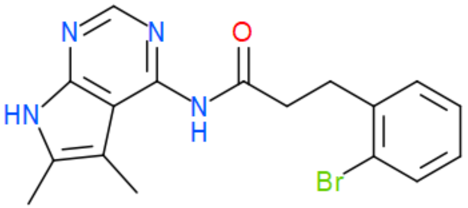

CACHE_ID CACHE3-HO_1696_27
Parent CACHE3HI_1696_67
distance 0.1538

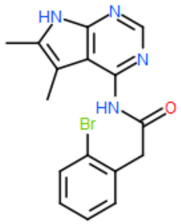

CACHE_ID CACHE3-HO_1696_29
Parent CACHE3HI_1696_67
distance 0.1579

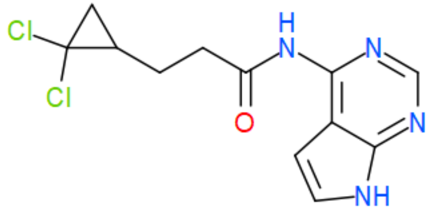

CACHE_ID CACHE3-HO_1696_32
Parent CACHE3HI_1696_67
distance 0.2623

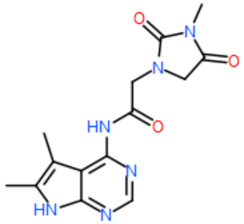

CACHE_ID CACHE3-HO_1696_34
Parent CACHE3HI_1696_67
distance 0.3102

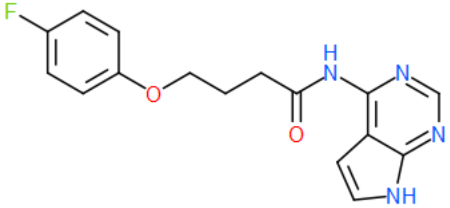

CACHE_ID CACHE3-HO_1696_31
Parent CACHE3HI_1696_67
distance 0.3419

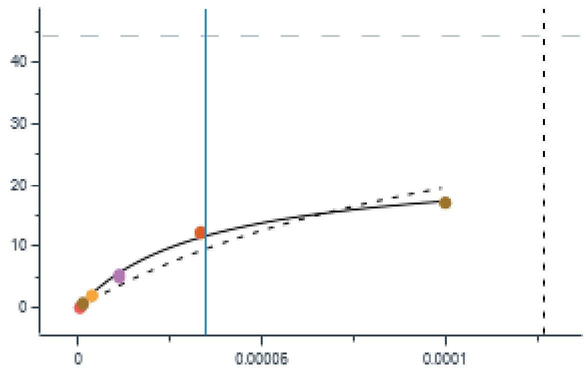

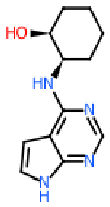

Closest published hit
dist: 0.3
Ki:114 µM

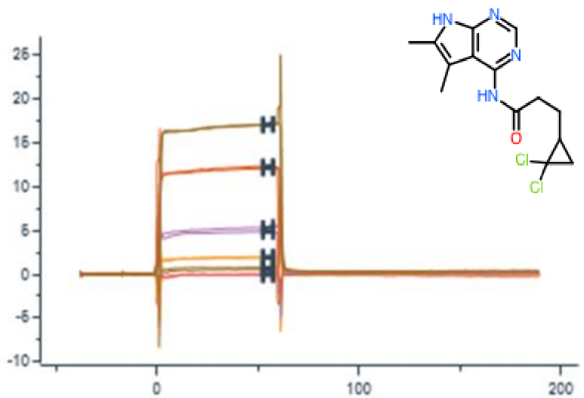

CACHE3-HO_1696_30

K_D (run 1) = 44 µM (predicted) – 49% binding
K_D (run 2) = 35 µM – 53% binding
Selectivity for NSP3 (against PARP14a protein) – 22% binding (slow off)
DLS (solub@50 µM)
HTRF_displacement hit confirmation (4% DMSO):
%inh@100 uM = 35
%inh@30 uM = 16
%inh@10 uM = 2

6 analogs, including a parent molecule, of CACHE3HI_1696_78 chemotype were submitted for round 2.
2 compounds, including a re-supplied parent molecule showed a dose depended displacement of ADP-ribose peptide by HTRF and confirmed binding affinity by SPR.

PARENT MOLECULE

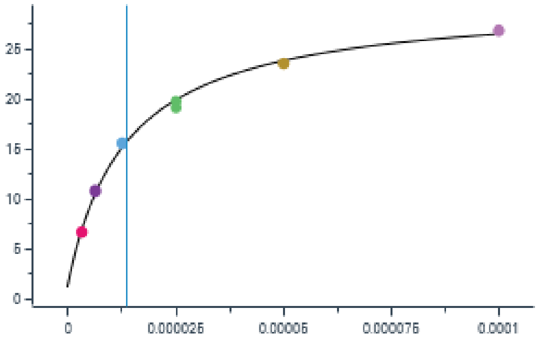

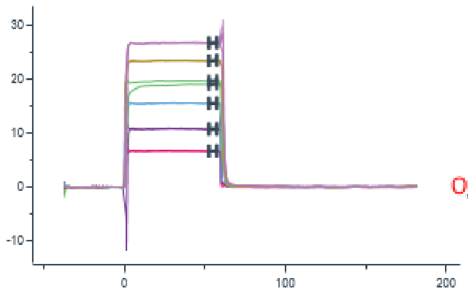

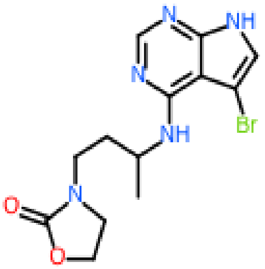

CACHE3HI_1696_78

K_D = 14 µM – 83% binding
Selectivity for NSP3 (against PARP14a protein) – Yes
DLS (solub@100 µM)
HTRF_displacement:
%inh@100 uM = 78
%inh@50 uM = 66
%inh@25 uM = 48

Structure – Yes !

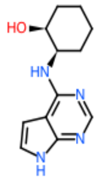

Closest published hit
dist: 0.4
Ki:114 µM

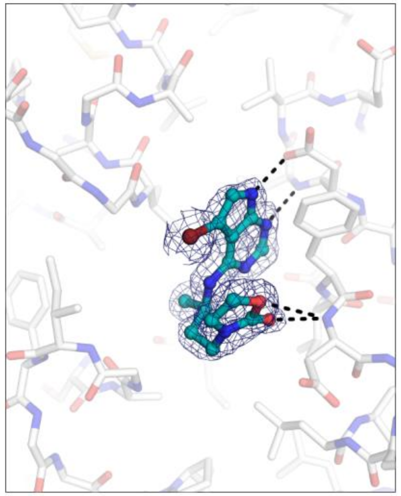

Blue mesh: PanDDA event map contoured at 2 σ

CACHE3-HO_1696_48

K_D = 17 µM – 73% binding
DLS (solub@100 µM)
HTRF_displacement hit confirmation (4% DMSO):
%inh@100 uM = 54
%inh@30 uM = 21
%inh@10 uM = 8

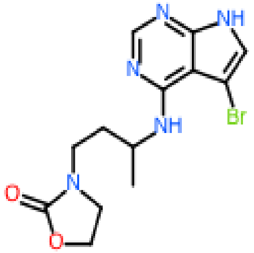

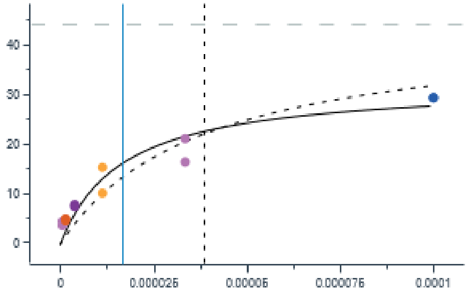

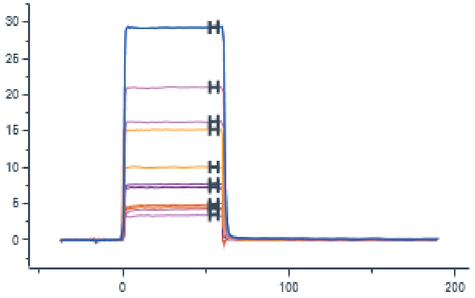

Tested analogs

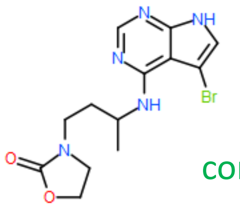

confirmed

CACHE_ID	CACHE3-HO_1696_48
Parent	CACHE3HI_1696_78
distance	0

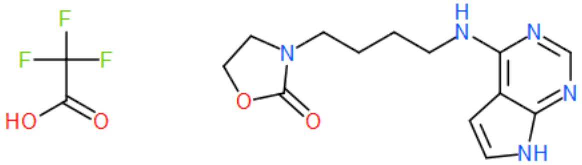

CACHE_ID	CACHE3-HO_1696_44
Parent	CACHE3HI_1696_78
distance	0.1169

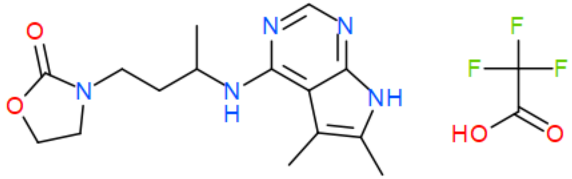

CACHE_ID	CACHE3-HO_1696_38
Parent	CACHE3HI_1696_78
distance	0.1624

confirmed

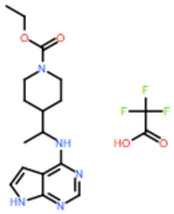

CACHE_ID	CACHE3-HO_1696_36
Parent	CACHE3HI_1696_78
distance	0.2261

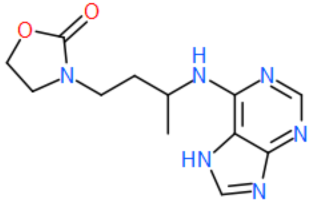

CACHE_ID	CACHE3-HO_1696_42
Parent	CACHE3HI_1696_78
distance	0.2741

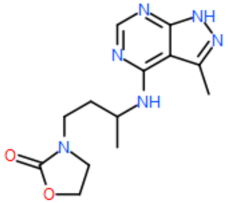

CACHE_ID	CACHE3-HO_1696_43
Parent	CACHE3HI_1696_78
distance	0.2972

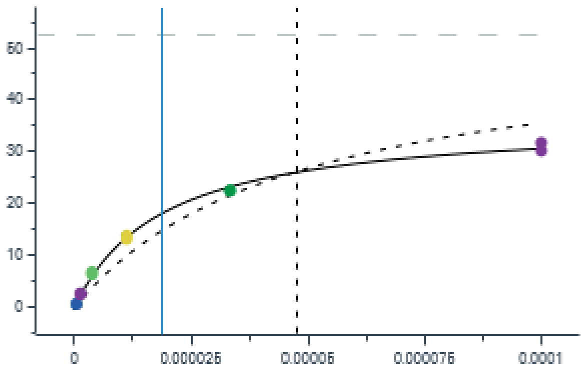

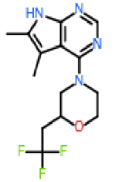

Closest published hit
dist: 0.3
Ki:118 µM

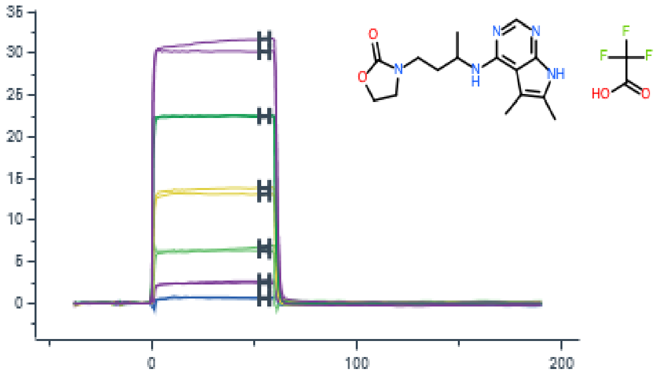

CACHE3-HO_1696_38

K_D (run 1) = 11µM – 60% binding
K_D (run 2) = 19µM – 69% binding
Selectivity for NSP3 (against PARP14a protein) – Yes
DLS (solub@50 µM)
HTRF_displacement hit confirmation (4% DMSO):
%inh@100 uM = 50
%inh@30 uM = 21
%inh@10 uM = 8

CACHE#3 – SARS-CoV2 Nsp3 macrodomain
Participant 1700

PARENT MOLECULE

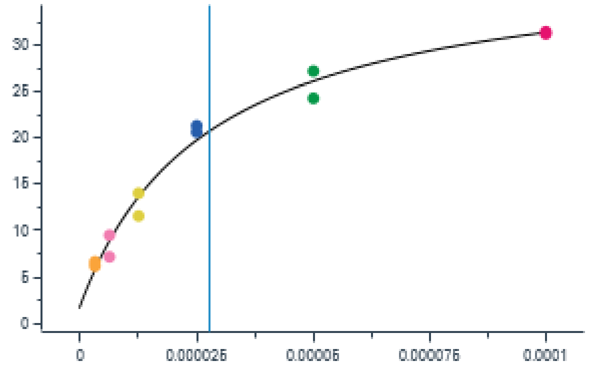

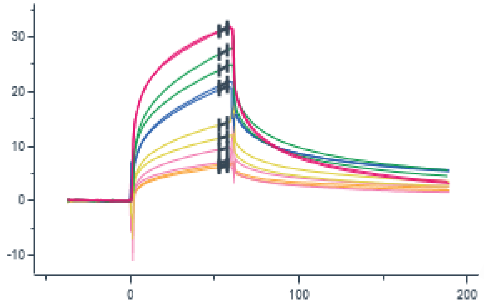

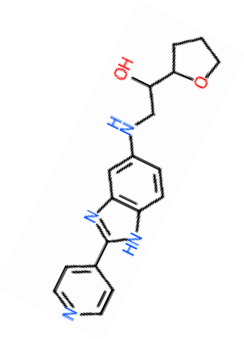

CACHE3HI_1700_52

K_D = 28 µM (slow on/off) – 94% binding

Selectivity for NSP3 (against PARP14a protein) – Yes

DLS (solub@100 µM)

HTRF_displacement:

%inh@100 uM = 81

%inh@50 uM = 69

%inh@25 uM = 32

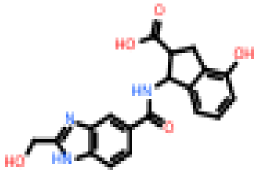

Closest published hit
dist: 0.5
Ki:1.1 µM

31 analogs, including a parent molecule, of CACHE3HI_1700_52 chemotype were submitted for round 2.

9 compounds, including a re-supplied parent molecule showed a dose depended displacement of ADP-ribose peptide by HTRF and binding response by SPR. **4 compounds** showed a dose dependent binding response by SPR (re-supplied molecule showed a low binding response)

CACHE3-HO_1700_36

K_D = 19 µM (low binding/ slow on/off) –

23% binding

DLS (solub@100 µM)

HTRF_displacement hit confirmation (4% DMSO):

%inh@100 uM = 73

%inh@30 uM = 22

%inh@10 uM = 5

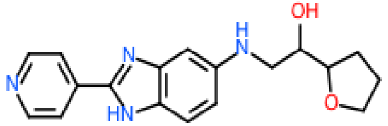

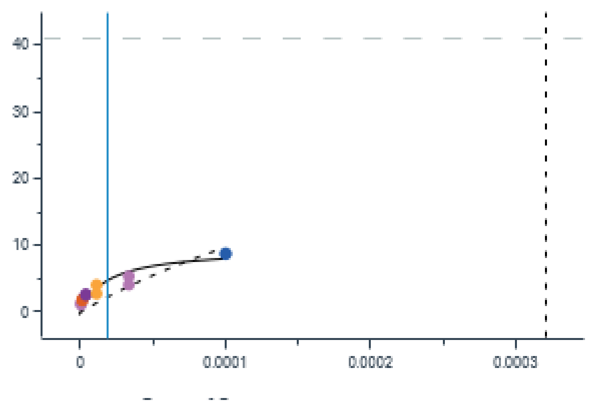

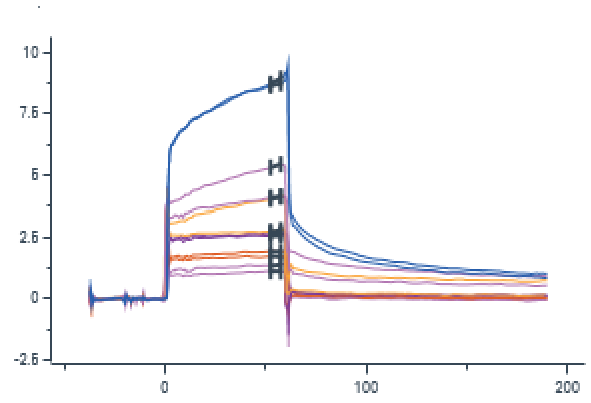

Tested analogs

confirmed

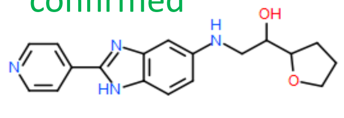

CACHE_ID CACHE3-HO_1700_36
Parent CACHE3HI_1700_52
distance 0

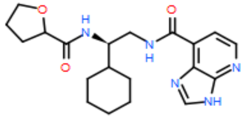

CACHE_ID CACHE3-HO_1700_28
Parent CACHE3HI_1700_52
distance 0.4178

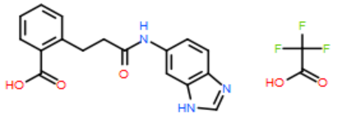

CACHE_ID CACHE3-HO_1700_14
Parent CACHE3HI_1700_52
distance 0.444

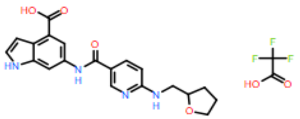

CACHE_ID CACHE3-HO_1700_32
Parent CACHE3HI_1700_52
distance 0.4462

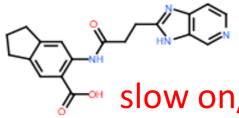

slow on/off

CACHE_ID CACHE3-HO_1700_3
Parent CACHE3HI_1700_52
distance 0.4742

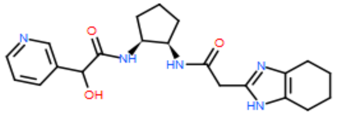

CACHE_ID CACHE3-HO_1700_29
Parent CACHE3HI_1700_52
distance 0.4809

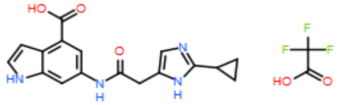

CACHE_ID CACHE3-HO_1700_23
Parent CACHE3HI_1700_52
distance 0.4844

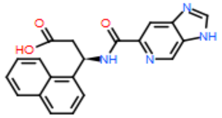

CACHE_ID CACHE3-HO_1700_11
Parent CACHE3HI_1700_52
distance 0.4921

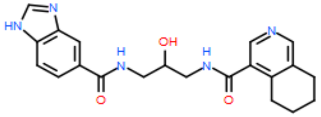

CACHE_ID CACHE3-HO_1700_31
Parent CACHE3HI_1700_52
distance 0.4952

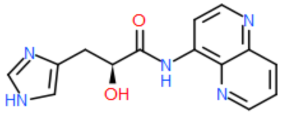

CACHE_ID CACHE3-HO_1700_30
Parent CACHE3HI_1700_52
distance 0.5097

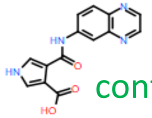

confirmed

CACHE_ID CACHE3-HO_1700_22
Parent CACHE3HI_1700_52
distance 0.5161

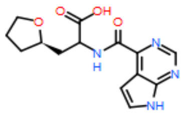

CACHE_ID CACHE3-HO_1700_34
Parent CACHE3HI_1700_52
distance 0.5174

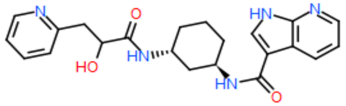

CACHE_ID CACHE3-HO_1700_35
Parent CACHE3HI_1700_52
distance 0.522

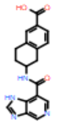

CACHE_ID CACHE3-HO_1700_21
Parent CACHE3HI_1700_52
distance 0.5368

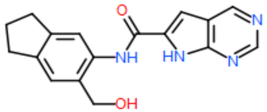

CACHE_ID CACHE3-HO_1700_33
Parent CACHE3HI_1700_52
distance 0.5668

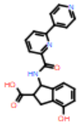

CACHE_ID CACHE3-HO_1700_27
Parent CACHE3HI_1700_52
distance 0.5888

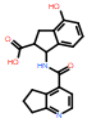

CACHE_ID CACHE3-HO_1700_18
Parent CACHE3HI_1700_52
distance 0.6017

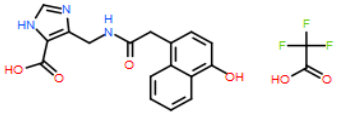

CACHE_ID CACHE3-HO_1700_10
Parent CACHE3HI_1700_52
distance 0.6106

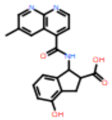

CACHE_ID CACHE3-HO_1700_9
Parent CACHE3HI_1700_52
distance 0.611

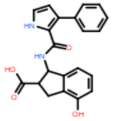

CACHE_ID CACHE3-HO_1700_7
Parent CACHE3HI_1700_52
distance 0.6116

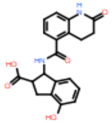

CACHE_ID CACHE3-HO_1700_8
Parent CACHE3HI_1700_52
distance 0.6117

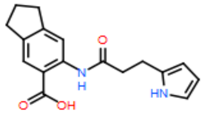

CACHE_ID CACHE3-HO_1700_17
Parent CACHE3HI_1700_52
distance 0.6129

confirmed

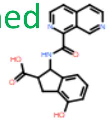

CACHE_ID CACHE3-HO_1700_2
Parent CACHE3HI_1700_52
distance 0.6188

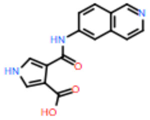

CACHE_ID CACHE3-HO_1700_26
Parent CACHE3HI_1700_52
distance 0.6224

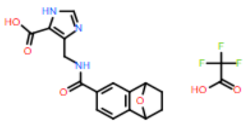

CACHE_ID CACHE3-HO_1700_25
Parent CACHE3HI_1700_52
distance 0.6236

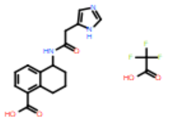

CACHE_ID CACHE3-HO_1700_1
Parent CACHE3HI_1700_52
distance 0.6341

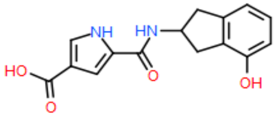

CACHE_ID CACHE3-HO_1700_19
Parent CACHE3HI_1700_52
distance 0.6344

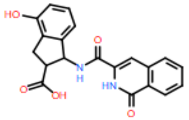

CACHE_ID CACHE3-HO_1700_20
Parent CACHE3HI_1700_52
distance 0.6382

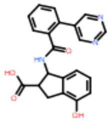

CACHE_ID CACHE3-HO_1700_4
Parent CACHE3HI_1700_52
distance 0.6483

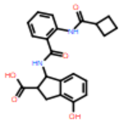

CACHE_ID CACHE3-HO_1700_6
Parent CACHE3HI_1700_52
distance 0.6628

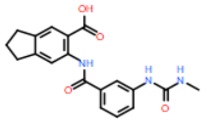

CACHE_ID CACHE3-HO_1700_24
Parent CACHE3HI_1700_52
distance 0.6656

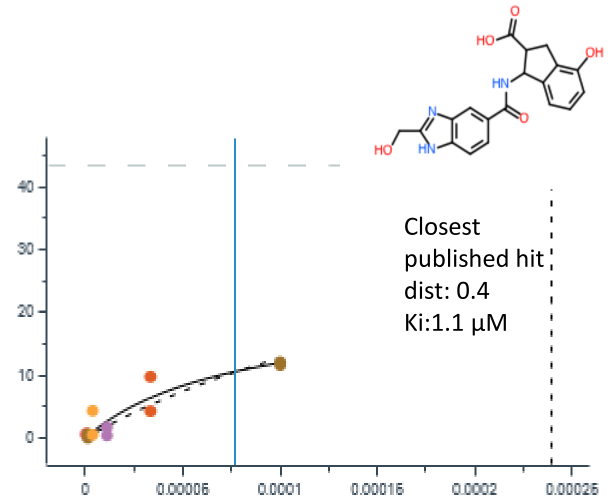

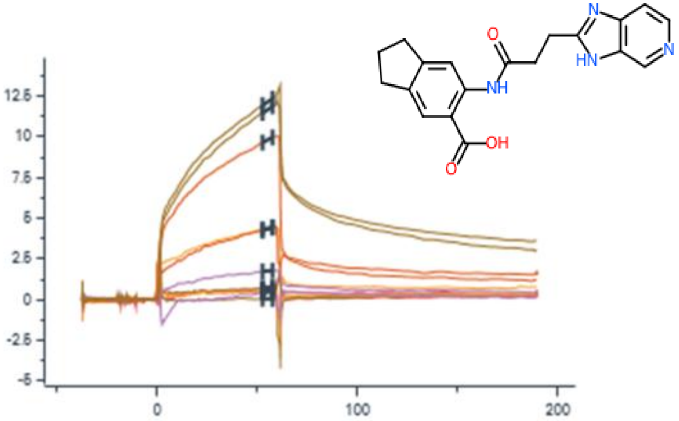

CACHE3-HO_1700_3

K_D = 76µM (slow on/off) – 49% binding
Selectivity for NSP3 (against PARP14a protein) – 26% binding
DLS (solub@100 µM)
HTRF_displacement hit confirmation (4% DMSO):
%inh@100 uM = 80
%inh@30 uM = 32
%inh@10 uM = 22

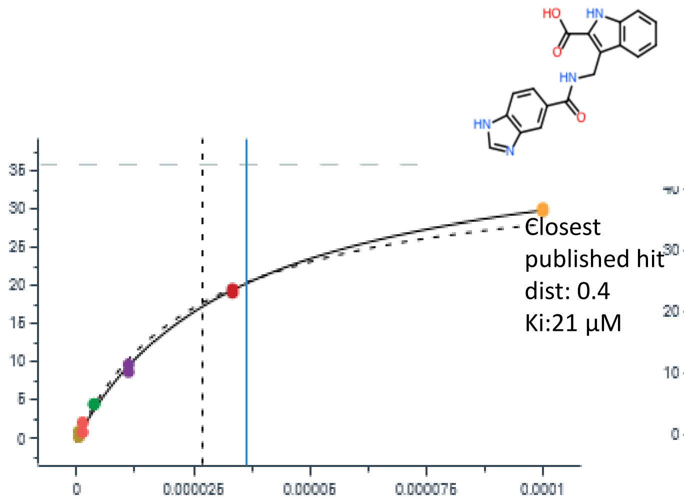

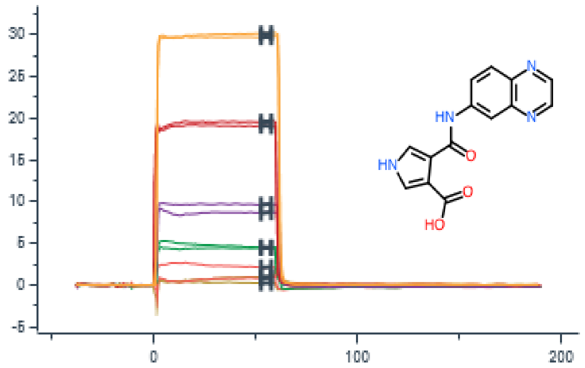

CACHE3-HO_1700_22

K_D (run 1) = 36 µM– 114% binding
K_D (run 2) = 21 µM (low binding) – 23% binding
Selectivity for NSP3 (against PARP14a protein) – Yes
DLS (solub@100 µM)
HTRF_displacement hit confirmation (4% DMSO):
%inh@100 uM = 55
%inh@30 uM = 18
%inh@10 uM = 7

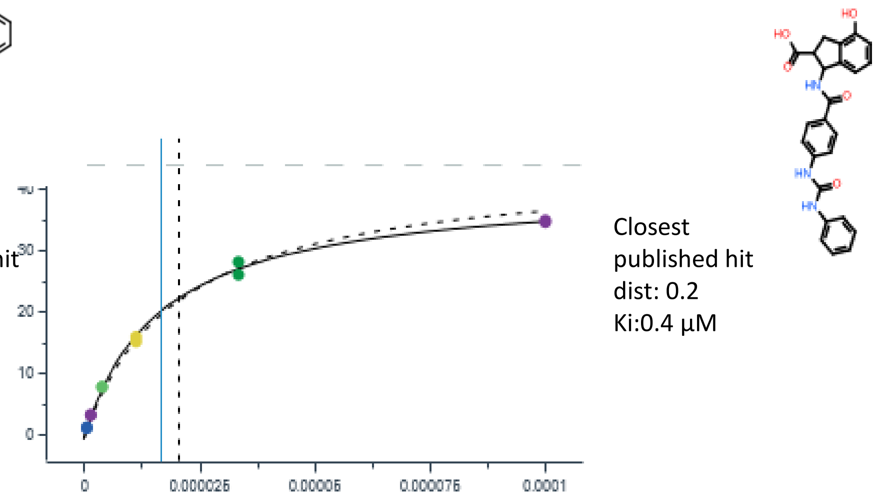

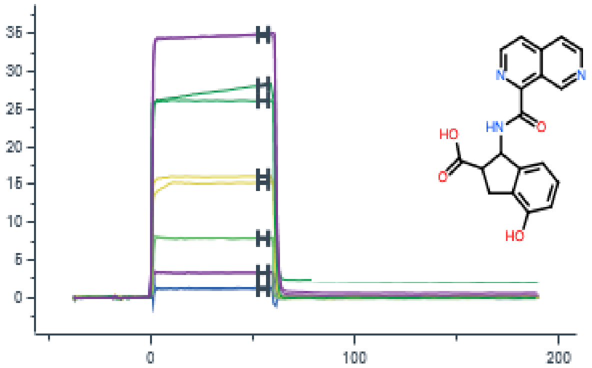

CACHE3-HO_1700_2

K_D = 17 µM– 92% binding
Selectivity for NSP3 (against PARP14a protein) – Yes
DLS (solub@100 µM)
HTRF_displacement hit confirmation (4% DMSO):
%inh@100 uM = 73
%inh@30 uM = 45
%inh@10 uM = 25

CACHE#3 – SARS-CoV2 Nsp3 macrodomain
Participant 1705

PARENT MOLECULE

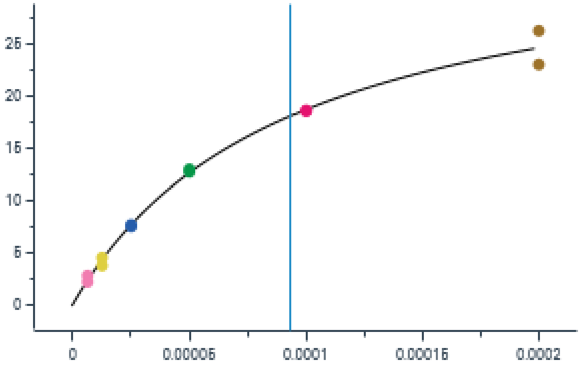

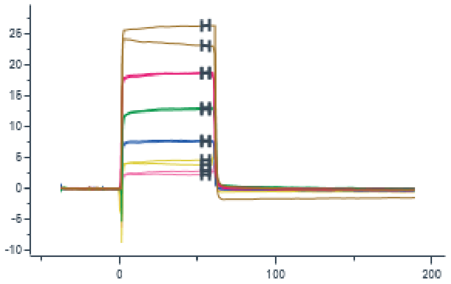

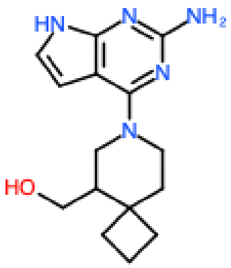

CACHE3HI_1705_4

K_D = 93 µM – 109% binding

Selectivity for NSP3 (against PARP14a protein) – Yes

DLS (solub@100 µM)

HTRF_displacement:

%inh@100 uM = 38

%inh@50 uM = 20

%inh@25 uM = 10

Structure – Yes !

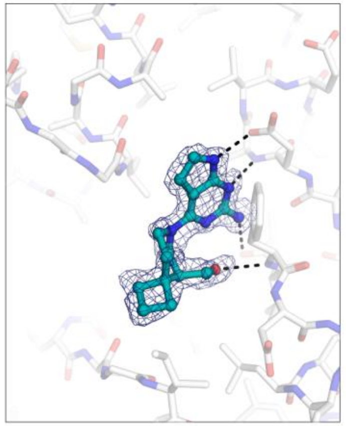

Blue mesh: PanDDA event map contoured at 2 σ

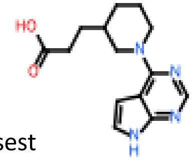

Closest published hit
dist: 0.2
Ki:438 µM

35 analogs of CACHE3HI_1705_4 chemotype were submitted for round 2.
9 compounds showed a dose depended displacement of ADP-ribose peptide by HTRF. Among them **3 compounds** showed very low binding response by SPR.

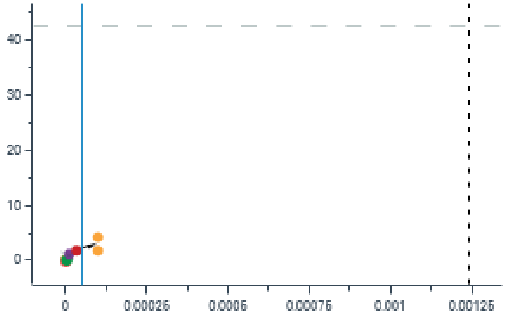

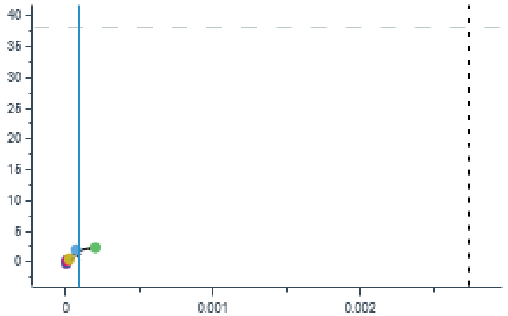

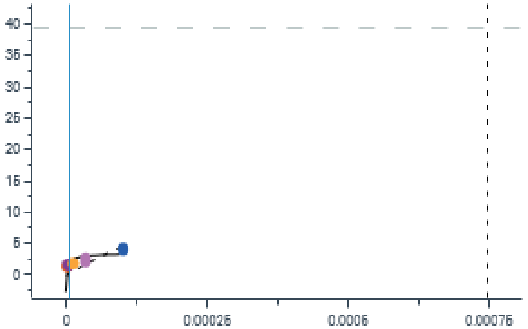

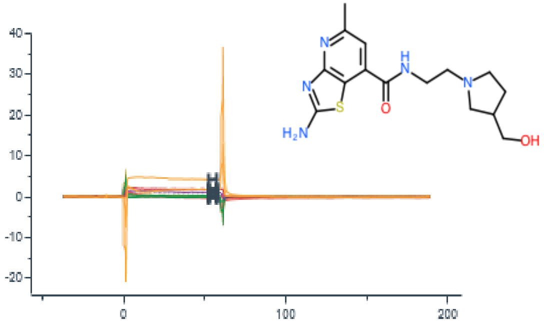

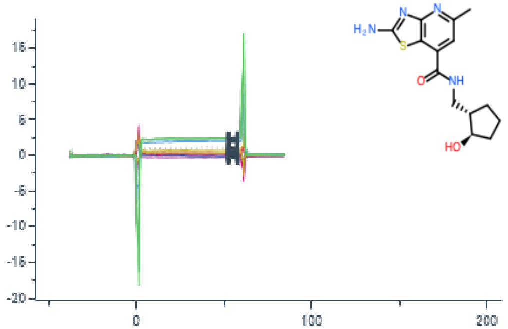

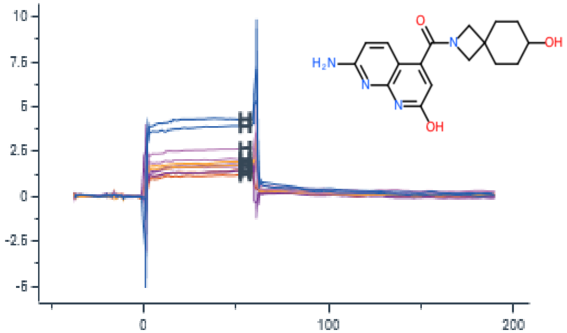

CACHE3-HO_1705_11

K_D = 52 µM (low binding) – 11% binding

DLS (solub@100 µM)

HTRF_displacement hit confirmation(4% DMSO):

%inh@100 uM = 39

%inh@30 uM = 20

%inh@10 uM = 6

CACHE3-HO_1705_40

K_D = 87 µM (low binding) – 9% binding

DLS (solub@100 µM)

HTRF_displacement hit confirmation (4% DMSO):

%inh@100 uM = 25

%inh@30 uM = 11

%inh@10 uM = 7

CACHE3-HO_1705_32

K_D = 4 µM (low binding) – 9% binding

DLS (solub@100 µM)

HTRF_displacement hit confirmation (4% DMSO):

%inh@100 uM = 81

%inh@30 uM = 72

%inh@10 uM = 64

Tested analogs

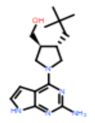

CACHE_ID CACHE3-HO_1705_5
Parent CACHE3HI_1705_4
distance 0.1416

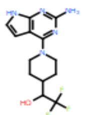

CACHE_ID CACHE3-HO_1705_1
Parent CACHE3HI_1705_4
distance 0.1696

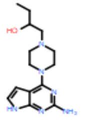

CACHE_ID CACHE3-HO_1705_34
Parent CACHE3HI_1705_4
distance 0.2124

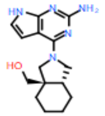

CACHE_ID CACHE3-HO_1705_9
Parent CACHE3HI_1705_4
distance 0.2294

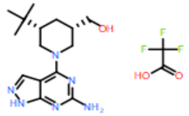

CACHE_ID CACHE3-HO_1705_42
Parent CACHE3HI_1705_4
distance 0.3374

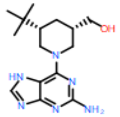

CACHE_ID CACHE3-HO_1705_13
Parent CACHE3HI_1705_4
distance 0.3568

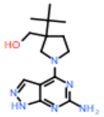

CACHE_ID CACHE3-HO_1705_25
Parent CACHE3HI_1705_4
distance 0.3592

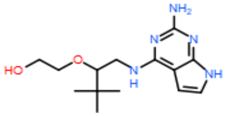

CACHE_ID CACHE3-HO_1705_28
Parent CACHE3HI_1705_4
distance 0.4039

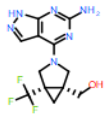

CACHE_ID CACHE3-HO_1705_41
Parent CACHE3HI_1705_4
distance 0.4333

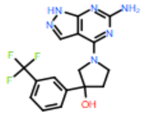

CACHE_ID CACHE3-HO_1705_18
Parent CACHE3HI_1705_4
distance 0.4567

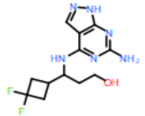

CACHE_ID CACHE3-HO_1705_38
Parent CACHE3HI_1705_4
distance 0.5037

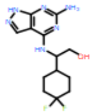

CACHE_ID CACHE3-HO_1705_21
Parent CACHE3HI_1705_4
distance 0.5073

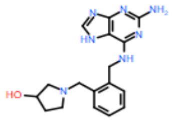

CACHE_ID CACHE3-HO_1705_15
Parent CACHE3HI_1705_4
distance 0.521

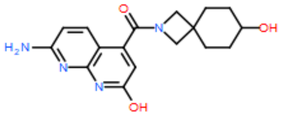

CACHE_ID CACHE3-HO_1705_32
Parent CACHE3HI_1705_4
distance 0.542

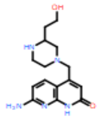

CACHE_ID CACHE3-HO_1705_37
Parent CACHE3HI_1705_4
distance 0.5532

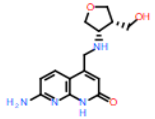

CACHE_ID CACHE3-HO_1705_31
Parent CACHE3HI_1705_4
distance 0.5886

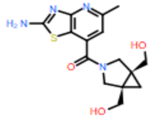

CACHE_ID CACHE3-HO_1705_12
Parent CACHE3HI_1705_4
distance 0.6018

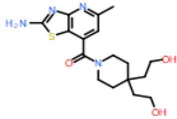

CACHE_ID CACHE3-HO_1705_23
Parent CACHE3HI_1705_4
distance 0.6037

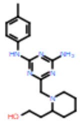

CACHE_ID CACHE3-HO_1705_4
Parent CACHE3HI_1705_4
distance 0.6038

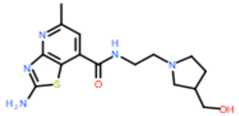

CACHE_ID CACHE3-HO_1705_11
Parent CACHE3HI_1705_4
distance 0.6145

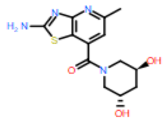

CACHE_ID CACHE3-HO_1705_20
Parent CACHE3HI_1705_4
distance 0.6166

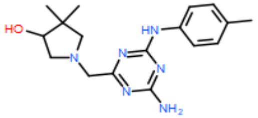

CACHE_ID CACHE3-HO_1705_7
Parent CACHE3HI_1705_4
distance 0.6183

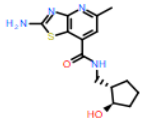

CACHE_ID CACHE3-HO_1705_40
Parent CACHE3HI_1705_4
distance 0.6292

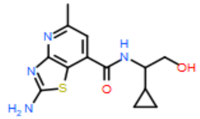

CACHE_ID CACHE3-HO_1705_30
Parent CACHE3HI_1705_4
distance 0.6314

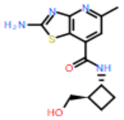

CACHE_ID CACHE3-HO_1705_24
Parent CACHE3HI_1705_4
distance 0.6322

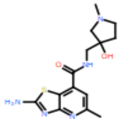

CACHE_ID CACHE3-HO_1705_8
Parent CACHE3HI_1705_4
distance 0.6353

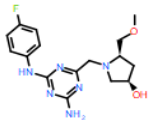

CACHE_ID CACHE3-HO_1705_6
Parent CACHE3HI_1705_4
distance 0.6373

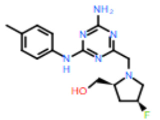

CACHE_ID CACHE3-HO_1705_33
Parent CACHE3HI_1705_4
distance 0.6406

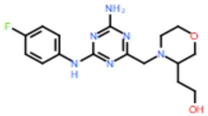

CACHE_ID CACHE3-HO_1705_26
Parent CACHE3HI_1705_4
distance 0.6502

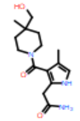

CACHE_ID CACHE3-HO_1705_35
Parent CACHE3HI_1705_4
distance 0.6606

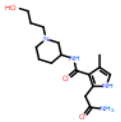

CACHE_ID CACHE3-HO_1705_3
Parent CACHE3HI_1705_4
distance 0.6858

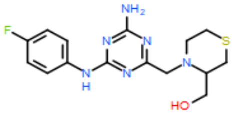

CACHE_ID CACHE3-HO_1705_22
Parent CACHE3HI_1705_4
distance 0.6912

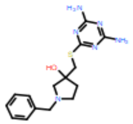

CACHE_ID CACHE3-HO_1705_39
Parent CACHE3HI_1705_4
distance 0.692

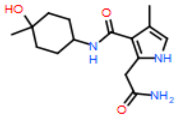

CACHE_ID CACHE3-HO_1705_14
Parent CACHE3HI_1705_4
distance 0.6926

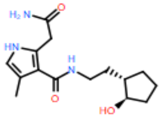

CACHE_ID CACHE3-HO_1705_17
Parent CACHE3HI_1705_4
distance 0.6996

CACHE#3 – SARS-CoV2 Nsp3 macrodomain
Participant 1706

PARENT MOLECULE

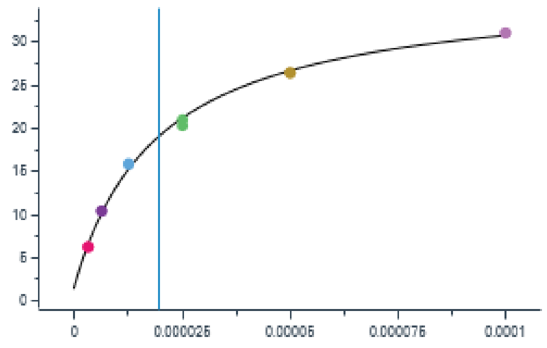

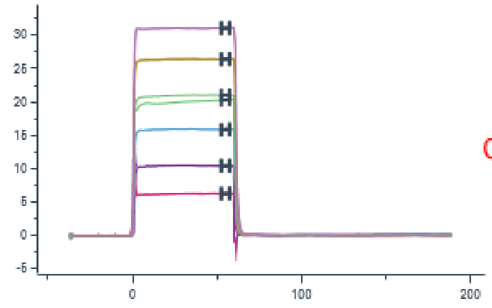

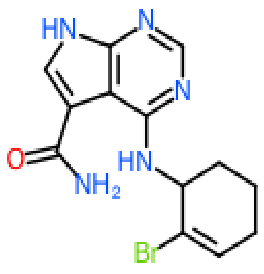

18 analogs, including parent molecule, of CACHE3HI_1706_56 chemotype were submitted for round 2.
17 compounds, including a re-supplied parent molecule, showed a dose depended displacement of ADP-ribose peptide by HTRF. Among them **12 compounds** confirmed a dose dependent binding response by SPR, reaching saturation. **1 compound** did not reach saturation.

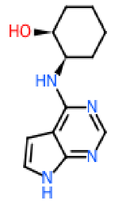

Closest published hit
dist: 0.3
Ki:114 µM

CACHE3-HO_1706_20

K$_D$ = 10 µM (poor fit) – 98% binding
DLS (solub@100 µM)
HTRF_displacement hit confirmation (4% DMSO):
%inh@100 uM = 81
%inh@30 uM = 47
%inh@10 uM = 29

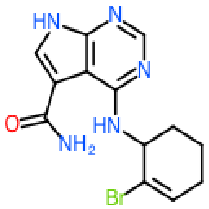

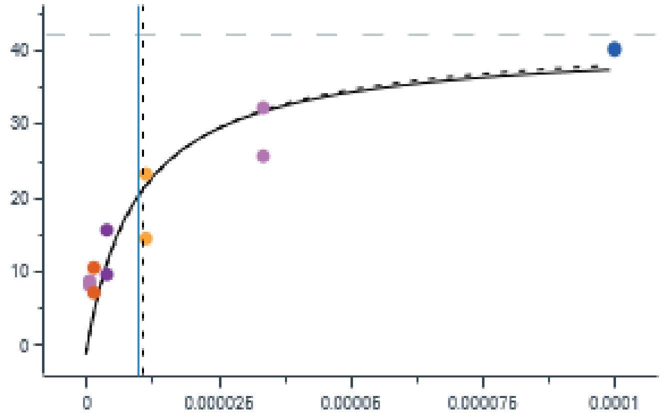

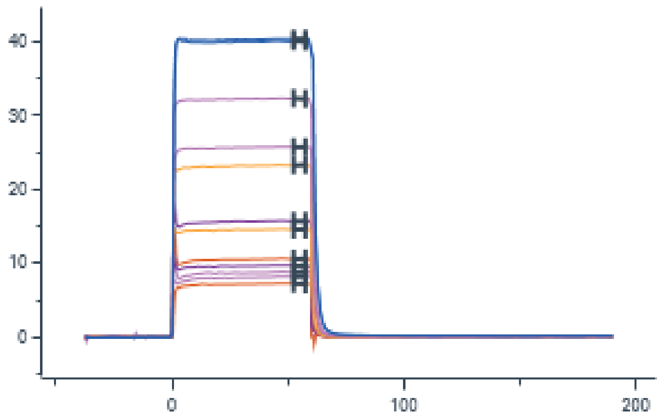

CACHE3HI_1706_56

K$_D$ = 20 µM – 89% binding
Selectivity for NSP3 (against PARP14a protein) – Yes
DLS (solub@100 µM)
HTRF_displacement:
%inh@100 uM = 84
%inh@50 uM = 72
%inh@25 uM = 54

Structure – Yes !

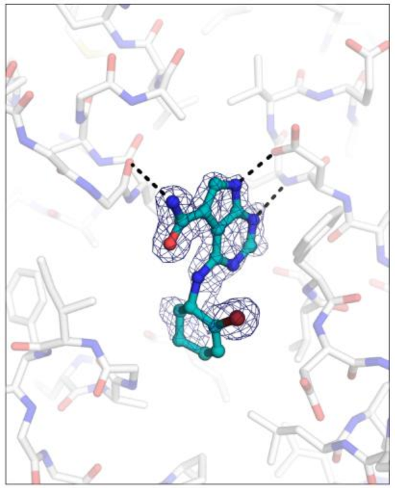

Blue mesh: PanDDA event map contoured at 2 σ

Tested analogs

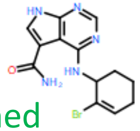

confirmed

CACHE_ID	CACHE3-HO_1706_20
Parent	CACHE3HI_1706_56
distance	0

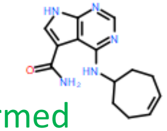

confirmed

CACHE_ID	CACHE3-HO_1706_3
Parent	CACHE3HI_1706_56
distance	0.1235

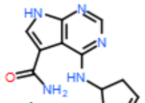

confirmed

CACHE_ID	CACHE3-HO_1706_10
Parent	CACHE3HI_1706_56
distance	0.1469

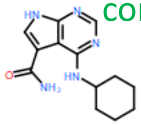

confirmed

CACHE_ID	CACHE3-HO_1706_7
Parent	CACHE3HI_1706_56
distance	0.1535

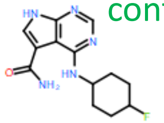

confirmed

CACHE_ID	CACHE3-HO_1706_1
Parent	CACHE3HI_1706_56
distance	0.1613

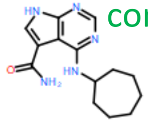

confirmed

CACHE_ID	CACHE3-HO_1706_4
Parent	CACHE3HI_1706_56
distance	0.1646

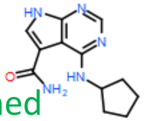

confirmed

CACHE_ID	CACHE3-HO_1706_11
Parent	CACHE3HI_1706_56
distance	0.1653

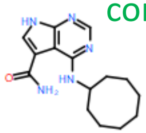

confirmed

CACHE_ID	CACHE3-HO_1706_2
Parent	CACHE3HI_1706_56
distance	0.168

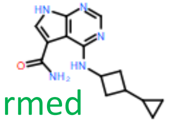

confirmed

CACHE_ID	CACHE3-HO_1706_12
Parent	CACHE3HI_1706_56
distance	0.1748

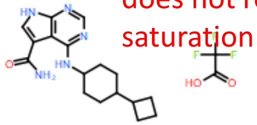

does not reach saturation

CACHE_ID	CACHE3-HO_1706_18
Parent	CACHE3HI_1706_56
distance	0.1774

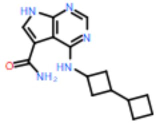

CACHE_ID	CACHE3-HO_1706_14
Parent	CACHE3HI_1706_56
distance	0.1781

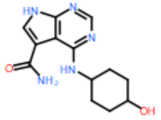

CACHE_ID	CACHE3-HO_1706_6
Parent	CACHE3HI_1706_56
distance	0.1873

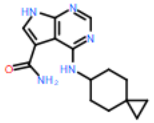

CACHE_ID	CACHE3-HO_1706_15
Parent	CACHE3HI_1706_56
distance	0.2317

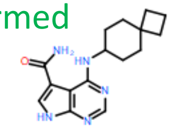

confirmed

CACHE_ID	CACHE3-HO_1706_16
Parent	CACHE3HI_1706_56
distance	0.2317

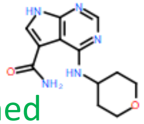

confirmed

CACHE_ID	CACHE3-HO_1706_9
Parent	CACHE3HI_1706_56
distance	0.2326

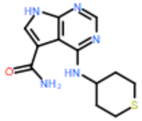

CACHE_ID	CACHE3-HO_1706_5
Parent	CACHE3HI_1706_56
distance	0.2326

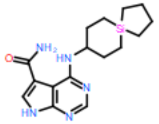

CACHE_ID	CACHE3-HO_1706_19
Parent	CACHE3HI_1706_56
distance	0.2395

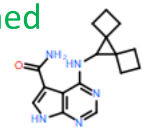

confirmed

CACHE_ID	CACHE3-HO_1706_8
Parent	CACHE3HI_1706_56
distance	0.3004

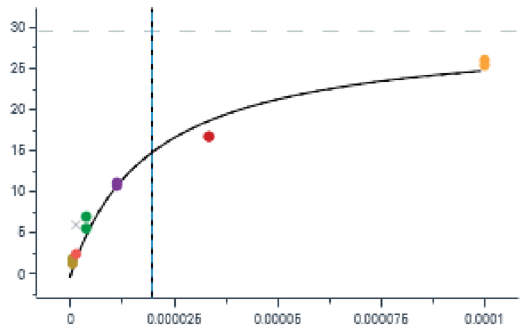

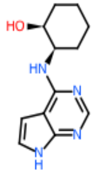

Closest published hit
dist: 0.2
Ki:114 µM

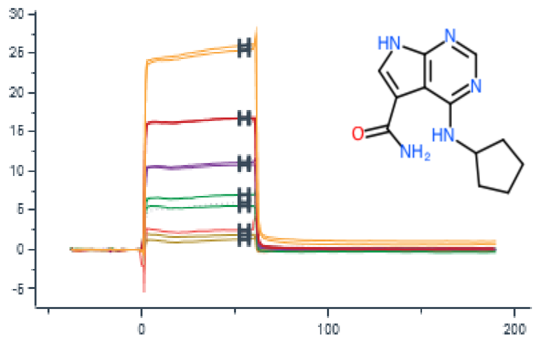

CACHE3-HO_1706_11

K_D (run 1) = 20 µM – 100% binding
K_D (run 2) = 34 µM – 116% binding
Selectivity for NSP3 (against PARP14a protein) – 45 µM – 87% binding
DLS (solub@100 µM)
HTRF_displacement hit confirmation (4% DMSO):
%inh@100 uM = 63
%inh@30 uM = 26
%inh@10 uM = 18

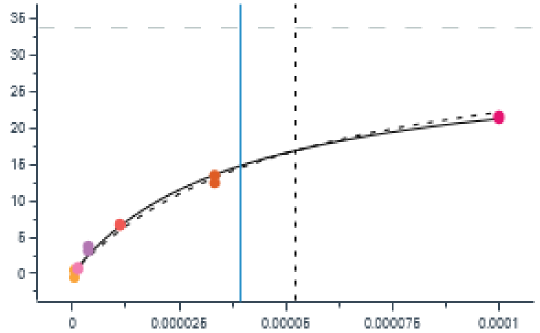

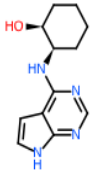

Closest published hit
dist: 0.3
Ki:114 µM

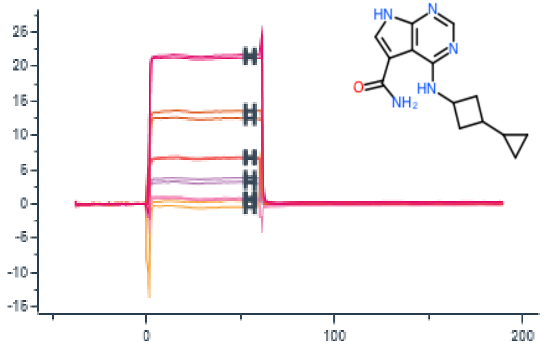

CACHE3-HO_1706_12

K_D (run 1) = 40 µM – 88% binding
K_D (run 2) = 59 µM – 134% binding
Selectivity for NSP3 (against PARP14a protein) – Yes
DLS (solub@100 µM)
HTRF_displacement hit confirmation (4% DMSO):
%inh@100 uM = 57
%inh@30 uM = 24
%inh@10 uM = 17

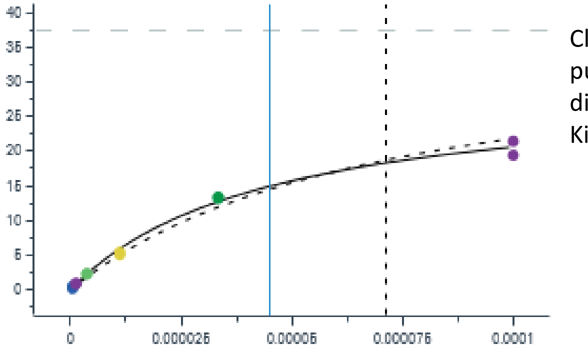

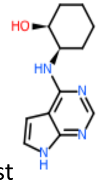

Closest published hit
dist: 0.4
Ki:114 µM

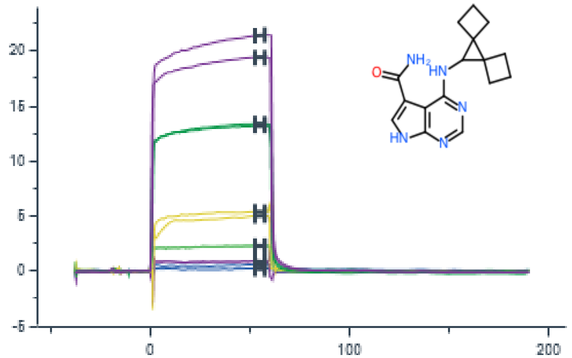

CACHE3-HO_1706_8

K_D = 45 µM – 80% binding
Selectivity for NSP3 (against PARP14a protein) – mild binding (linear response)
DLS (solub@100 µM)
HTRF_displacement hit confirmation (4% DMSO):
%inh@100 uM = 50
%inh@30 uM = 22
%inh@10 uM = 13

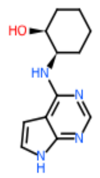

Closest
published hit
dist: 0.3
Ki:114 µM

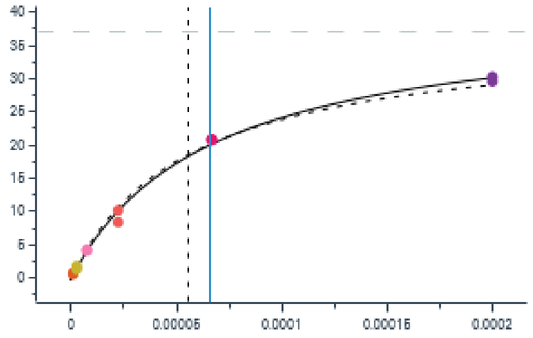

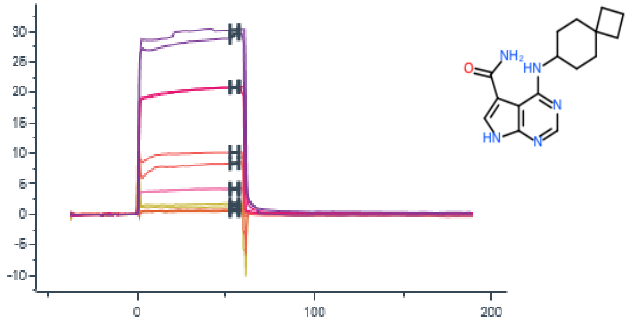

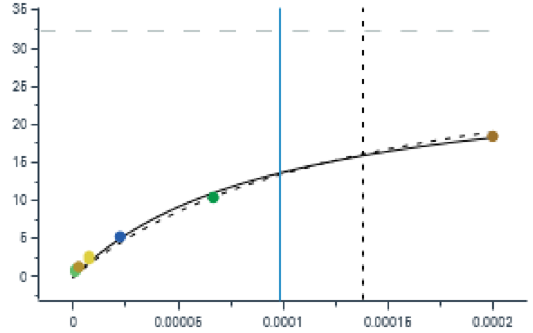

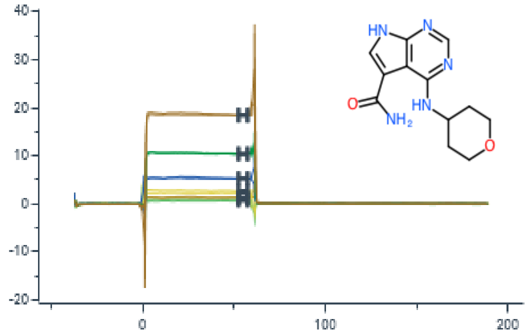

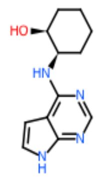

Closest
published hit
dist: 0.3
Ki:114 µM

CACHE3-HO_1706_16

K_D (run 1) = 51 µM – 70% binding
K_D (run 2) = 66 µM – 108% binding
Selectivity for NSP3 (against PARP14a protein) – Yes
DLS (solub@100 µM)
HTRF_displacement hit confirmation (4% DMSO):
%inh@100 uM = 53
%inh@30 uM = 23
%inh@10 uM = 15

CACHE3-HO_1706_9

K_D = 99 µM – 85% binding
Selectivity for NSP3 (against PARP14a protein) – Yes
DLS (solub@100 µM)
HTRF_displacement hit confirmation (4% DMSO):
%inh@100 uM = 25
%inh@30 uM = 10
%inh@10 uM = 8

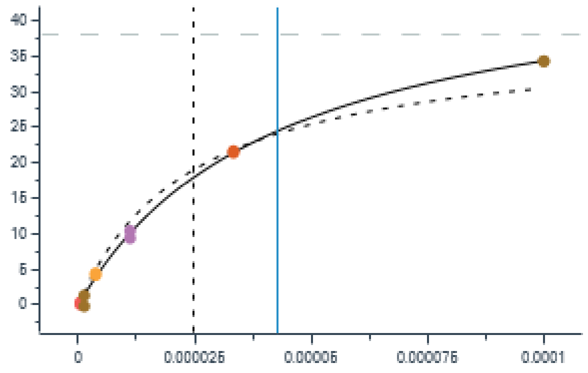

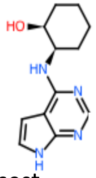

Closest published hit dist: 0.3 Ki:114 µM

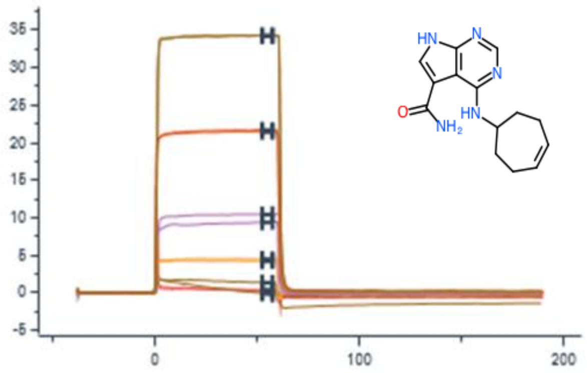

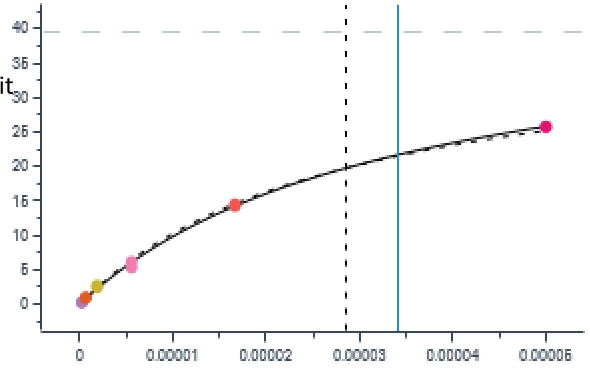

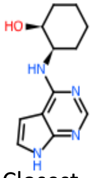

Closest published hit dist: 0.26 Ki:114 µM

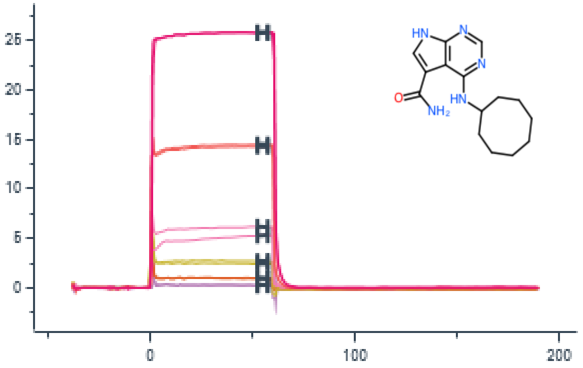

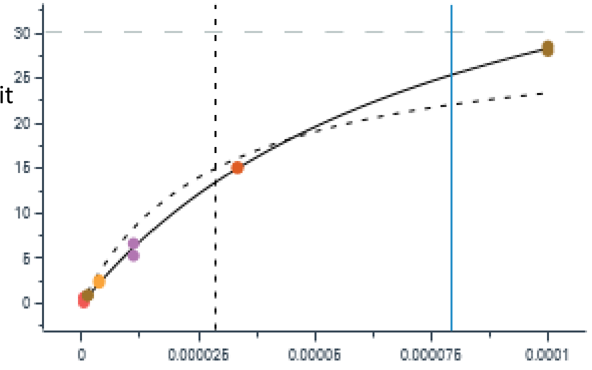

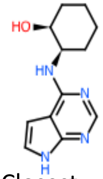

Closest published hit dist: 0.3 Ki:114 µM

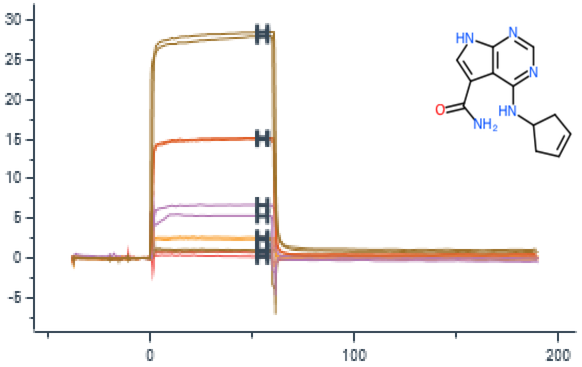

CACHE3-HO_1706_3

K_D (run 1) = 60 µM – 143% binding
K_D (run 2) = 43 µM – 129% binding
Selectivity for NSP3 (against PARP14a protein) – Yes
DLS (solub@50 µM)
HTRF_displacement hit confirmation (4% DMSO):
%inh@100 uM = 61
%inh@30 uM = 30
%inh@10 uM = 6

CACHE3-HO_1706_2

K_D = 34 µM – 110% binding
Selectivity for NSP3 (against PARP14a protein) – Yes
DLS (solub@50 µM)
HTRF_displacement hit confirmation (4% DMSO):
%inh@100 uM = 67
%inh@30 uM = 34
%inh@10 uM = 14

CACHE3-HO_1706_10

K_D (run 1) = 81 µM (predicted) – 146% binding
K_D (run 2) = 79 µM – 169% binding
Selectivity for NSP3 (against PARP14a protein) – Yes
DLS (solub@50 µM)
HTRF_displacement hit confirmation (4% DMSO):
%inh@100 uM = 49
%inh@30 uM = 20
%inh@10 uM = 16

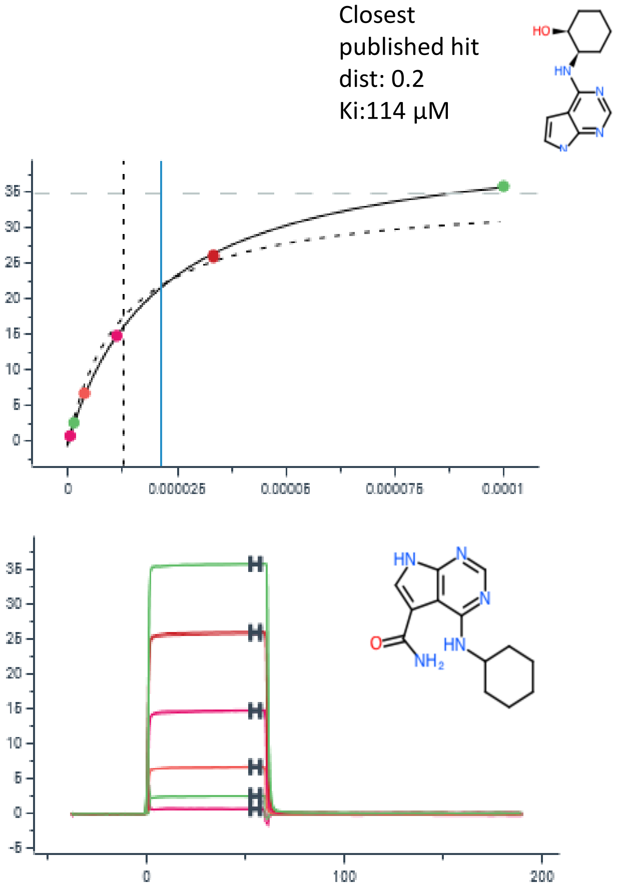

CACHE3-HO_1706_7

K_D (run 1) = 24 µM – 108% binding
K_D (run 1) = 21 µM – 125% binding
Selectivity for NSP3 (against PARP14a protein) – Yes
DLS (solub@50 µM)
HTRF_displacement hit confirmation (4% DMSO):
%inh@100 uM = 75
%inh@30 uM = 39
%inh@10 uM = 26

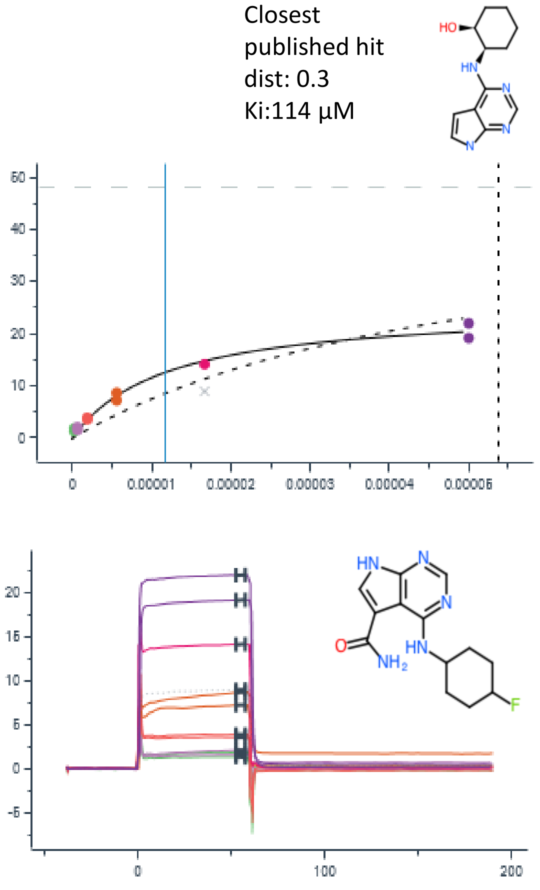

CACHE3-HO_1706_1

K_D = 12 µM – 53% binding
Selectivity for NSP3 (against PARP14a protein) – Yes
DLS (solub@50 µM)
HTRF_displacement hit confirmation (4% DMSO):
%inh@100 uM = 54
%inh@30 uM = 22
%inh@10 uM = 11

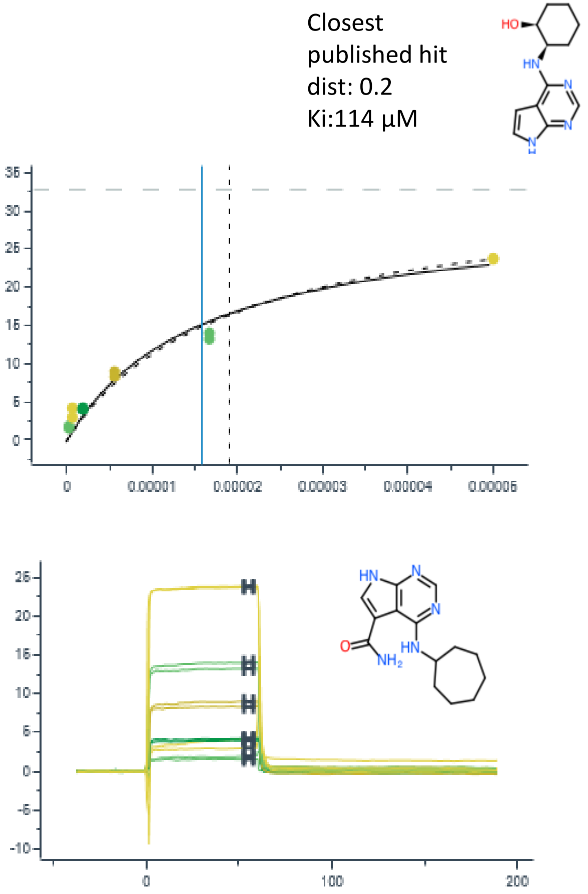

CACHE3-HO_1706_4

K_D (run 1) = 16 µM – 92% binding
K_D (run 2) = 25 µM – 128% binding
Selectivity for NSP3 (against PARP14a protein) – Yes
DLS (solub@50 µM)
HTRF_displacement hit confirmation (4% DMSO):
%inh@100 uM = 76
%inh@30 uM = 42
%inh@10 uM = 29

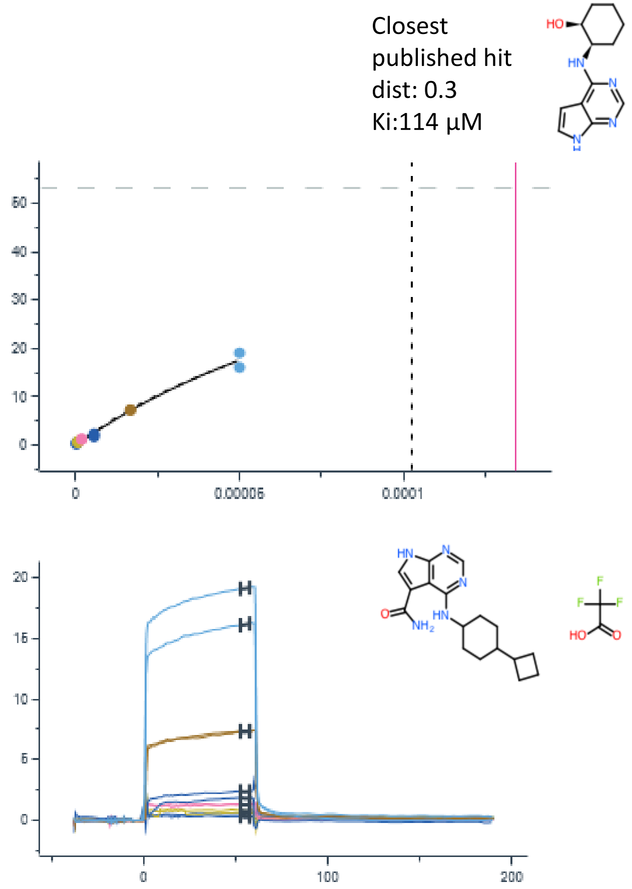

CACHE3-HO_1706_18

K_D = 134 µM (does not reach saturation) – 122% binding
DLS (solub@50 µM)
HTRF_displacement hit confirmation (4% DMSO):
%inh@100 uM = 40
%inh@30 uM = 19
%inh@10 uM = 4

CACHE#3 – SARS-CoV2 Nsp3 macrodomain
Participant 1708

PARENT MOLECULE

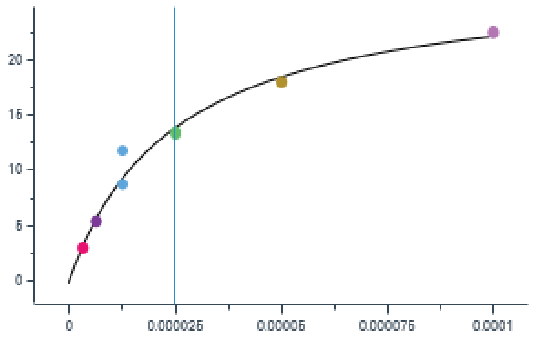

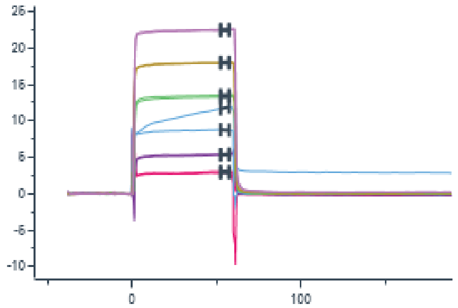

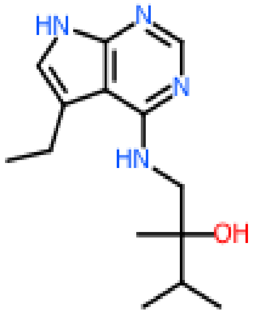

CACHE3HI_1708_42

K_D = 25 μM – 114% binding

Selectivity for NSP3 (against PARP14a protei[illegible]

DLS (solub@100 μM)

HTRF_displacement:

%inh@100 uM = 66

%inh@50 uM = 46

%inh@25 uM = 34

Structure – Yes !

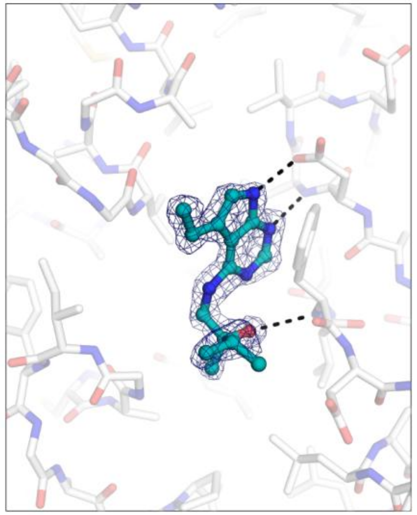

Blue mesh: PanDDA event map contoured at 2 σ

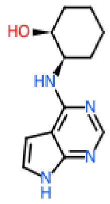

Closest published hit
dist: 0.2
Ki:114 μM

40 analogs, including parent molecule, of <u>CACHE3HI_1708_42 chemotype</u> were submitted for round 2.

23 compounds, including a re-supplied parent molecule, showed a dose depended displacement of ADP-ribose peptide by HTRF. Among them **11 compounds** confirmed a dose dependent binding response by SPR with a tendency to reach saturation

CACHE3-HO_1708_1

K_D = 11 μM (poor fit) – 83% binding

DLS (solub@100 μM)

HTRF_displacement hit confirmation (4% DMSO):

%inh@100 uM = 71

%inh@30 uM = 42

%inh@10 uM = 17

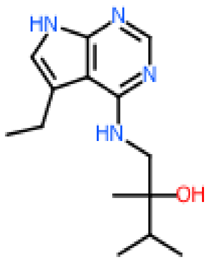

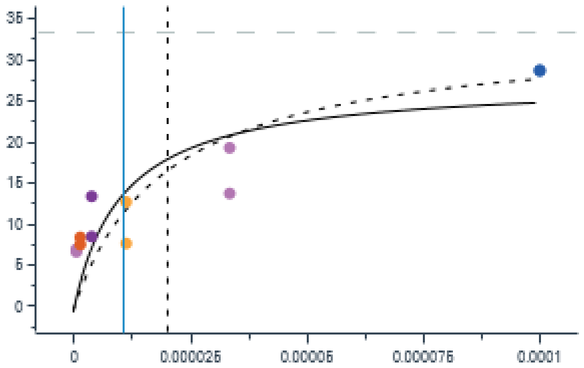

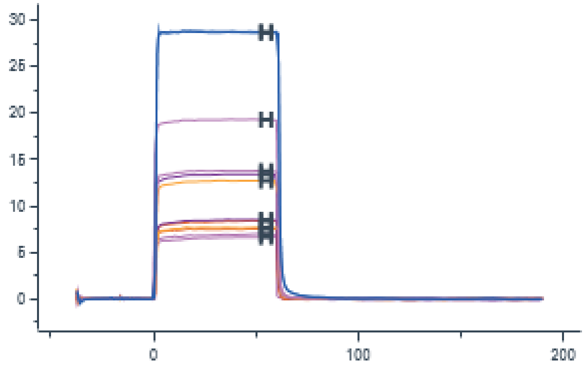

Tested analogs

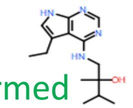
confirmed
CACHE_ID CACHE3-HO_1708_1
Parent CACHE3HI_1708_42
distance 0

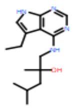
CACHE_ID CACHE3-HO_1708_34
Parent CACHE3HI_1708_42
distance 0.03865

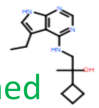
confirmed
CACHE_ID CACHE3-HO_1708_41
Parent CACHE3HI_1708_42
distance 0.06977

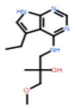
CACHE_ID CACHE3-HO_1708_31
Parent CACHE3HI_1708_42
distance 0.07583

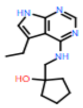
CACHE_ID CACHE3-HO_1708_37
Parent CACHE3HI_1708_42
distance 0.07944

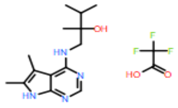
CACHE_ID CACHE3-HO_1708_46
Parent CACHE3HI_1708_42
distance 0.1048

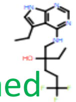
confirmed
CACHE_ID CACHE3-HO_1708_11
Parent CACHE3HI_1708_42
distance 0.1126

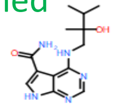
confirmed
CACHE_ID CACHE3-HO_1708_5
Parent CACHE3HI_1708_42
distance 0.1532

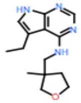
CACHE_ID CACHE3-HO_1708_44
Parent CACHE3HI_1708_42
distance 0.1562

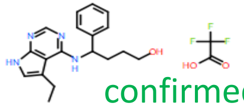
confirmed
CACHE_ID CACHE3-HO_1708_32
Parent CACHE3HI_1708_42
distance 0.1983

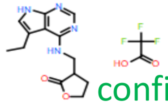
confirmed
CACHE_ID CACHE3-HO_1708_26
Parent CACHE3HI_1708_42
distance 0.2176

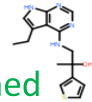
confirmed
CACHE_ID CACHE3-HO_1708_35
Parent CACHE3HI_1708_42
distance 0.2204

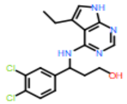
CACHE_ID CACHE3-HO_1708_25
Parent CACHE3HI_1708_42
distance 0.2282

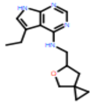
CACHE_ID CACHE3-HO_1708_43
Parent CACHE3HI_1708_42
distance 0.2298

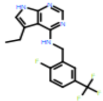
CACHE_ID CACHE3-HO_1708_21
Parent CACHE3HI_1708_42
distance 0.2479

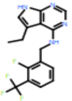
CACHE_ID CACHE3-HO_1708_22
Parent CACHE3HI_1708_42
distance 0.251

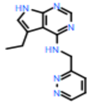
CACHE_ID CACHE3-HO_1708_49
Parent CACHE3HI_1708_42
distance 0.2625

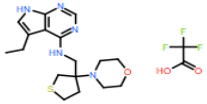
CACHE_ID CACHE3-HO_1708_27
Parent CACHE3HI_1708_42
distance 0.2879

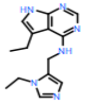
CACHE_ID CACHE3-HO_1708_28
Parent CACHE3HI_1708_42
distance 0.3028

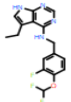
CACHE_ID CACHE3-HO_1708_20
Parent CACHE3HI_1708_42
distance 0.305

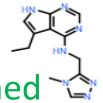
confirmed
CACHE_ID CACHE3-HO_1708_45
Parent CACHE3HI_1708_42
distance 0.316

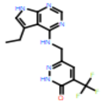
CACHE_ID CACHE3-HO_1708_12
Parent CACHE3HI_1708_42
distance 0.3382

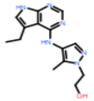
CACHE_ID CACHE3-HO_1708_33
Parent CACHE3HI_1708_42
distance 0.354

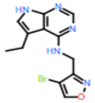
CACHE_ID CACHE3-HO_1708_29
Parent CACHE3HI_1708_42
distance 0.361

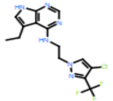
CACHE_ID CACHE3-HO_1708_18
Parent CACHE3HI_1708_42
distance 0.3684

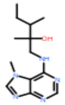
CACHE_ID CACHE3-HO_1708_3
Parent CACHE3HI_1708_42
distance 0.4153

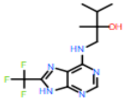
CACHE_ID CACHE3-HO_1708_38
Parent CACHE3HI_1708_42
distance 0.418

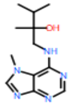
CACHE_ID CACHE3-HO_1708_2
Parent CACHE3HI_1708_42
distance 0.4224

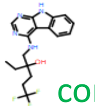
confirmed
CACHE_ID CACHE3-HO_1708_15
Parent CACHE3HI_1708_42
distance 0.4245

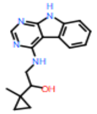
CACHE_ID CACHE3-HO_1708_42
Parent CACHE3HI_1708_42
distance 0.4255

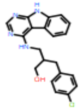
CACHE_ID CACHE3-HO_1708_17
Parent CACHE3HI_1708_42
distance 0.4507

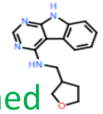
confirmed
CACHE_ID CACHE3-HO_1708_39
Parent CACHE3HI_1708_42
distance 0.4571

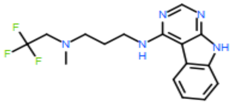
CACHE_ID CACHE3-HO_1708_19
Parent CACHE3HI_1708_42
distance 0.4894

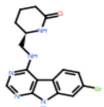
CACHE_ID CACHE3-HO_1708_50
Parent CACHE3HI_1708_42
distance 0.5081

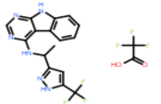
CACHE_ID CACHE3-HO_1708_9
Parent CACHE3HI_1708_42
distance 0.5206

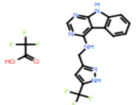
CACHE_ID CACHE3-HO_1708_23
Parent CACHE3HI_1708_42
distance 0.526

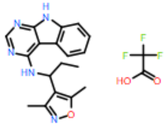
CACHE_ID CACHE3-HO_1708_7
Parent CACHE3HI_1708_42
distance 0.5269

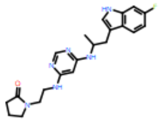
CACHE_ID CACHE3-HO_1708_13
Parent CACHE3HI_1708_42
distance 0.5419

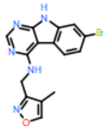
CACHE_ID CACHE3-HO_1708_30
Parent CACHE3HI_1708_42
distance 0.5593

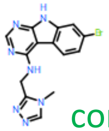
confirmed
CACHE_ID CACHE3-HO_1708_36
Parent CACHE3HI_1708_42
distance 0.5659

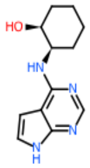

Closest
published hit
dist: 0.2
Ki:114 µM

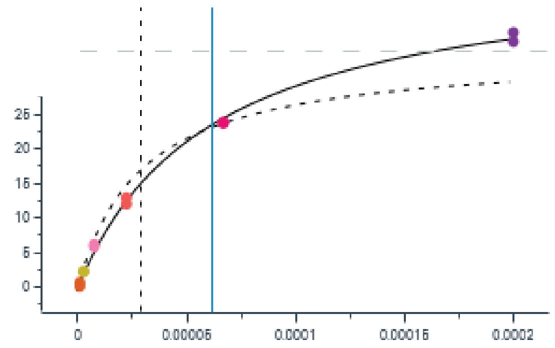

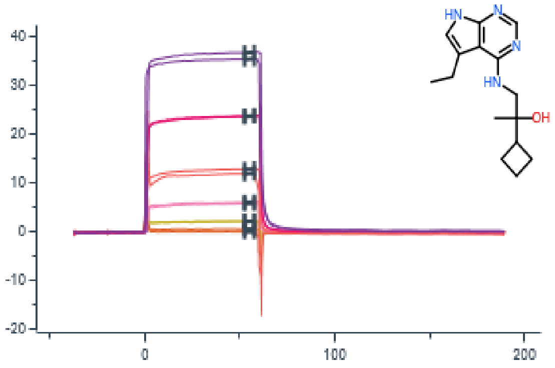

CACHE3-HO_1708_41

K_D (run 1) = 64 µM – 112% binding
K_D (run 2) = 61 µM – 138% binding
Selectivity for NSP3 (against PARP14a protein) – Linear response (slow off)
DLS (solub@100 µM)
HTRF_displacement hit confirmation (4% DMSO):
%inh@100 uM = 45
%inh@30 uM = 15
%inh@10 uM = 14

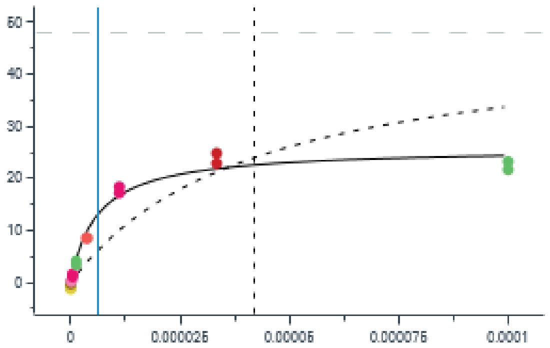

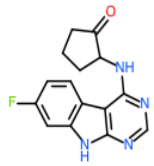

Closest
published hit
dist: 0.3
Ki:12 µM

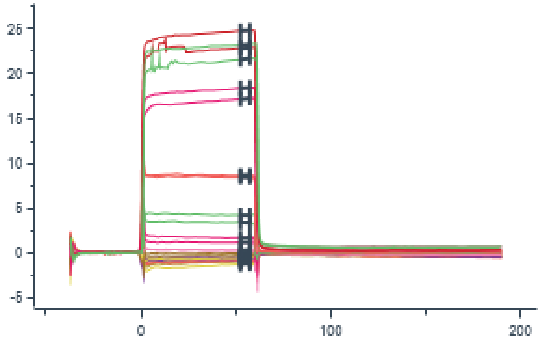

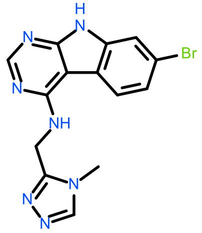

CACHE3-HO_1708_36

K_D = 6 µM – 54% binding
Selectivity for NSP3 (against PARP14a protein) – Yes
DLS (solub@100 µM)
HTRF_displacement hit confirmation (4% DMSO):
%inh@100 uM = 86
%inh@30 uM = 39
%inh@10 uM = 13

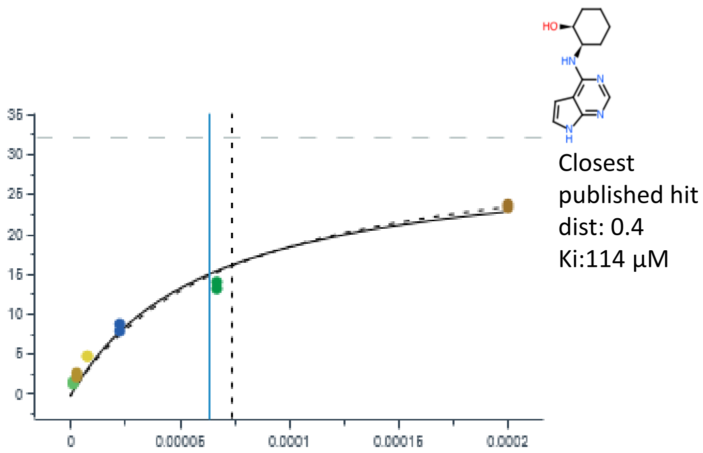

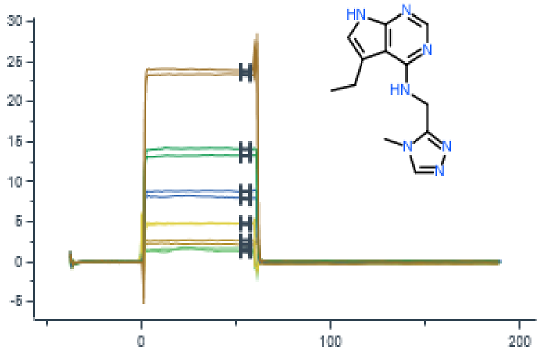

CACHE3-HO_1708_45

K_D (run 1) = 63 µM – 94% binding
K_D (run 2) = 95 µM – 119% binding
Selectivity for NSP3 (against PARP14a protein) – Yes
DLS (solub@100 µM)
HTRF_displacement hit confirmation (4% DMSO):
%inh@100 uM = 25
%inh@30 uM = 10
%inh@10 uM = 6

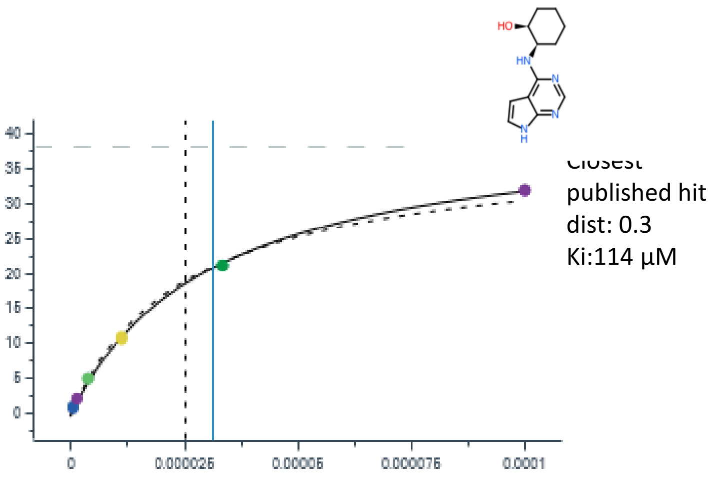

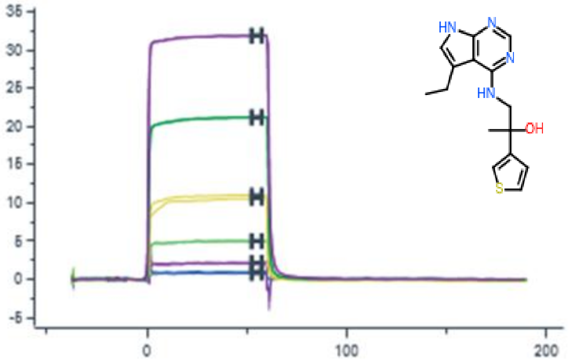

CACHE3-HO_1708_35

K_D (run 1) = 24 µM (poor fit) – 100% binding
K_D (run 2) = 31 µM – 109% binding
Selectivity for NSP3 (against PARP14a protein) – mild binding
DLS (solub@100 µM)
HTRF_displacement hit confirmation (4% DMSO):
%inh@100 uM = 58
%inh@30 uM = 28
%inh@10 uM = 17

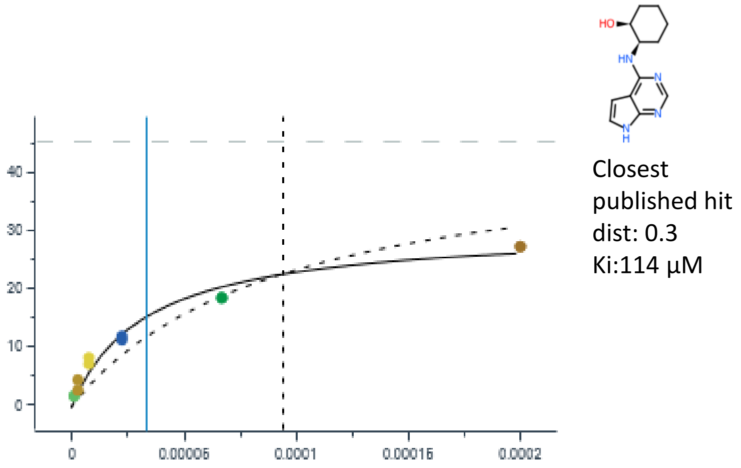

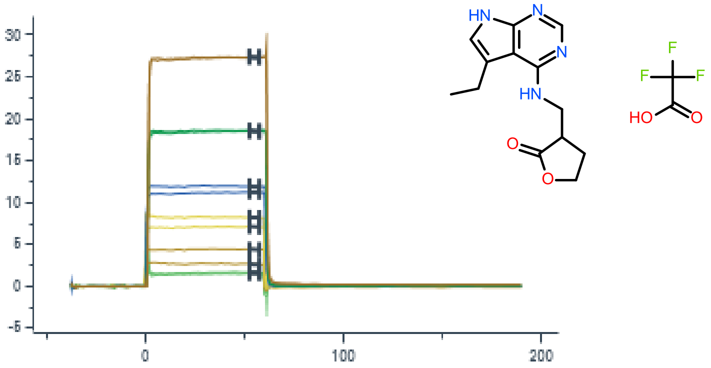

CACHE3-HO_1708_26

K_D (run 1) = 33 µM – 68% binding
K_D (run 2) = 48 µM – 75% binding
Selectivity for NSP3 (against PARP14a protein) – mild binding
DLS (solub@100 µM)
HTRF_displacement hit confirmation (4% DMSO):
%inh@100 uM = 36
%inh@30 uM = 8
%inh@10 uM = 9

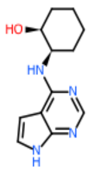

Closest
published hit
dist: 0.3
Ki:114 µM

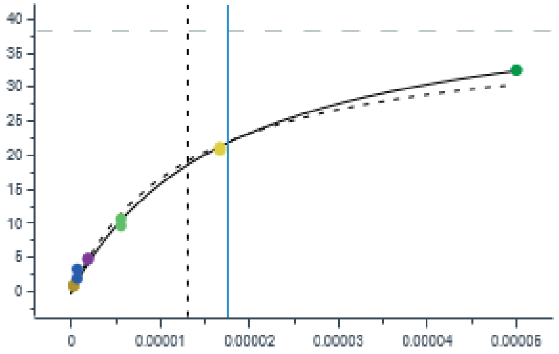

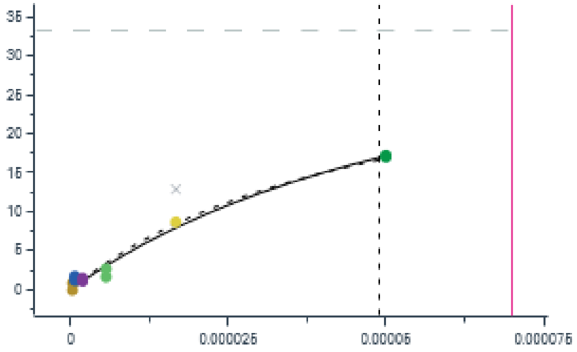

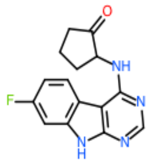

Closest
published hit
dist: 0.2
Ki:12 µM

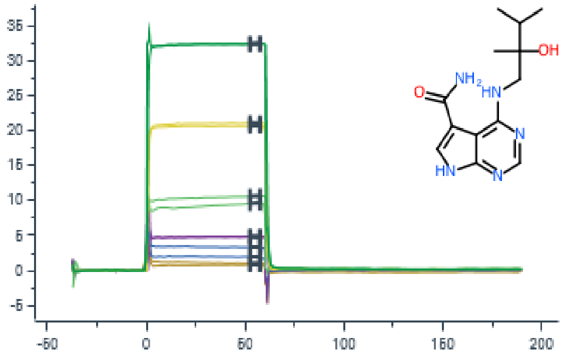

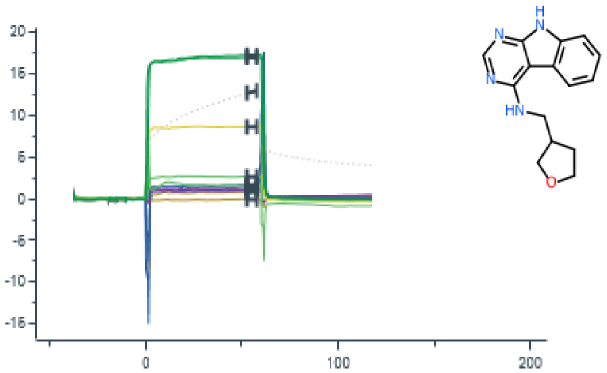

CACHE3-HO_1708_5

K_D = 18 µM – 114% binding
Selectivity for NSP3 (against PARP14a protein) – Yes
DLS (solub@30 µM)
HTRF_displacement hit confirmation (4% DMSO):
%inh@100 uM = 74
%inh@30 uM = 38
%inh@10 uM = 18

CACHE3-HO_1708_39

K_D (run 1) = 27 µM – 50% binding
K_D (run 2) = 70 µM – 124% binding
Selectivity for NSP3 (against PARP14a protein) – Yes
DLS (solub@30 µM)
HTRF_displacement hit confirmation (4% DMSO):
%inh@100 uM = 27
%inh@30 uM = 6
%inh@10 uM = 2

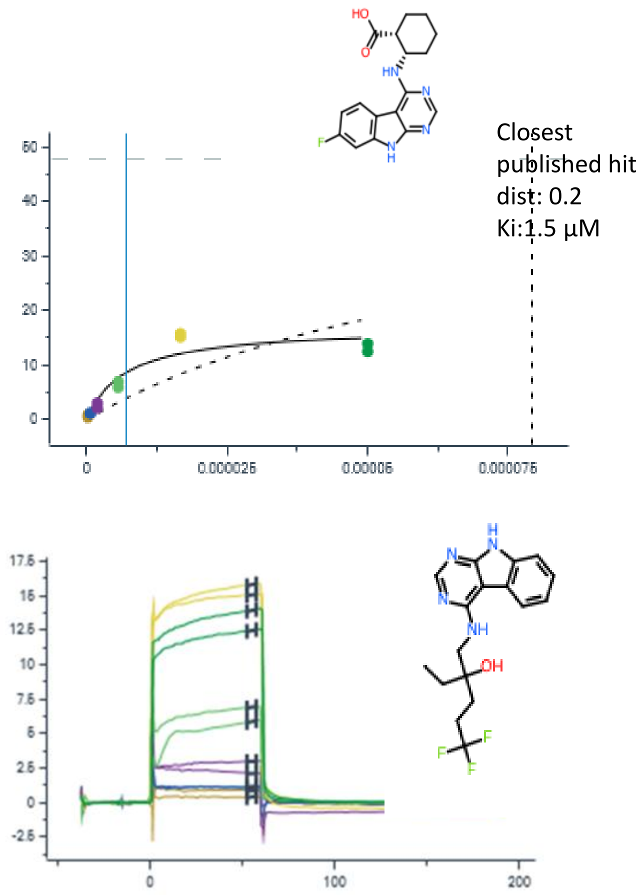

CACHE3-HO_1708_15

K_D (run 1) = 7 µM (poor fit) – 35% binding
K_D (run 2) = 7 µM (poor fit) – 36% binding
Selectivity for NSP3 (against PARP14a protein) – Yes
DLS (solub@50 µM)
HTRF_displacement hit confirmation (4% DMSO):
%inh@100 uM = 74
%inh@30 uM = 24
%inh@10 uM = 15

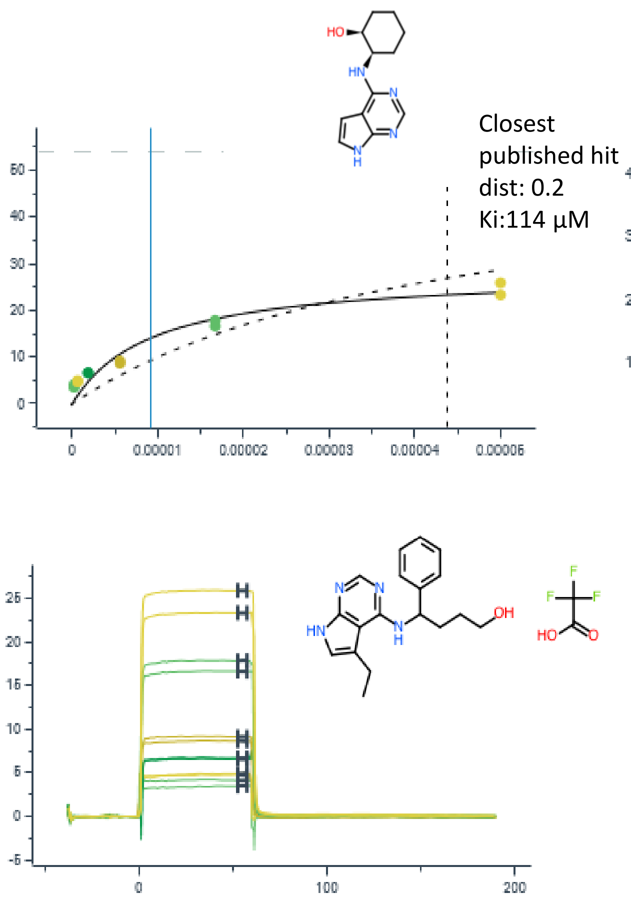

CACHE3-HO_1708_32

K_D (run 1) = 9 µM (poor fit) – 52% binding
K_D (run 2) = 20 µM – 74% binding
Selectivity for NSP3 (against PARP14a protein) – Yes
DLS (solub@50 µM)
HTRF_displacement hit confirmation (4% DMSO):
%inh@100 uM = 59
%inh@30 uM = 21
%inh@10 uM = 13

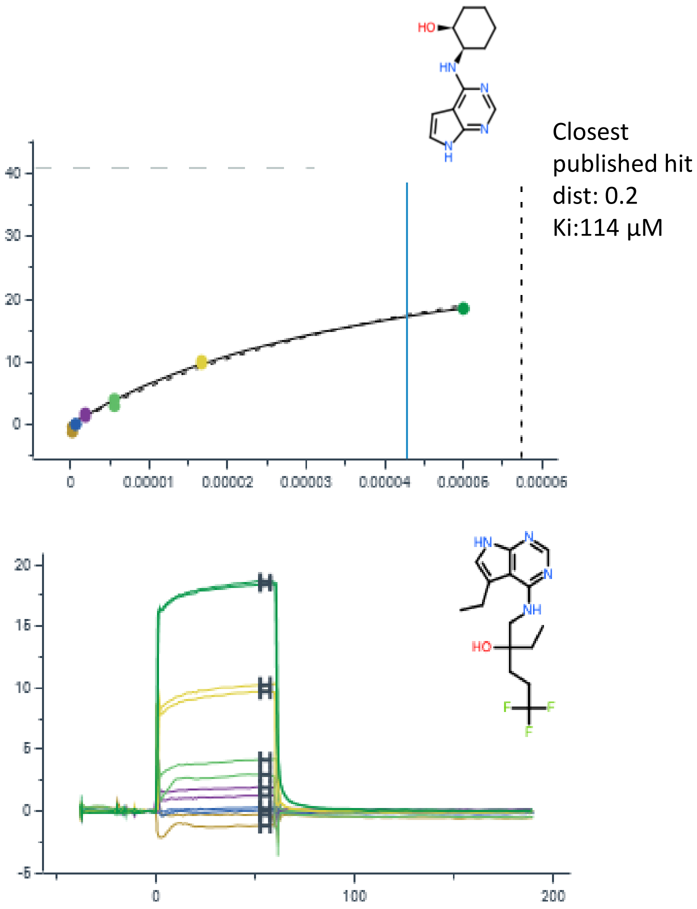

CACHE3-HO_1708_11

K_D = 43 µM – 84% binding
Selectivity for NSP3 (against PARP14a protein) – 43% binding
DLS (solub@50 µM)
HTRF_displacement hit confirmation (4% DMSO):
%inh@100 uM = 55
%inh@30 uM = 26
%inh@10 uM = 13

HIT MOLECULE that was not followed up for round 2

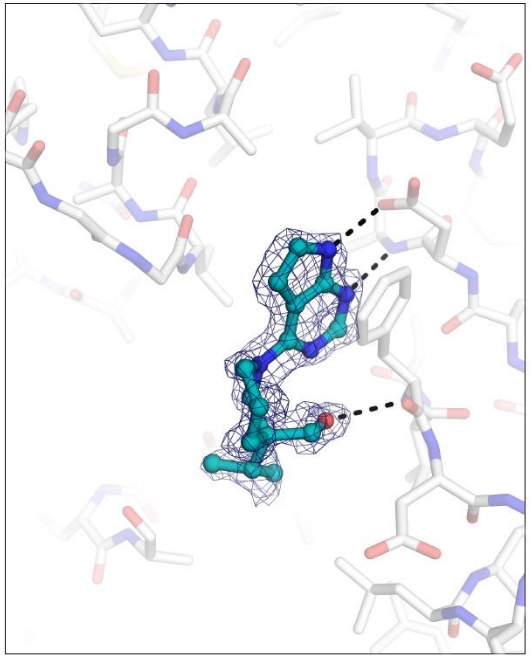

Blue mesh: PanDDA event map contoured at 2 σ

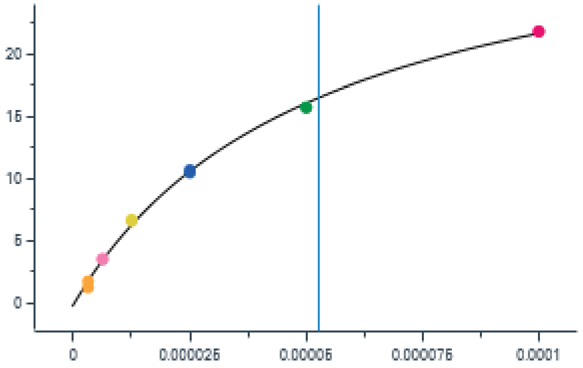

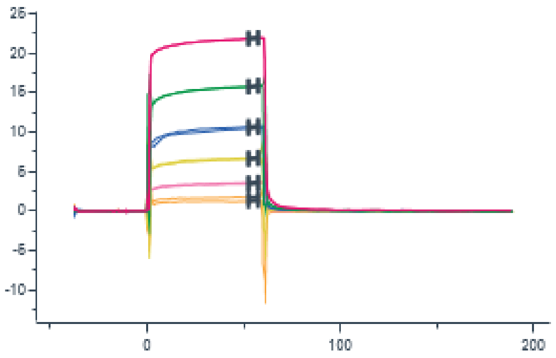

Closest
published hit
dist: 0.1
Ki:439 μM

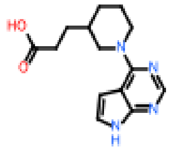

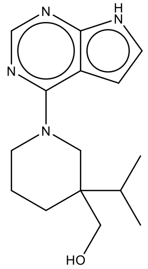

CACHE3HI_1708_87

K_D = 53 μM – 106% binding

Selectivity for NSP3 (against PARP14a protein) – Yes

DLS (solub@100 μM)

HTRF_displacement:

%inh@100 uM = 52

%inh@50 uM = 38

%inh@25 uM = 20

Structure – Yes !

CACHE#3 – SARS-CoV2 Nsp3 macrodomain
Participant 1709

Crystallized molecule that was not advanced to round 2

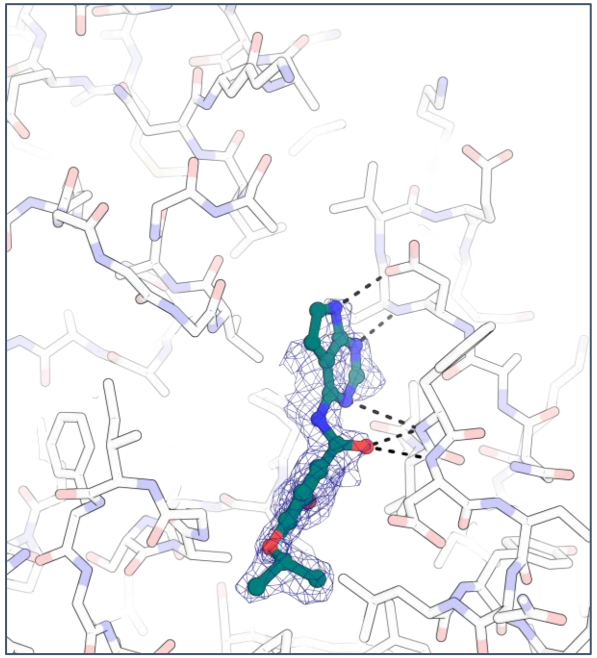

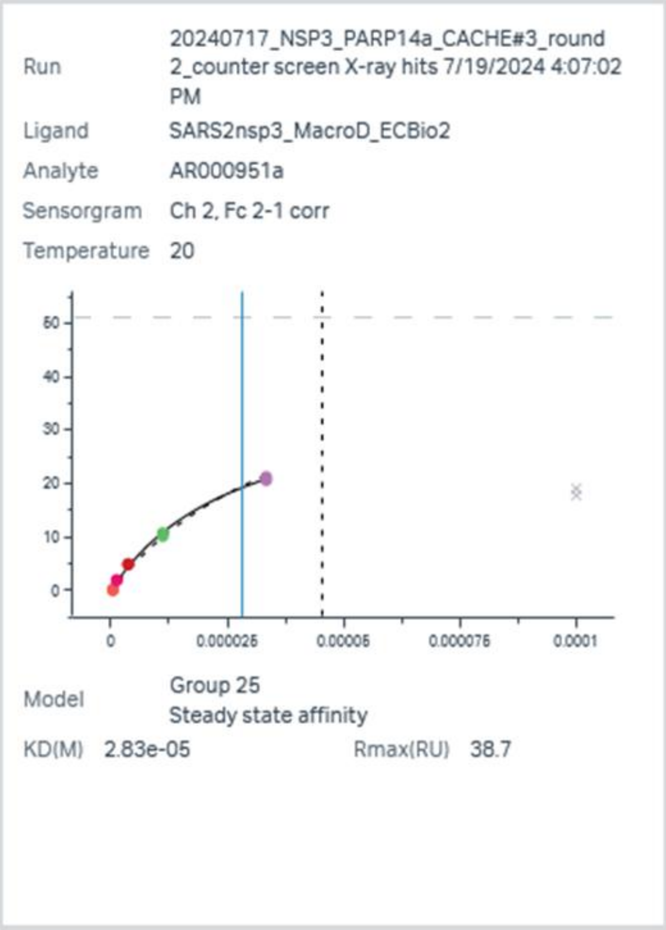

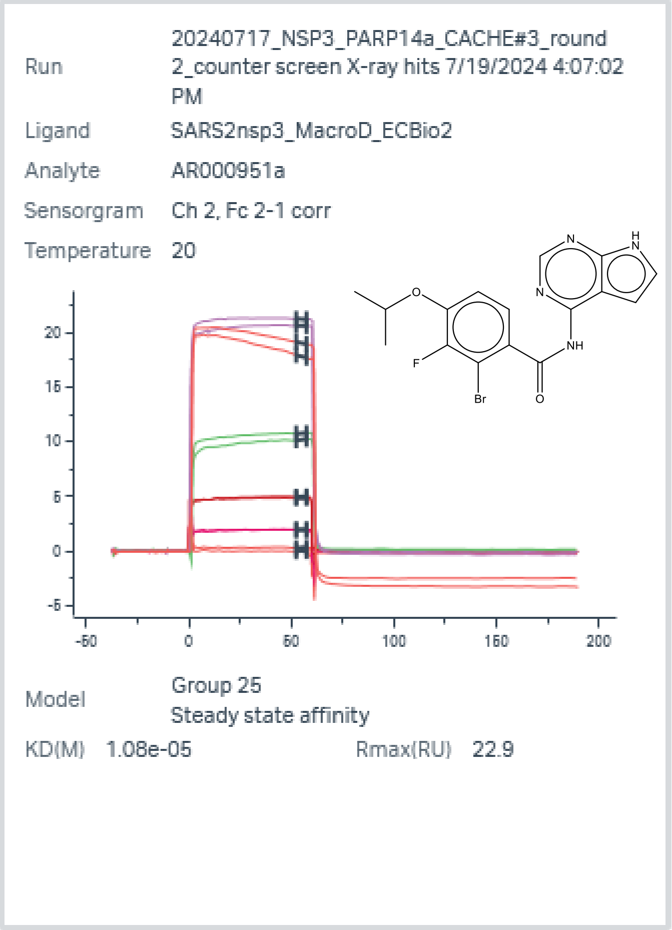

CACHE3HI_1709_75

K_D (4% DMSO) = 28 µM (solubility issues) – 76% binding

K_D (2% DMSO) = N/A

Selectivity for NSP3 (against PARP14a protein) – Yes

Solubility (DLS) – 100 uM

HTRF_displacement hit confirmation (2% DMSO):

%inh@100 uM = 71

%inh@50 uM = 52

%inh@25 uM = 23

Structure – Yes !

Closest published hit
dist: 0.4
Ki:114 µM

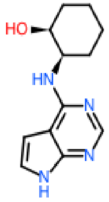

CACHE#3 – SARS-CoV2 Nsp3 macrodomain
Participant 1714

PARENT MOLECULE

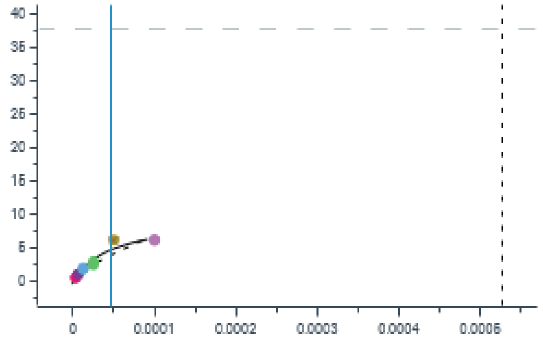

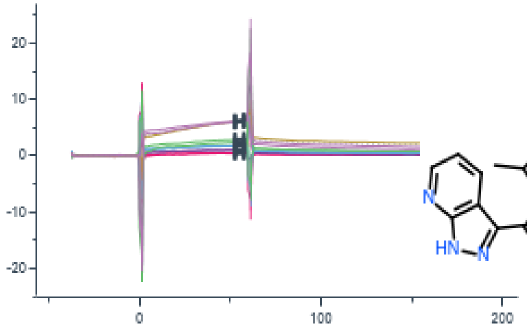

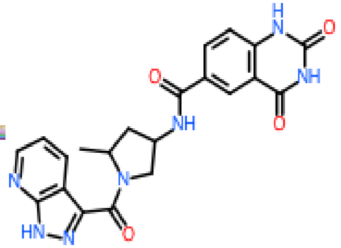

CACHE3HI_1714_34

K_D = 46 µM (low binding) – 25% binding
Selectivity for NSP3 (against PARP14a protein) – 43 µM – 27% binding
DLS (solub@100 µM)
HTRF_displacement:
%inh@100 uM = 40
%inh@50 uM = 15
%inh@25 uM = 1

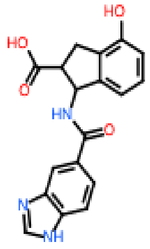

Closest published hit
dist: 0.5
Ki:4.7 µM

27 analogs, including parent molecule, of CACHE3HI_1714_34 chemotype were submitted for round 2.
5 compounds, including a re-supplied parent molecule, showed a dose depended displacement of ADP-ribose peptide by HTRF and did not confirm binding by SPR (less than 10% binding response).

CACHE3-HO_1714_1

K_D = N/A (Low binding) – 1% binding
DLS (solub@100 µM)
HTRF_displacement hit confirmation (4% DMSO):
%inh@100 uM = 78
%inh@30 uM = 3
%inh@10 uM = 10

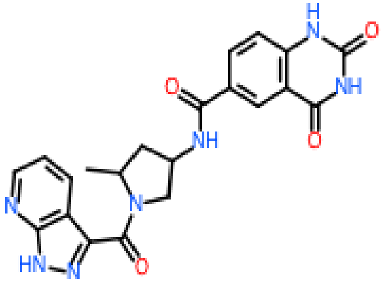

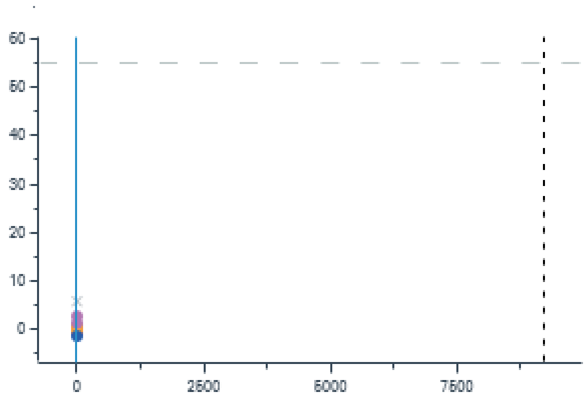

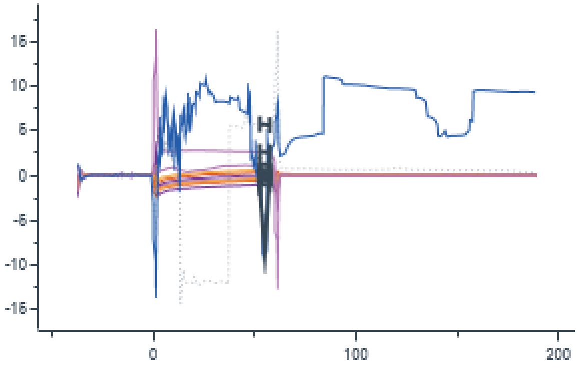

Tested analogs

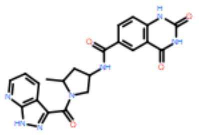
CACHE_ID CACHE3-HO_1714_1
Parent CACHE3HI_1714_34
distance 0

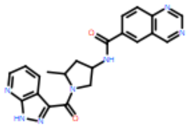
CACHE_ID CACHE3-HO_1714_10
Parent CACHE3HI_1714_34
distance 0.1026

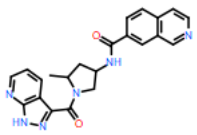
CACHE_ID CACHE3-HO_1714_9
Parent CACHE3HI_1714_34
distance 0.1619

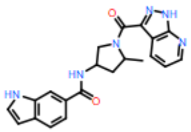
CACHE_ID CACHE3-HO_1714_21
Parent CACHE3HI_1714_34
distance 0.1796

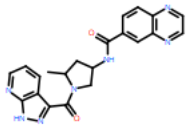
CACHE_ID CACHE3-HO_1714_11
Parent CACHE3HI_1714_34
distance 0.1863

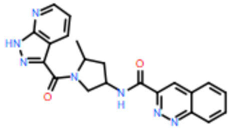
CACHE_ID CACHE3-HO_1714_13
Parent CACHE3HI_1714_34
distance 0.192

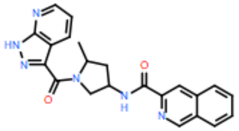
CACHE_ID CACHE3-HO_1714_7
Parent CACHE3HI_1714_34
distance 0.1933

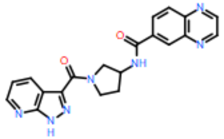
CACHE_ID CACHE3-HO_1714_25
Parent CACHE3HI_1714_34
distance 0.2105

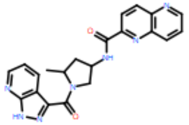
CACHE_ID CACHE3-HO_1714_12
Parent CACHE3HI_1714_34
distance 0.2175

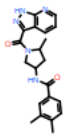
CACHE_ID CACHE3-HO_1714_23
Parent CACHE3HI_1714_34
distance 0.224

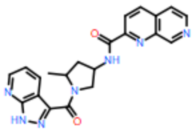
CACHE_ID CACHE3-HO_1714_22
Parent CACHE3HI_1714_34
distance 0.2292

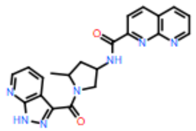
CACHE_ID CACHE3-HO_1714_8
Parent CACHE3HI_1714_34
distance 0.231

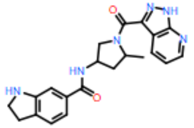
CACHE_ID CACHE3-HO_1714_20
Parent CACHE3HI_1714_34
distance 0.2472

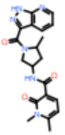
CACHE_ID CACHE3-HO_1714_18
Parent CACHE3HI_1714_34
distance 0.2551

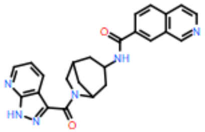
CACHE_ID CACHE3-HO_1714_2
Parent CACHE3HI_1714_34
distance 0.2598

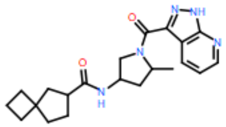
CACHE_ID CACHE3-HO_1714_24
Parent CACHE3HI_1714_34
distance 0.2667

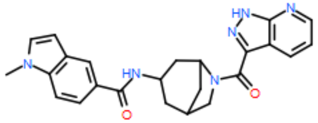
CACHE_ID CACHE3-HO_1714_6
Parent CACHE3HI_1714_34
distance 0.2692

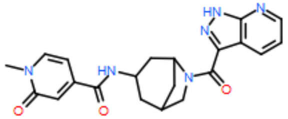
CACHE_ID CACHE3-HO_1714_27
Parent CACHE3HI_1714_34
distance 0.2834

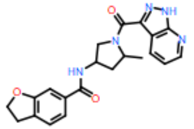
CACHE_ID CACHE3-HO_1714_15
Parent CACHE3HI_1714_34
distance 0.2837

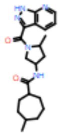
CACHE_ID CACHE3-HO_1714_16
Parent CACHE3HI_1714_34
distance 0.2848

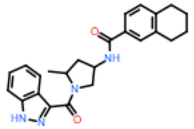
CACHE_ID CACHE3-HO_1714_3
Parent CACHE3HI_1714_34
distance 0.2925

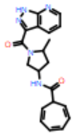
CACHE_ID CACHE3-HO_1714_26
Parent CACHE3HI_1714_34
distance 0.2938

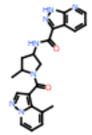
CACHE_ID CACHE3-HO_1714_17
Parent CACHE3HI_1714_34
distance 0.3104

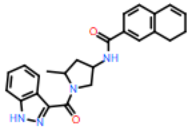
CACHE_ID CACHE3-HO_1714_4
Parent CACHE3HI_1714_34
distance 0.32

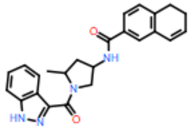
CACHE_ID CACHE3-HO_1714_5
Parent CACHE3HI_1714_34
distance 0.32

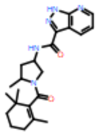
CACHE_ID CACHE3-HO_1714_14
Parent CACHE3HI_1714_34
distance 0.3324

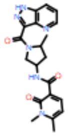
CACHE_ID CACHE3-HO_1714_19
Parent CACHE3HI_1714_34
distance 0.3646

CACHE#3 – SARS-CoV2 Nsp3 macrodomain
Participant 1715

PARENT MOLECULE

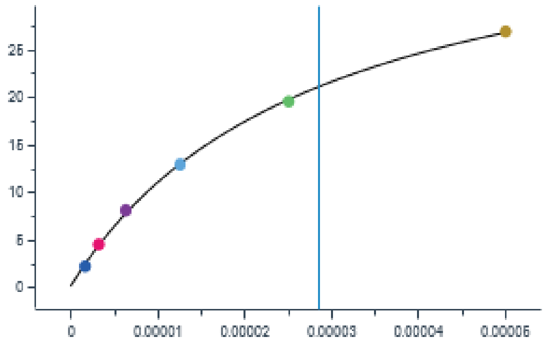

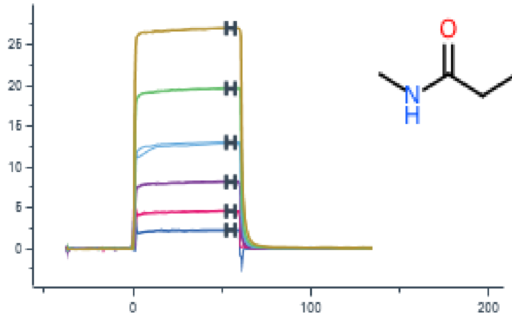

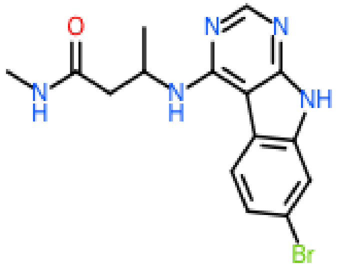

CACHE3HI_1715_71

K_D = 28 µM – 128% binding
Selectivity for NSP3 (against PARP14a protein) – Yes
DLS (solub@50 µM)
HTRF_displacement (2% DMSO):
%inh@100 uM = 66
%inh@50 uM = 93
%inh@25 uM = 23

12 analogs, including parent molecule, of CACHE3HI_1715_71 chemotype were submitted for round 2.
12 compounds, including a re-supplied parent molecule, showed a dose depended displacement of ADP-ribose peptide by HTRF. Among them **6 compounds** confirmed a dose dependent binding response reaching saturation (**1 compounds** does not reach saturation)

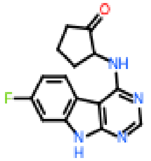

Closest published hit
dist: 0.2
Ki:12 µM

CACHE3-HO_1715_45

K_D = 18 µM – 89% binding
DLS (solub@50 µM)
HTRF_displacement hit confirmation (4% DMSO):
%inh@100 uM = 56
%inh@30 uM = 23
%inh@10 uM = 14

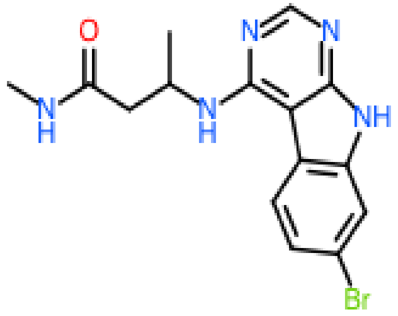

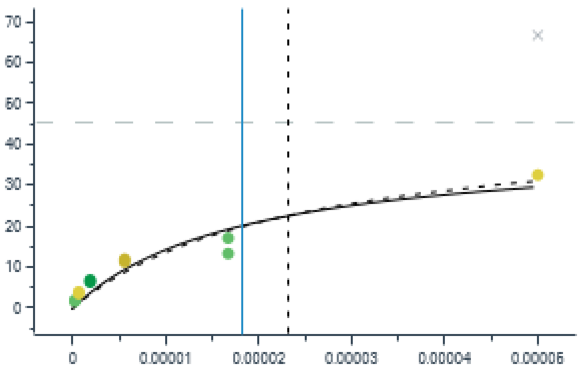

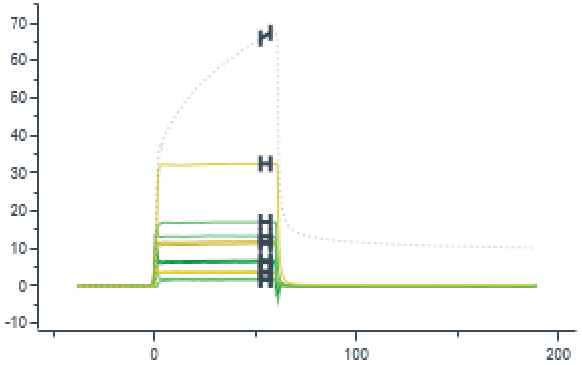

Tested analogs

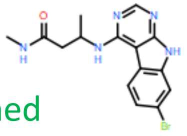<br>confirmed<br>CACHE_ID CACHE3-HO_1715_45<br>Parent CACHE3HI_1715_71<br>distance 0	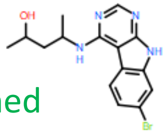<br>confirmed<br>CACHE_ID CACHE3-HO_1715_23<br>Parent CACHE3HI_1715_71<br>distance 0.09677	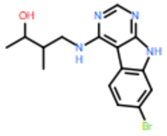<br>CACHE_ID CACHE3-HO_1715_24<br>Parent CACHE3HI_1715_71<br>distance 0.1012	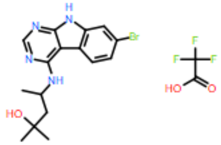<br>CACHE_ID CACHE3-HO_1715_38<br>Parent CACHE3HI_1715_71<br>distance 0.1036	does not reach saturation<br>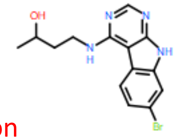<br>CACHE_ID CACHE3-HO_1715_28<br>Parent CACHE3HI_1715_71<br>distance 0.1053
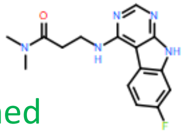<br>confirmed<br>CACHE_ID CACHE3-HO_1715_18<br>Parent CACHE3HI_1715_71<br>distance 0.1071	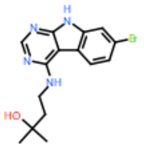<br>CACHE_ID CACHE3-HO_1715_22<br>Parent CACHE3HI_1715_71<br>distance 0.1089	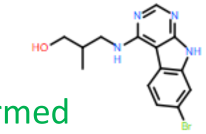<br>confirmed<br>CACHE_ID CACHE3-HO_1715_27<br>Parent CACHE3HI_1715_71<br>distance 0.1138	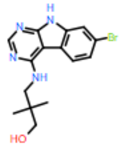<br>CACHE_ID CACHE3-HO_1715_25<br>Parent CACHE3HI_1715_71<br>distance 0.1215	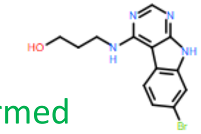<br>confirmed<br>CACHE_ID CACHE3-HO_1715_30<br>Parent CACHE3HI_1715_71<br>distance 0.1301
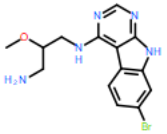<br>CACHE_ID CACHE3-HO_1715_19<br>Parent CACHE3HI_1715_71<br>distance 0.1705	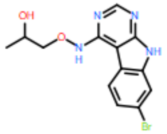<br>CACHE_ID CACHE3-HO_1715_15<br>Parent CACHE3HI_1715_71<br>distance 0.3156			

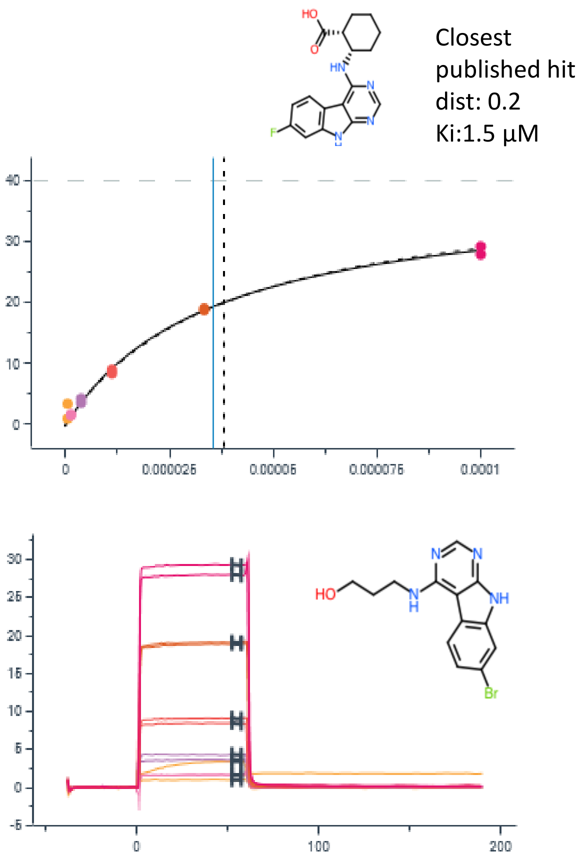

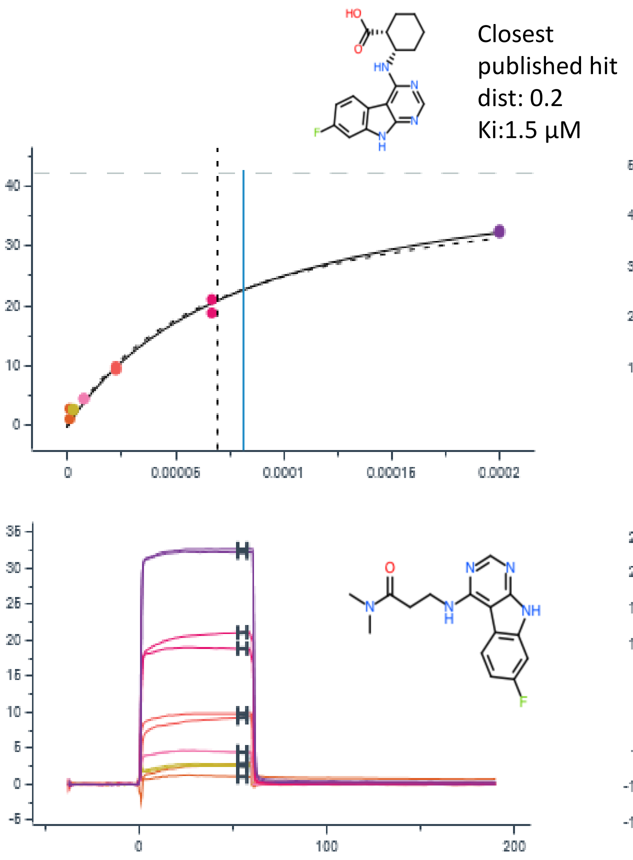

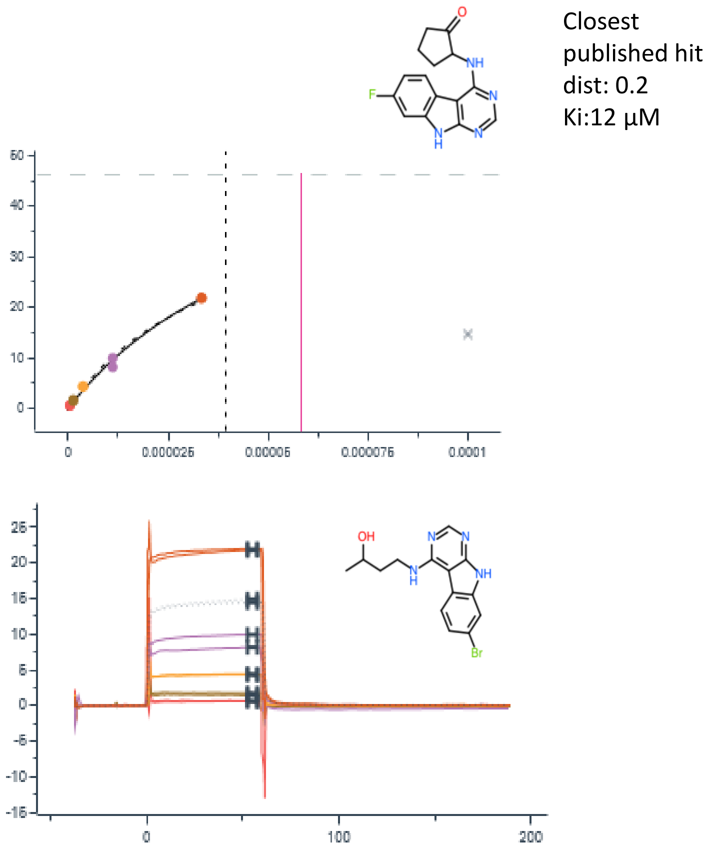

CACHE3-HO_1715_30

K_D (run 1) = 36 µM – 97% binding
K_D (run 2) = 51 µM – 119% binding
Selectivity for NSP3 (against PARP14a protein) – Yes
DLS (solub@100 µM)
HTRF_displacement hit confirmation (4% DMSO):
%inh@100 uM = 46
%inh@30 uM = 16
%inh@10 uM = 12

CACHE3-HO_1715_18

K_D (run 1) = 74 µM – 84% binding
K_D (run 2) = 82 µM – 108% binding
Selectivity for NSP3 (against PARP14a protein) – Yes
DLS (solub@100 µM)
HTRF_displacement hit confirmation (4% DMSO):
%inh@100 uM = 23
%inh@30 uM = 8
%inh@10 uM = 10

CACHE3-HO_1715_28

K_D = 58 µM (does not reach saturation) – 130% binding
Selectivity for NSP3 (against PARP14a protein) – Yes
DLS (solub@100 µM)
HTRF_displacement hit confirmation (4% DMSO):
%inh@100 uM = 82
%inh@30 uM = 19
%inh@10 uM = 15

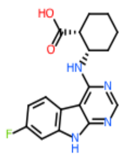

Closest
published hit
dist: 0.2
Ki:1.5 µM

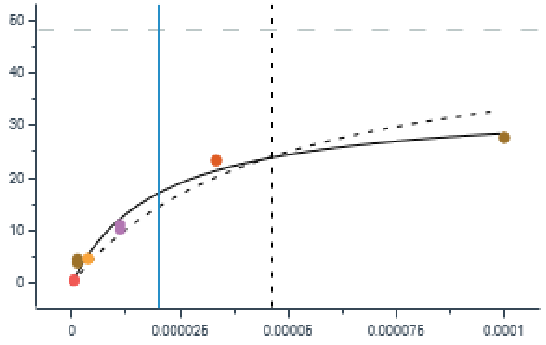

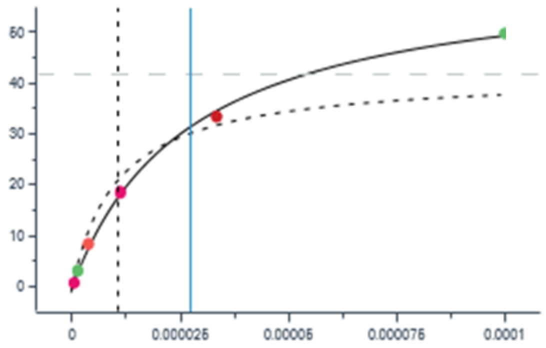

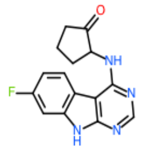

Closest
published hit
dist: 0.2
Ki:12 µM

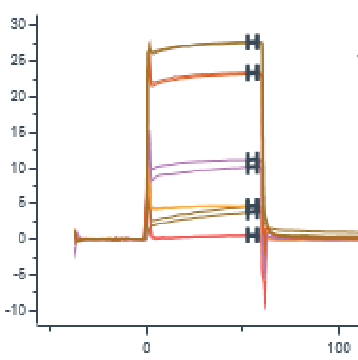

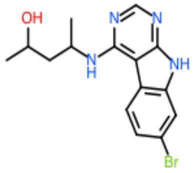

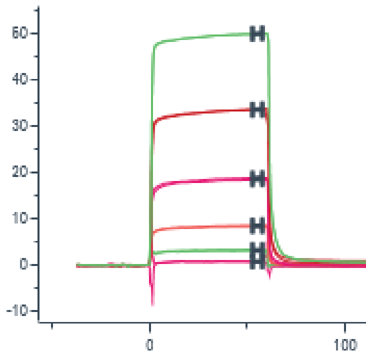

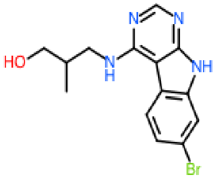

CACHE3-HO_1715_23

K_D = 20 µM – 71% binding
Selectivity for NSP3 (against PARP14a protein) – Yes
DLS (solub@50 µM)
HTRF_displacement hit confirmation (4% DMSO):
%inh@100 uM = 47
%inh@30 uM = 16
%inh@10 uM = 9

CACHE3-HO_1715_27

K_D **(run 1)** = 14 µM – 92% binding
K_D **(run 2)** = 27 µM – 151% binding
Selectivity for NSP3 (against PARP14a protein) – Yes
DLS (solub@50 µM)
HTRF_displacement hit confirmation (4% DMSO):
%inh@100 uM = 66
%inh@30 uM = 27
%inh@10 uM = 17

1 analog of CACHE3HI_1715_76 chemotype were submitted for round 2.
CACHE3-HO_1715_36 showed a mild displacement of ADP-ribose peptide by HTRF and no binding response by SPR.

PARENT MOLECULE

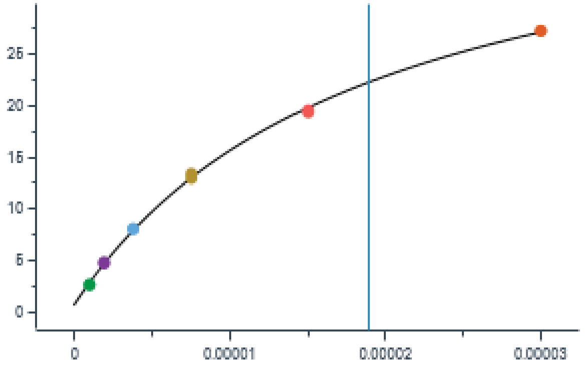

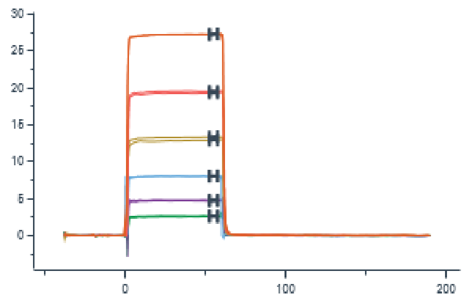

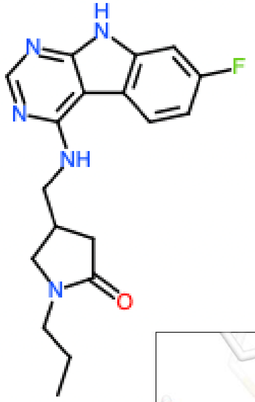

CACHE3HI_1715_76

K_D = 19 µM – 103% binding
Selectivity for NSP3 (against PARP14a
DLS (solub@50 µM)
HTRF_displacement (2% DMSO):
%inh@100 uM = 48
%inh@50 uM = 37
%inh@25 uM = 24

Structure – Yes !

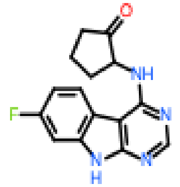

Closest published hit
dist: 0.1
Ki:12 µM

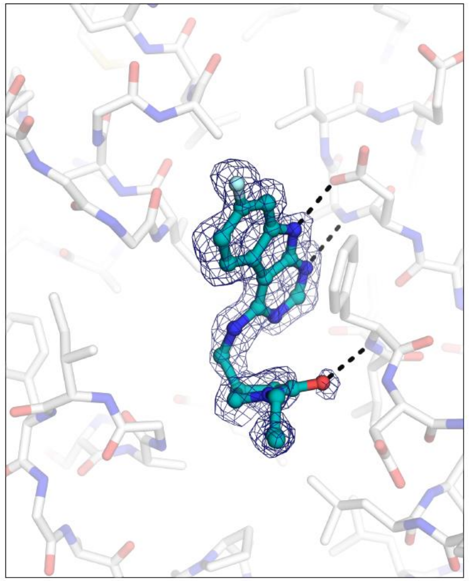

Blue mesh: PanDDA event map contoured at 2 σ

CACHE3-HO_1715_36

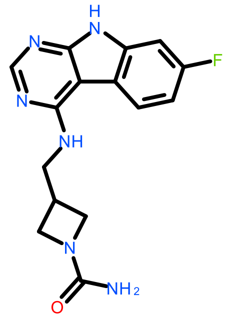

K_D = N/A µM – N/A% binding (low binding, does not reach saturation)
DLS (solub@100 µM)
HTRF_displacement hit confirmation (4% DMSO):
%inh@100 uM = 20
%inh@30 uM = 7
%inh@10 uM = 3

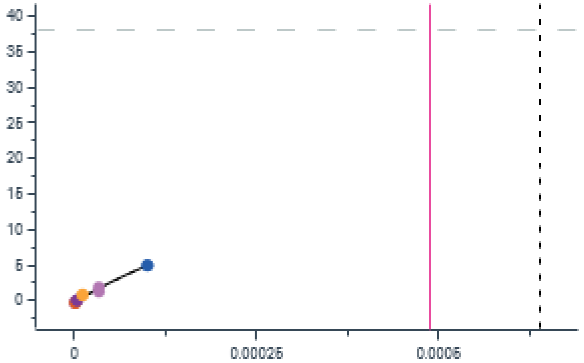

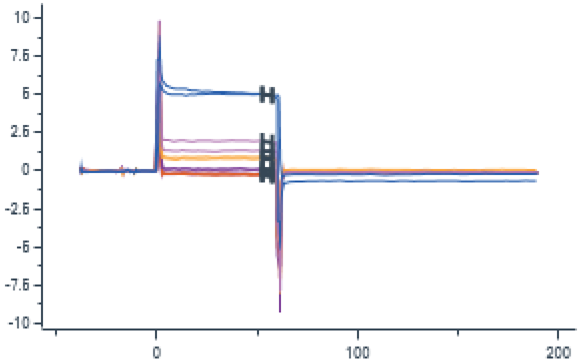

Crystallized molecule that was not advanced to round 2

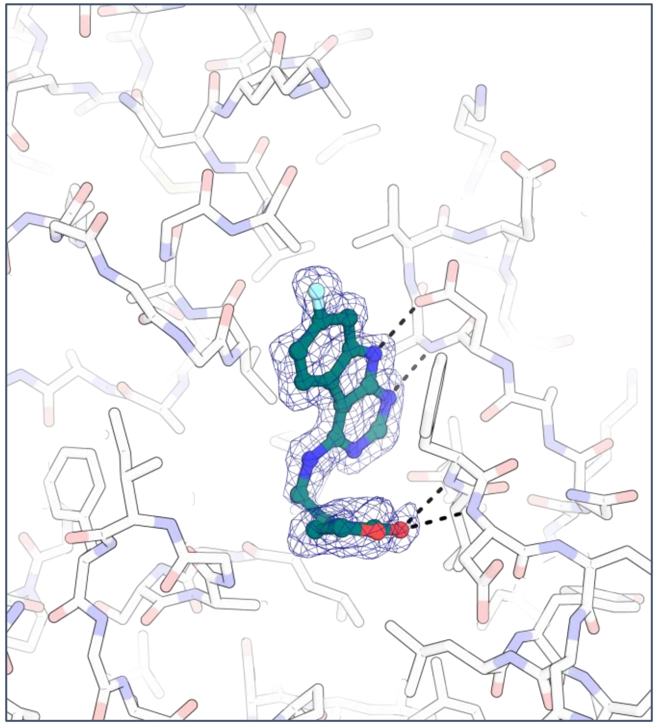

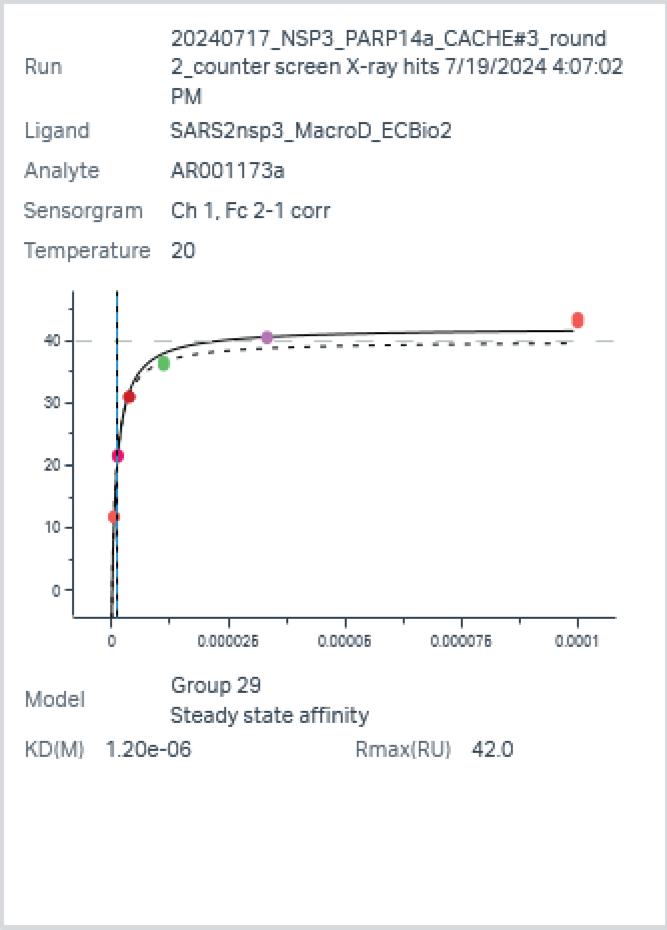

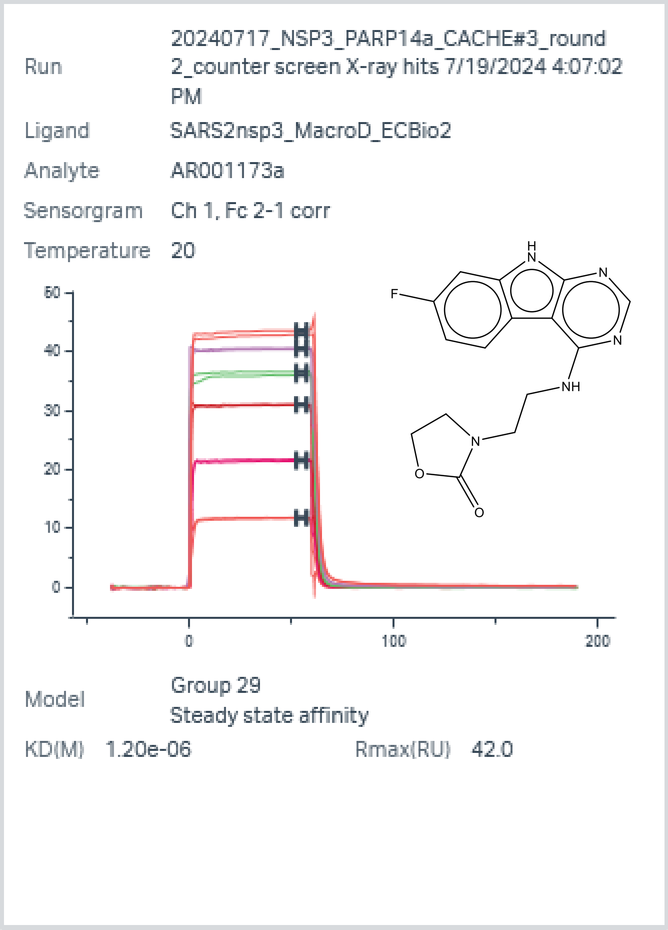

CACHE3HI_1715_78

K_D = 1 µM – 105% binding

Selectivity for NSP3 (against PARP14a protein) – Yes

HTRF_displacement hit confirmation (2% DMSO):

%inh@100 uM = NA

%inh@50 uM = 47

%inh@25 uM = 25

-showed fluorescence interference

Structure – Yes !

Closest published hit
dist: 0.3
Ki:12 µM

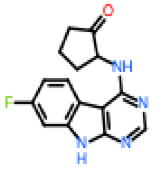

PARENT MOLECULE

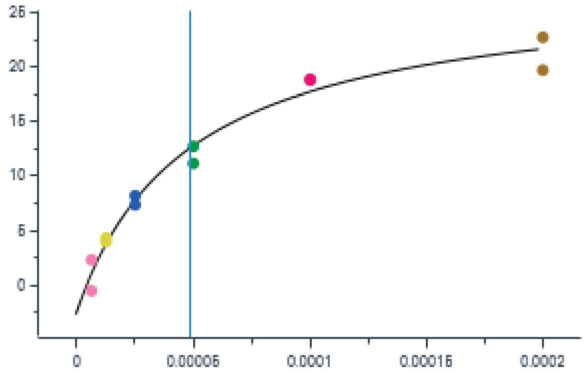

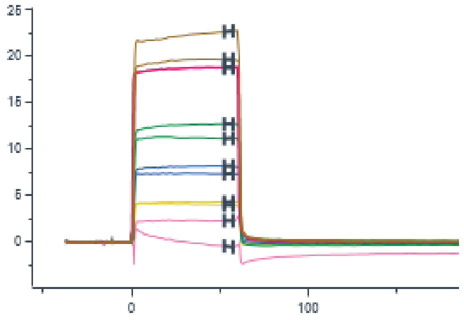

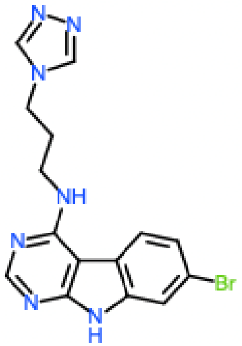

CACHE3HI_1715_82

K_D = 48 µM – 64% binding
Selectivity for NSP3 (against PARP14a protein) – weak binding
DLS (solub@100 µM)
HTRF_displacement (2% DMSO):
%inh@100 uM = 68
%inh@50 uM = 25
%inh@25 uM = 9

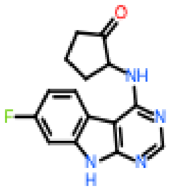

Closest published hit
dist: 0.3
Ki:12 µM

1 analog of CACHE3HI_1715_82 chemotype were submitted for round 2.
CACHE3-HO_1715_41 showed a mild displacement of ADP-ribose peptide by HTRF and low binding response by SPR.

CACHE3-HO_1715_41

K_D =47 µM– Low binding (30%)
DLS (solub@100 µM)
HTRF_displacement hit confirmation (4% DMSO):
%inh@100 uM = 45
%inh@30 uM = 3
%inh@10 uM = 2

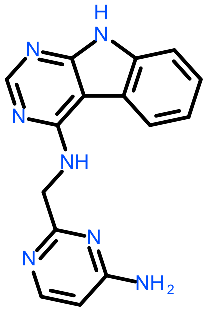

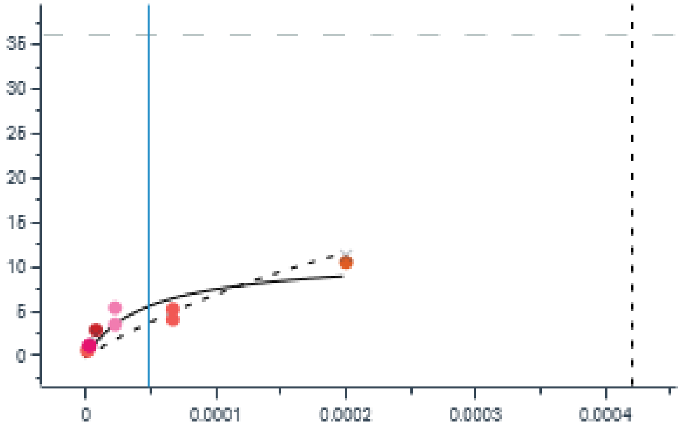

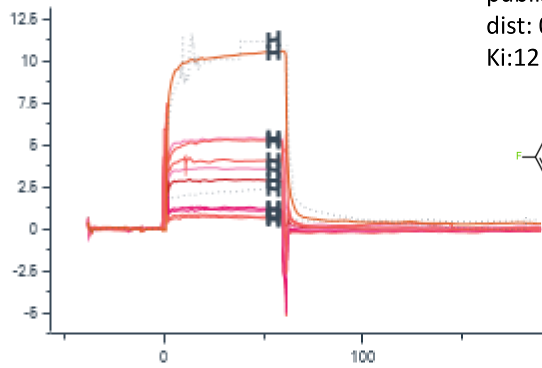

Closest published hit
dist: 0.3
Ki:12 µM

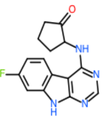

2 analogs of CACHE3HI_1715_83 chemotype were submitted for round 2.

2 compounds showed a mild displacement of ADP-ribose peptide by HTRF. Among them **1 compound** confirmed a dose dependent binding affinity reaching saturation by SPR.

PARENT MOLECULE

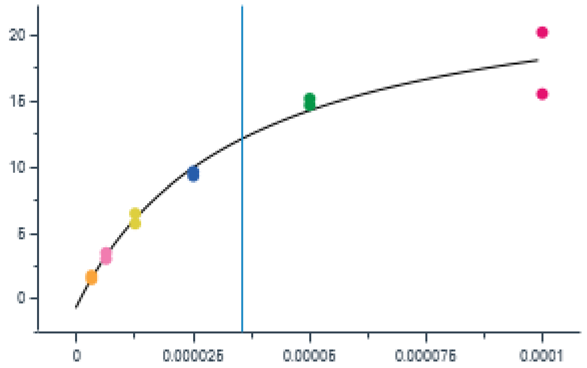

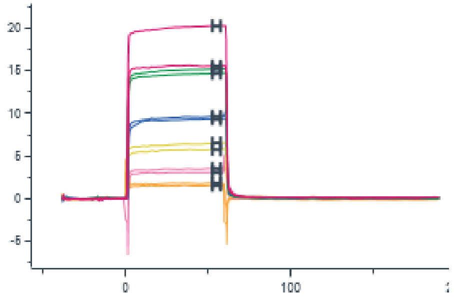

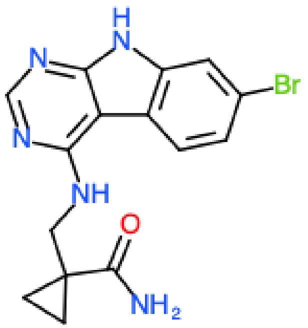

CACHE3HI_1715_83

K_D = 35 μM – 61% binding

Selectivity for NSP3 (against PARP14a protein) – weak binding

DLS (solub@100 μM)

HTRF_displacement (2% DMSO):

%inh@100 uM = 82

%inh@50 uM = 37

%inh@25 uM = 15

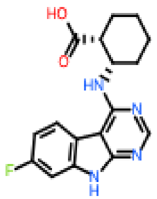

Closest published hit
dist: 0.15
Ki:1.5 μM

CACHE3-HO_1715_40

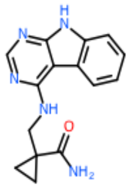

K_D = 20 μM – 97% binding

Selectivity for NSP3 (against PARP14a protein) weak binding

DLS (solub@100 μM)

HTRF_displacement hit confirmation (4% DMSO):

%inh@100 uM = 35

%inh@30 uM = 10

%inh@10 uM = 7

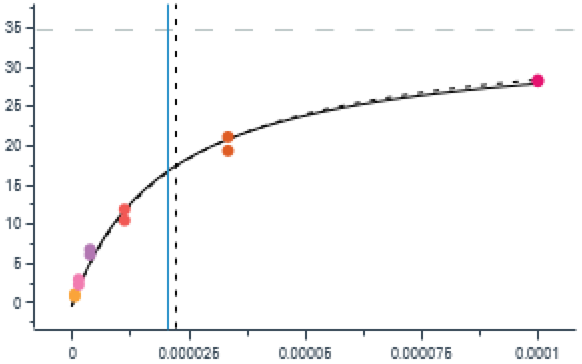

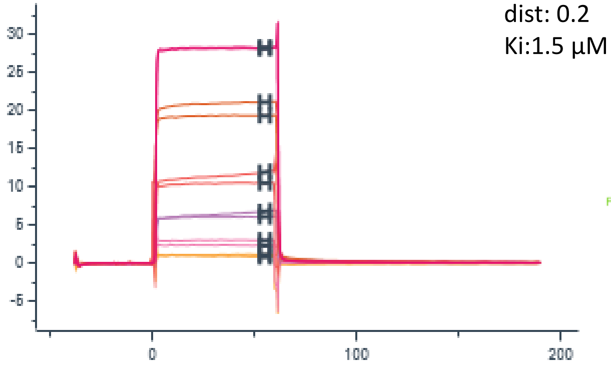

Closest published hit
dist: 0.2
Ki:1.5 μM

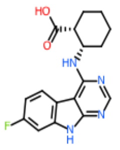

Tested analogs

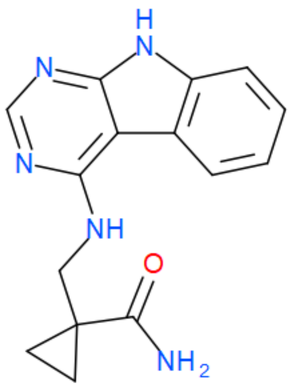

confirmed

CACHE_ID	CACHE3-HO_1715_40
Parent	CACHE3HI_1715_83
distance	0.05578

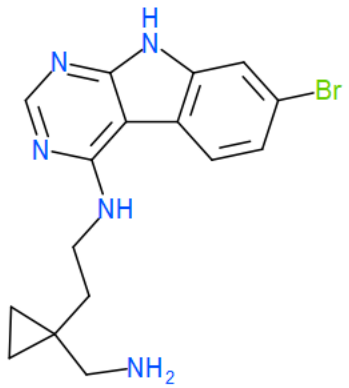

CACHE_ID	CACHE3-HO_1715_37
Parent	CACHE3HI_1715_83
distance	0.112

HIT MOLECULE that was not followed up for round 2

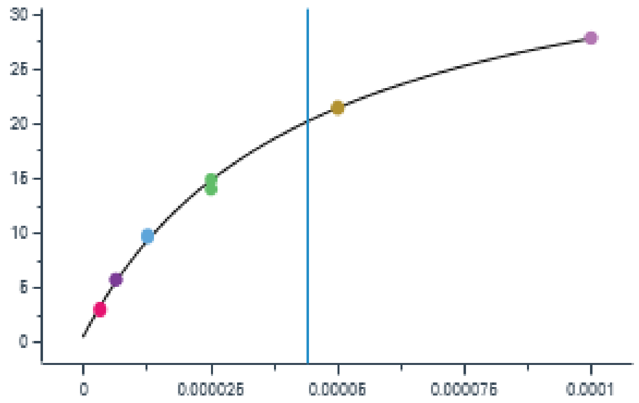

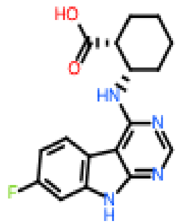

Closest
published hit
dist: 0.1
Ki:1.5 µM

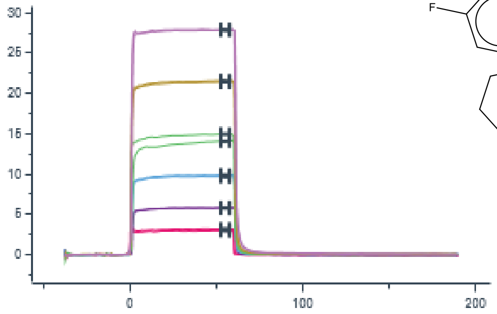

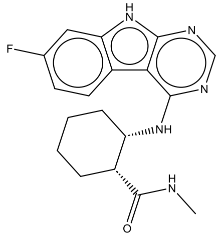

CACHE3HI_1715_85

K_D (run 1) = 44 µM – 124% binding
Selectivity for NSP3 (against PARP14a protein) – Yes
DLS (solub@100 µM)
HTRF_displacement:
%inh@100 uM = 67
%inh@50 uM = 47
%inh@25 uM = 32

Structure – Yes !

PARENT MOLECULE

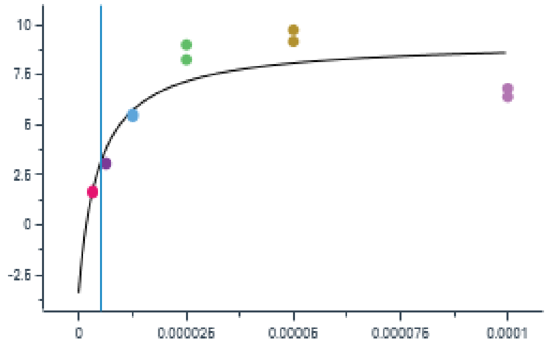

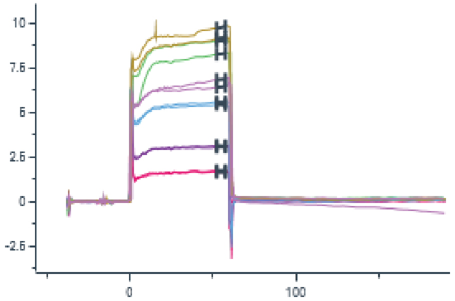

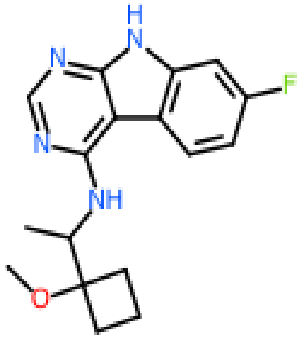

CACHE3HI_1715_88

K_D = 49 µM (poor fit) – 35% binding

Selectivity for NSP3 (against PARP14a protein) – Yes

DLS (solub@50 µM)

HTRF_displacement (2% DMSO):

%inh@100 uM = 80

%inh@50 uM = 65

%inh@25 uM = 22

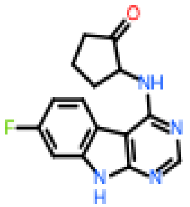

Closest published hit
dist: 0.15
Ki:12 µM

12 analogs, including parent molecule, of CACHE3HI_1715_88 chemotype were submitted for round 2.
6 compounds, including a re-supplied parent molecule, showed a dose depended displacement of ADP-ribose peptide by HTRF and **2 compounds** confirmed a dose depended binding affinity by SPR (**1 compound** did not reach saturation).

CACHE3-HO_1715_46

K_D = 1.4 µM (low binding/poor fit) – 19% binding

DLS (solub@100 µM)

HTRF_displacement hit confirmation (4% DMSO):

%inh@100 uM = 82

%inh@30 uM = 32

%inh@10 uM = 17

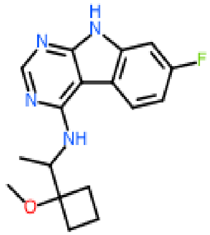

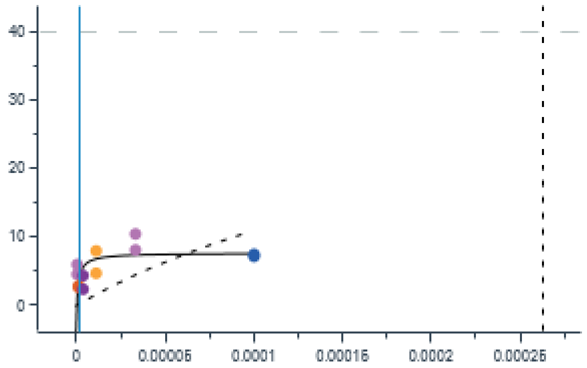

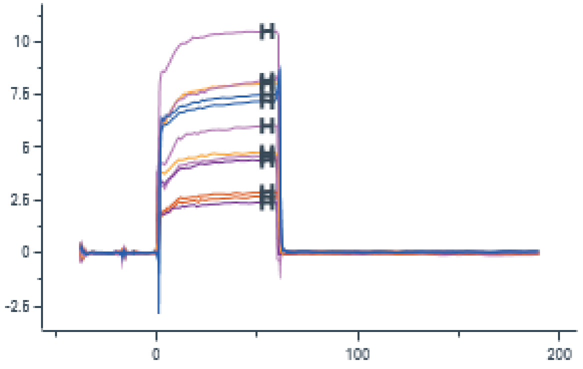

Tested analogs

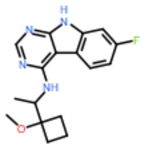<br>CACHE_ID CACHE3-HO_1715_46<br>Parent CACHE3HI_1715_88<br>distance 0	does not reach saturation 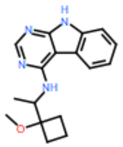<br>CACHE_ID CACHE3-HO_1715_2<br>Parent CACHE3HI_1715_88<br>distance 0.04724	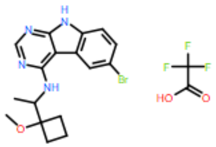<br>CACHE_ID CACHE3-HO_1715_5<br>Parent CACHE3HI_1715_88<br>distance 0.05323	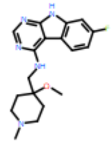<br>CACHE_ID CACHE3-HO_1715_12<br>Parent CACHE3HI_1715_88<br>distance 0.0989	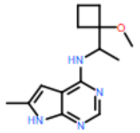<br>CACHE_ID CACHE3-HO_1715_6<br>Parent CACHE3HI_1715_88<br>distance 0.3511
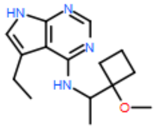<br>CACHE_ID CACHE3-HO_1715_1<br>Parent CACHE3HI_1715_88<br>distance 0.372	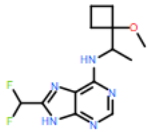<br>CACHE_ID CACHE3-HO_1715_11<br>Parent CACHE3HI_1715_88<br>distance 0.4498	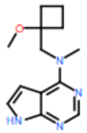<br>CACHE_ID CACHE3-HO_1715_9<br>Parent CACHE3HI_1715_88<br>distance 0.4696	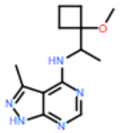<br>CACHE_ID CACHE3-HO_1715_3<br>Parent CACHE3HI_1715_88<br>distance 0.4721	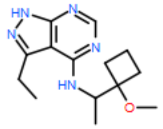<br>CACHE_ID CACHE3-HO_1715_10<br>Parent CACHE3HI_1715_88<br>distance 0.4826
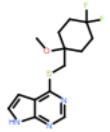 confirmed<br>CACHE_ID CACHE3-HO_1715_7<br>Parent CACHE3HI_1715_88<br>distance 0.5666	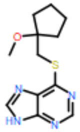<br>CACHE_ID CACHE3-HO_1715_8<br>Parent CACHE3HI_1715_88<br>distance 0.6269			

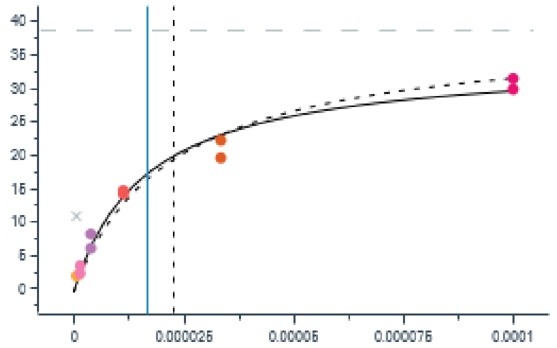

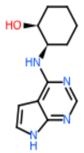

Closest published hit
dist: 0.5
Ki:114 µM

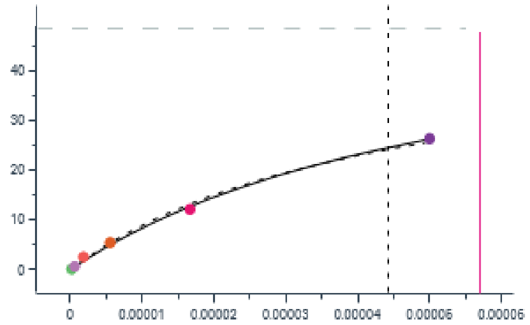

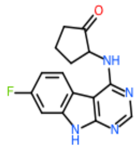

Closest published hit
dist: 0.2
Ki:12 µM

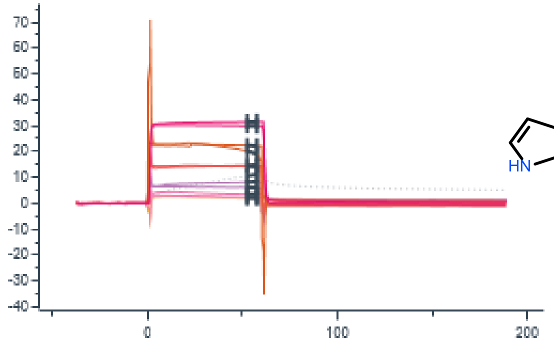

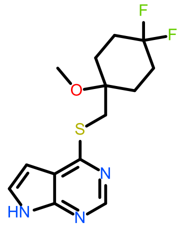

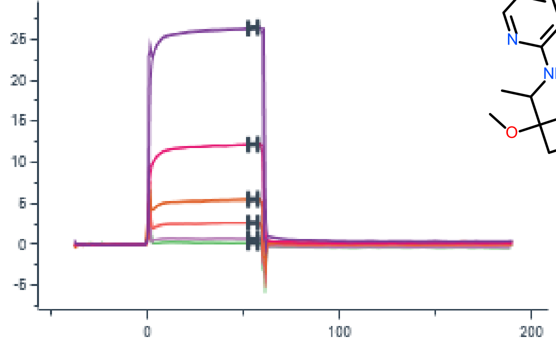

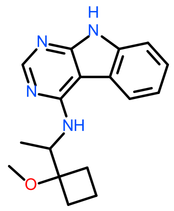

CACHE3-HO_1715_7

K_D = 17 µM – 89% binding
DLS (solub@100 µM)
HTRF_displacement hit confirmation (4% DMSO):
%inh@100 uM = 17
%inh@30 uM = 7
%inh@10 uM = 0

CACHE3-HO_1715_2

K_D = 57 µM (does not reach saturation) – 116% binding
Selectivity for NSP3 (against PARP14a protein) – Yes
DLS (solub@50 µM)
HTRF_displacement hit confirmation (4% DMSO):
%inh@100 uM = 79
%inh@30 uM = 34
%inh@10 uM = 15

PARENT MOLECULE

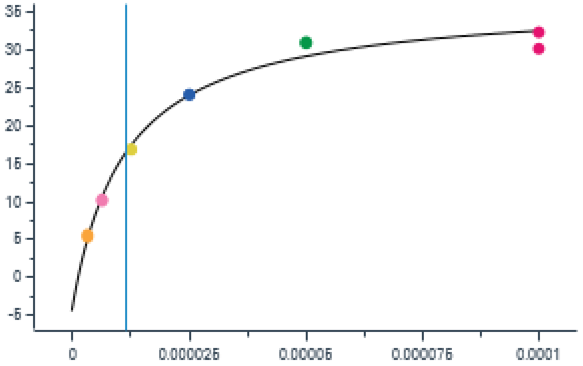

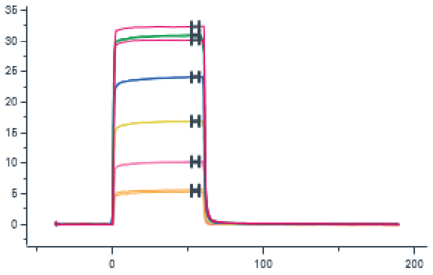

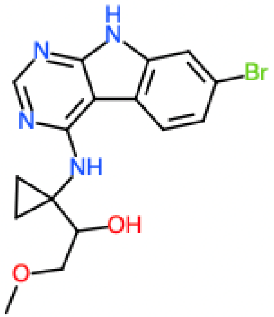

CACHE3HI_1715_89

K_D = 12 µM – 87% binding

Selectivity for NSP3 (against PARP14a protein) – Yes

DLS (solub@100 µM)

HTRF_displacement (2% DMSO):

%inh@100 uM = 82

%inh@50 uM = 48

%inh@25 uM = 31

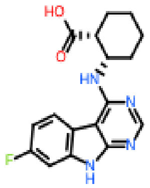

Closest published hit
dist: 0.2
Ki:1.5 µM

3 analogs, including parent molecule, of CACHE3HI_1715_89 chemotype were submitted for round 2.
3 compounds, including a re-supplied parent molecule, showed a dose depended displacement of ADP-ribose peptide by HTRF and confirmed binding affinity by SPR.

CACHE3-HO_1715_47

K_D = 14 µM (poor fit) – 69% binding

DLS (solub@100 µM)

HTRF_displacement hit confirmation (4% DMSO):

%inh@100 uM = 55

%inh@30 uM = 19

%inh@10 uM = 15

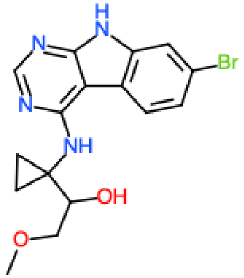

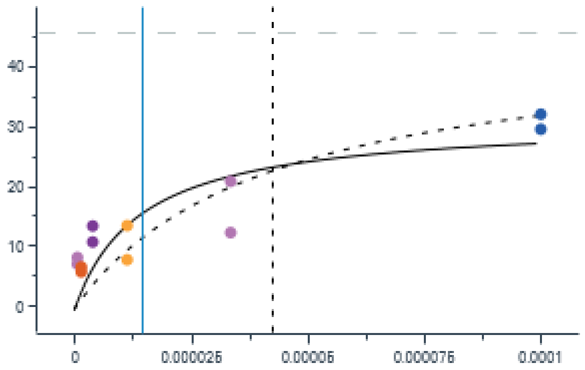

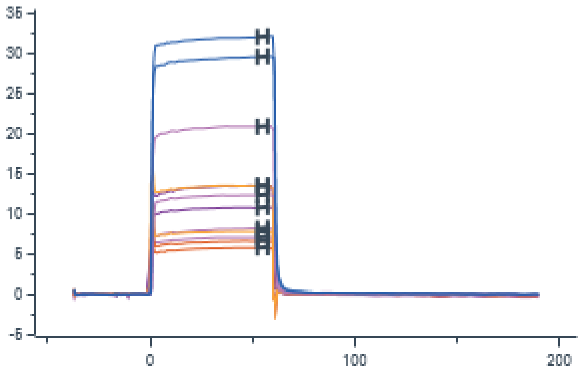

Tested analogs

confirmed

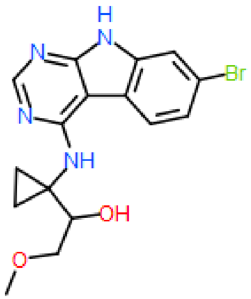

CACHE_ID CACHE3-HO_1715_47
Parent CACHE3HI_1715_89
distance 0

confirmed

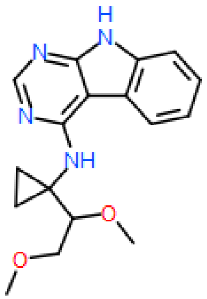

CACHE_ID CACHE3-HO_1715_16
Parent CACHE3HI_1715_89
distance 0.1095

confirmed

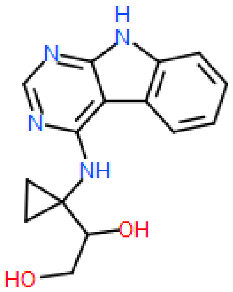

CACHE_ID CACHE3-HO_1715_29
Parent CACHE3HI_1715_89
distance 0.117

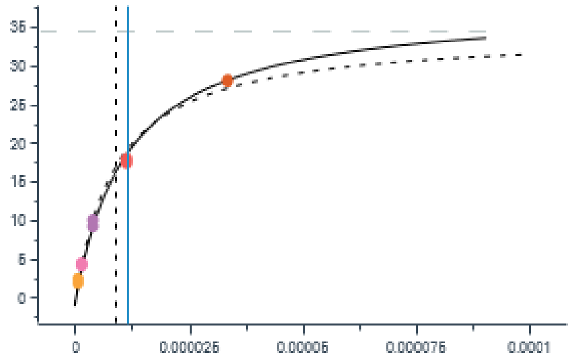

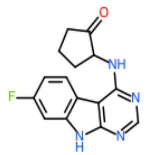

Closest published hit
dist: 0.2
Ki:12 µM

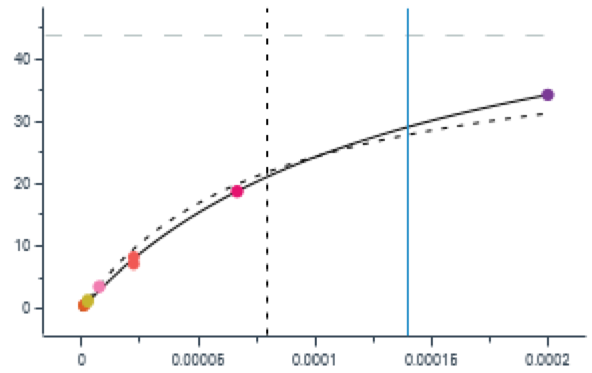

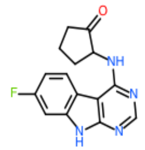

Closest published hit
dist: 0.2
Ki:12 µM

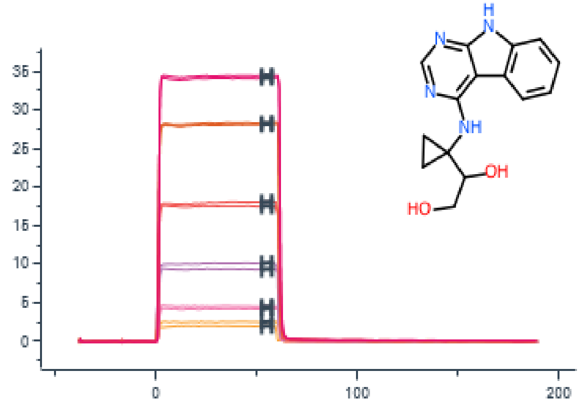

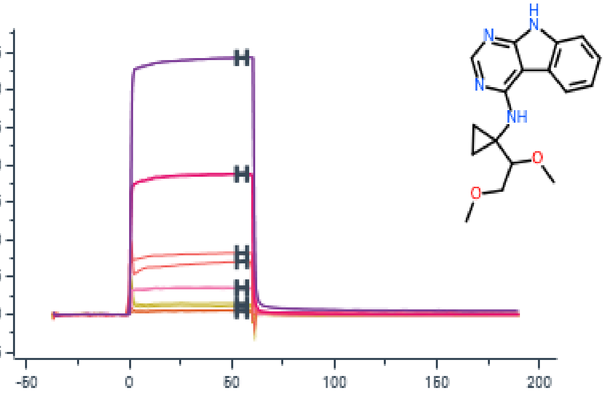

CACHE3-HO_1715_29

K_D (run 1) = 12 µM – 111% binding
K_D (run 2) = 18 µM – 118% binding
Selectivity for NSP3 (against PARP14a protein) – Yes
DLS (solub@100 µM)
HTRF_displacement hit confirmation (4% DMSO):
%inh@100 uM = 65
%inh@30 uM = 31
%inh@10 uM = 15

CACHE3-HO_1715_16

K_D = 140 µM – 133% binding
Selectivity for NSP3 (against PARP14a protein) – weak binding (slow on/off)
DLS (solub@100 µM)
HTRF_displacement hit confirmation (4% DMSO):
%inh@100 uM = 19
%inh@30 uM = 6
%inh@10 uM = 0

PARENT MOLECULE

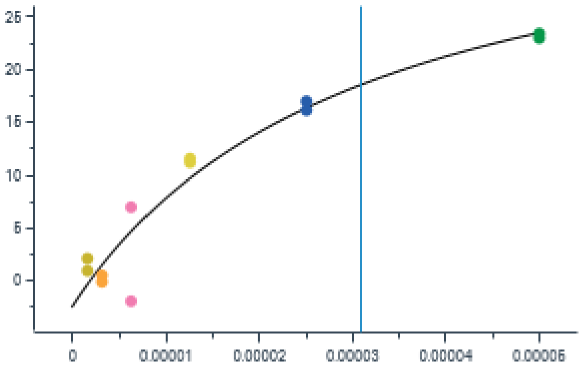

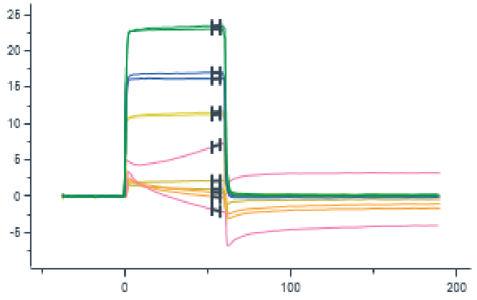

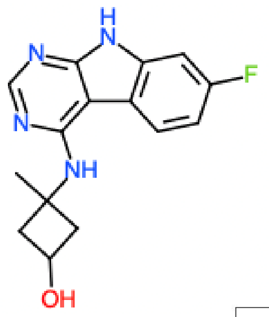

CACHE3HI_1715_91

K_D = 31 µM – 124% binding

Selectivity for NSP3 (against PARP14a protein) – Yes

DLS (solub@100 µM)

HTRF_displacement (2% DMSO):

%inh@100 uM = 57

%inh@50 uM = 40

%inh@25 uM = 23

Structure – Yes !

9 analogs of CACHE3HI_1715_91 chemotype were submitted for round 2. **7 compounds** showed a dose depended displacement of ADP-ribose peptide by HTRF. **3 compounds** showed a dose dependent binding response by SPR (**1 compound** may reach saturation at higher tested top concentration)

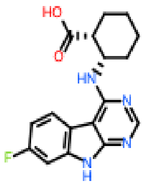

Closest published hit
dist: 0.1
Ki:1.5 µM

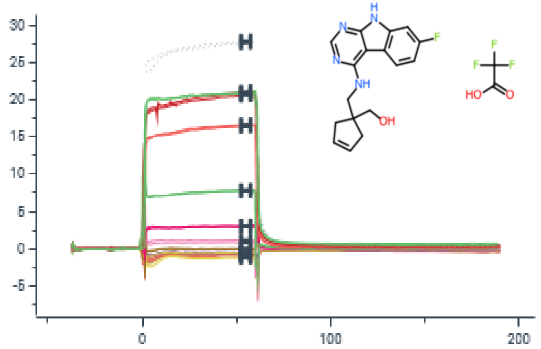

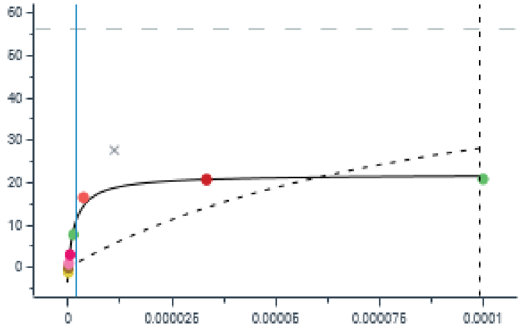

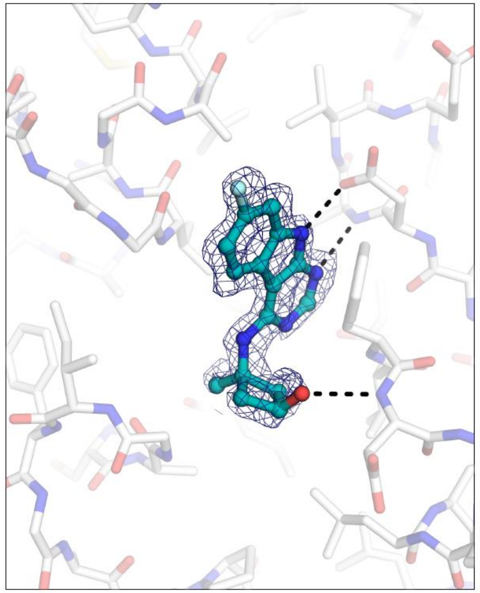

Blue mesh: PanDDA event map contoured at 2 σ

CACHE3-HO_1715_20

K_D = 2 µM –39% binding

Selectivity for NSP3 (against PARP14a protein) – Yes

DLS (solub@100 µM)

HTRF_displacement hit confirmation (4% DMSO):

%inh@100 uM = 80

%inh@30 uM = 52

%inh@10 uM = 40

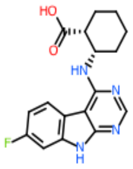

Closest published hit
dist: 0.1
Ki:1.5 µM

Tested analogs

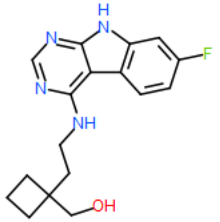

CACHE_ID CACHE3-HO_1715_13
Parent CACHE3HI_1715_91
distance 0.06478

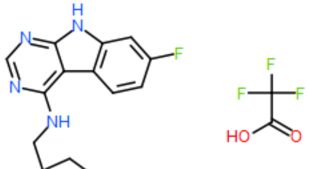

confirmed

CACHE_ID CACHE3-HO_1715_20
Parent CACHE3HI_1715_91
distance 0.08235

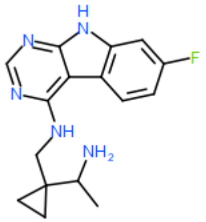

CACHE_ID CACHE3-HO_1715_21
Parent CACHE3HI_1715_91
distance 0.1036

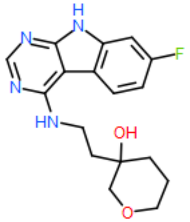

CACHE_ID CACHE3-HO_1715_17
Parent CACHE3HI_1715_91
distance 0.1212

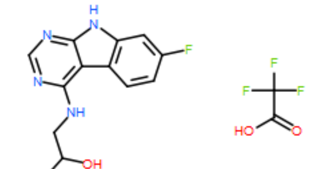

confirmed

CACHE_ID CACHE3-HO_1715_42
Parent CACHE3HI_1715_91
distance 0.261

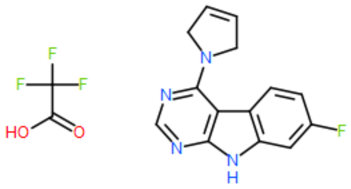

CACHE_ID CACHE3-HO_1715_35
Parent CACHE3HI_1715_91
distance 0.2657

does not reach saturation

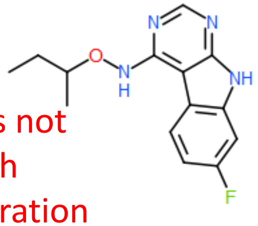

CACHE_ID CACHE3-HO_1715_33
Parent CACHE3HI_1715_91
distance 0.2909

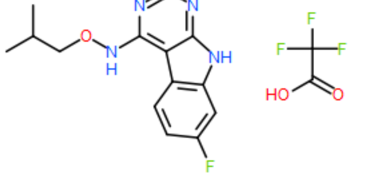

CACHE_ID CACHE3-HO_1715_34
Parent CACHE3HI_1715_91
distance 0.3018

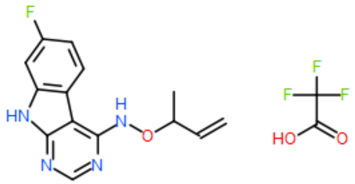

CACHE_ID CACHE3-HO_1715_32
Parent CACHE3HI_1715_91
distance 0.3322

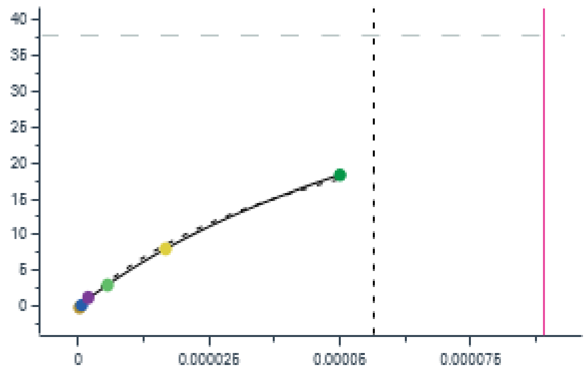

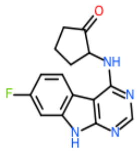

Closest
published hit
dist: 0.3
Ki:12 µM

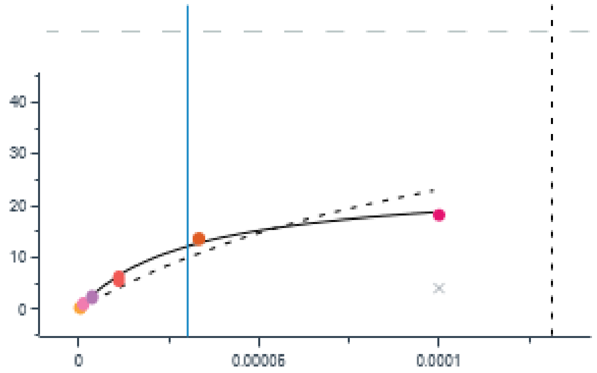

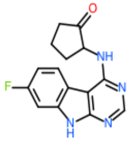

Closest
published hit
dist: 0.2
Ki:12 µM

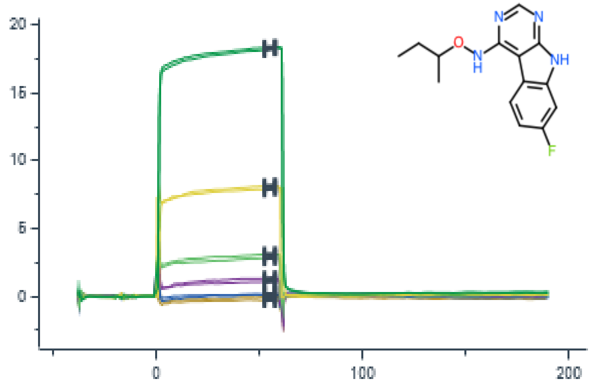

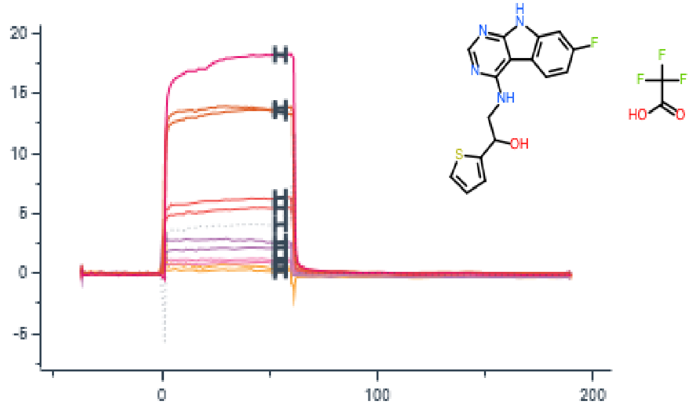

CACHE3-HO_1715_33

K_D = 89 µM (does not reach saturation) – 134% binding

Selectivity for NSP3 (against PARP14a protein) – Yes

DLS (solub@ 30 µM)

HTRF_displacement hit confirmation (4% DMSO):

%inh@100 uM = 44

%inh@30 uM = 20

%inh@10 uM = 1

CACHE3-HO_1715_42

K_D (run 1) = 31 µM – 46% binding

K_D (run 2) = 7 µM (low response) – 15% binding

Selectivity for NSP3 (against PARP14a protein) – 5% binding

DLS (solub@100 µM)

HTRF_displacement hit confirmation (4% DMSO):

%inh@100 uM = 50

%inh@30 uM = 8

%inh@10 uM = 7

PARENT MOLECULE

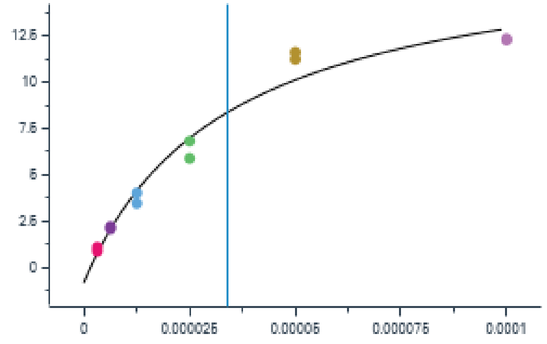

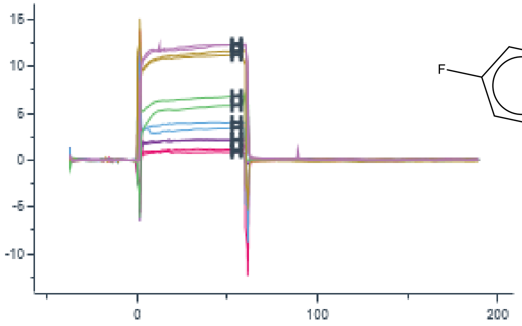

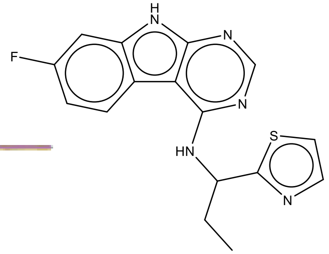

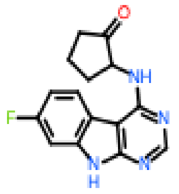

Closest published hit
dist: 0.3
Ki:12 µM

CACHE3HI_1715_92

K_D = 34 µM – 64% binding

Selectivity for NSP3 (against PARP14a protein) – 31% binding

DLS (solub@100 µM)

HTRF_displacement (2% DMSO):

%inh@100 uM = 47

%inh@50 uM = 27

%inh@25 uM = 16

Structure – Yes !

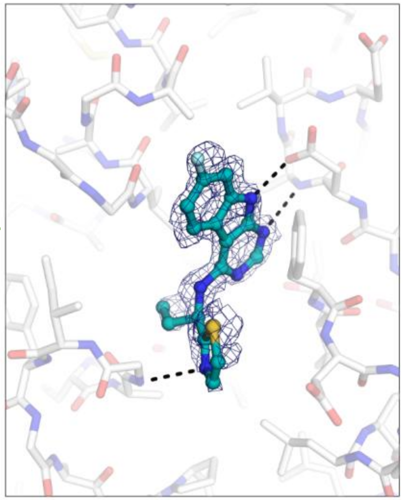

Blue mesh: PanDDA event map contoured at 2 σ

2 analogs of CACHE3HI_1715_92 chemotype were submitted for round 2.

1 compound showed a dose depended displacement of ADP-ribose peptide by HTRF and binding affinity by SPR.

CACHE3-HO_1715_39

K_D = 10 µM – 89% binding

DLS (solub@100 µM)

HTRF_displacement hit confirmation (4% DMSO)

%inh@100 uM = 45

%inh@30 uM = 17

%inh@10 uM = 13

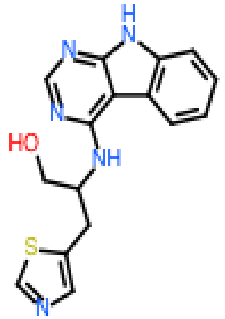

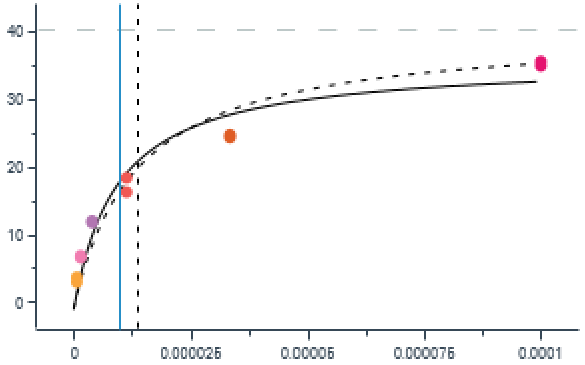

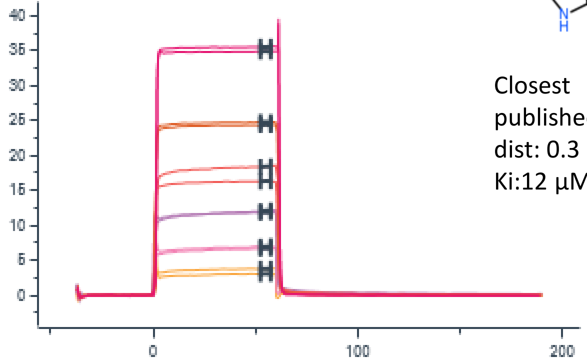

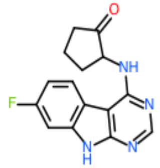

Closest published hit
dist: 0.3
Ki:12 µM

Tested analogs

confirmed

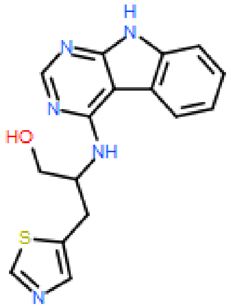

CACHE_ID CACHE3-HO_1715_39
Parent CACHE3HI_1715_92
distance 0.224

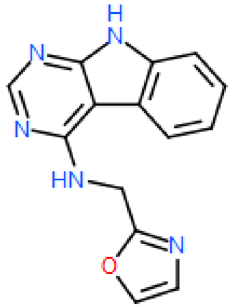

CACHE_ID CACHE3-HO_1715_26
Parent CACHE3HI_1715_92
distance 0.2252

PARENT MOLECULE

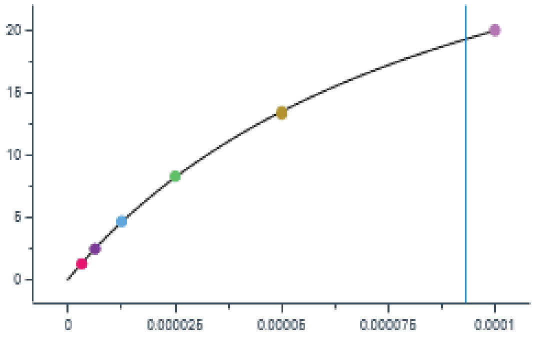

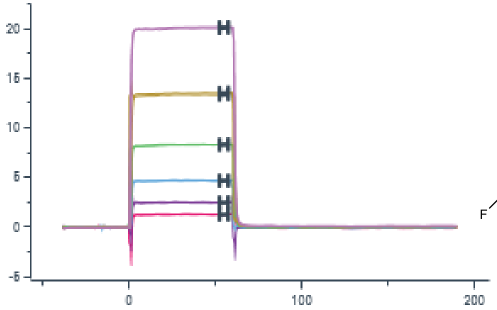

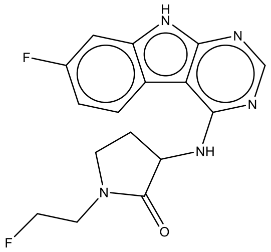

CACHE3HI_1715_95

K_D = 93 µM – 129% binding
Selectivity for NSP3 (against PARP14a protein)
DLS (solub@100 µM)
HTRF_displacement (2% DMSO):
%inh@100 uM = 35
%inh@50 uM = 21
%inh@25 uM = 11

Structure – Yes !

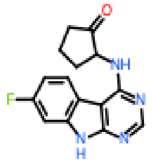

Closest published hit
dist: 0.15
Ki:12 µM

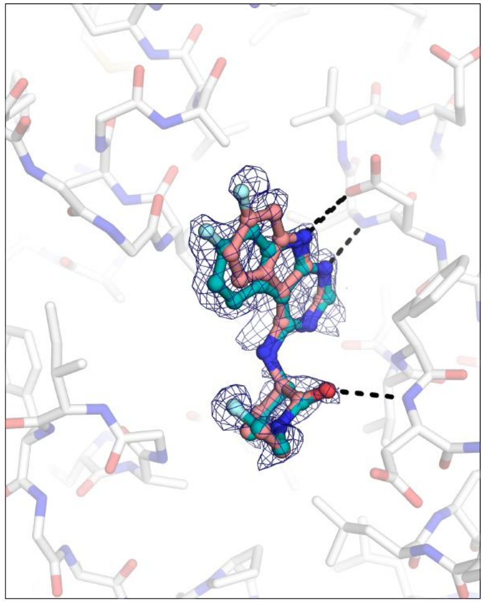

Blue mesh: PanDDA event map contoured at 2 σ

1 analog of CACHE3HI_1715_95 chemotype were submitted for round 2.
CACHE3-HO_1715_31 showed a dose depended displacement of ADP-ribose peptide by HTRF and a weak binding affinity by SPR.

CACHE3-HO_1715_31

K_D = 84 (poor fit) – 49% binding
DLS (solub@100 µM)
HTRF_displacement hit confirmation (4% DMSO):
%inh@100 uM = 86
%inh@30 uM = 43
%inh@10 uM = 2

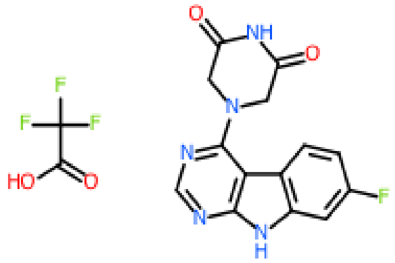

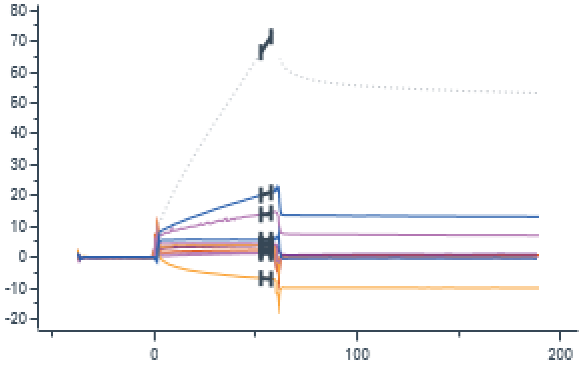

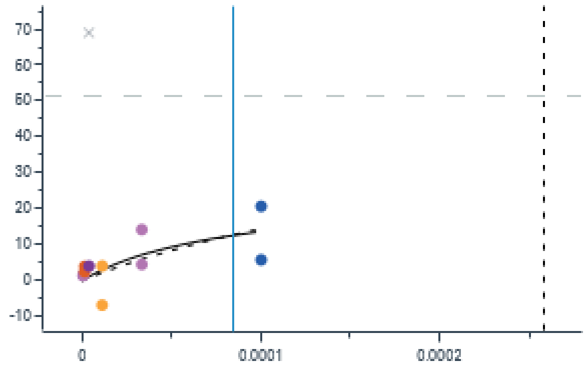

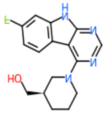

Closest published hit
dist: 0.2
Ki:79 µM

CACHE#3 – SARS-CoV2 Nsp3 macrodomain

Participant 1716

Crystallized molecule that was not advanced to round 2

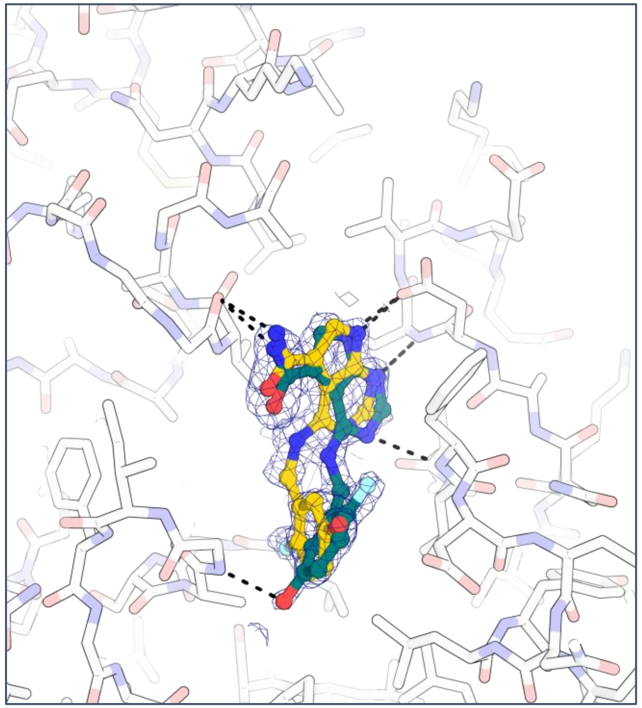

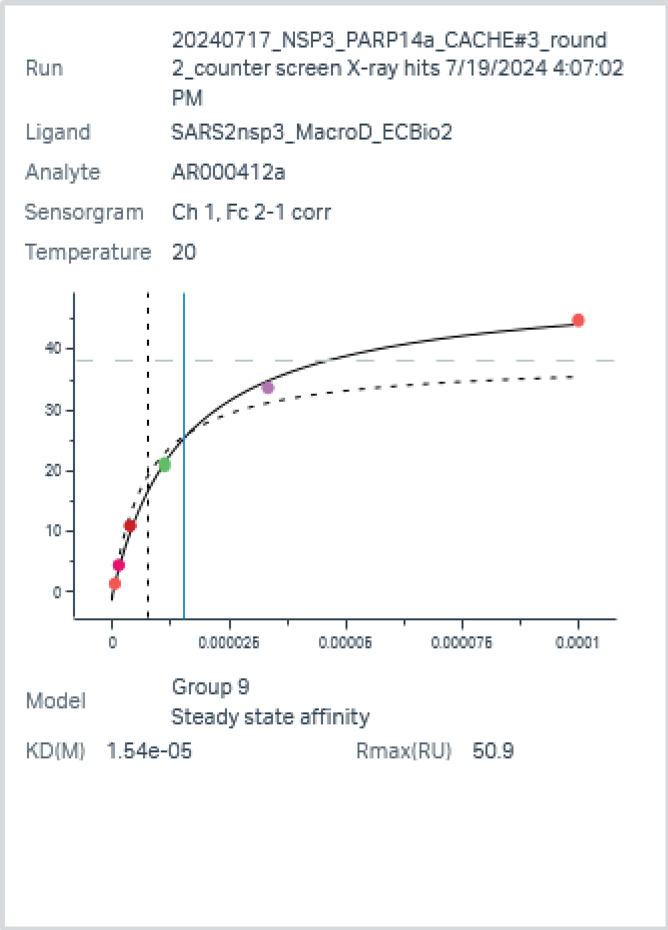

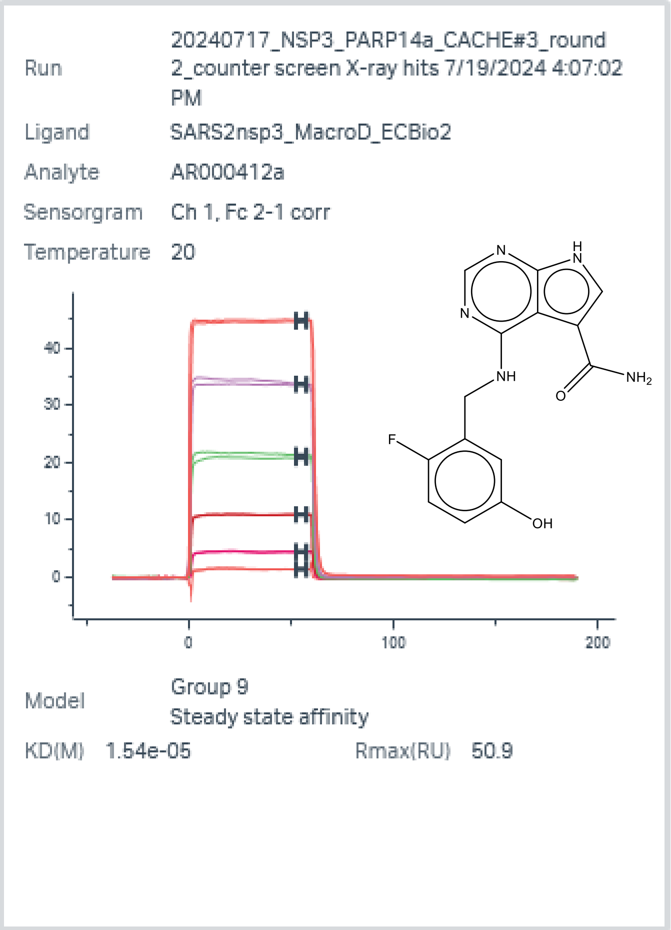

CACHE3HI_1716_34

K_D = 15 µM – 133% binding

Selectivity for NSP3 (against PARP14a protein) – mild binding

HTRF_displacement hit confirmation (2% DMSO):

%inh@100 uM = NA

%inh@50 uM = NA

%inh@25 uM = 72

-showed fluorescence interference

Structure – Yes !

Closest published hit
dist: 0.4
Ki:114 µM

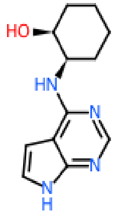

CACHE#3 – SARS-CoV2 Nsp3 macrodomain
Participant 1718

PARENT MOLECULE

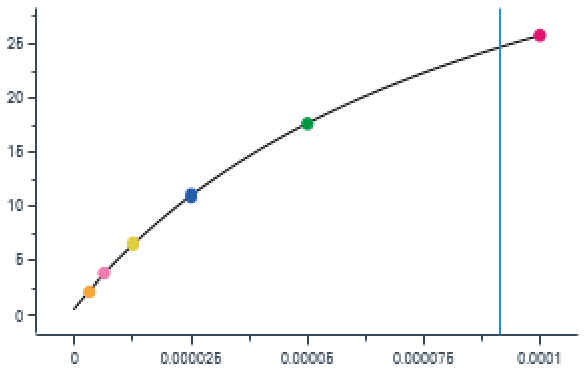

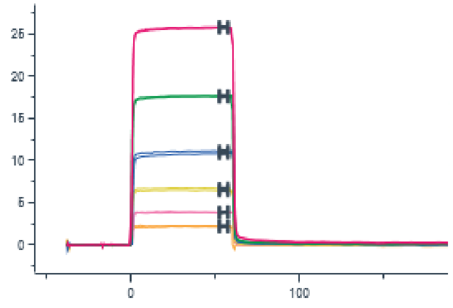

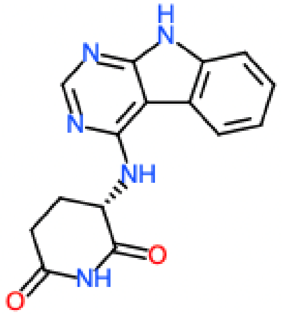

CACHE3HI_1718_58

K_D = 92 µM – 139% binding

Selectivity for NSP3 (against PARP14a protein) – Yes

DLS (solub@100 µM)

HTRF_displacement (2% DMSO):

%inh@100 uM = 36

%inh@50 uM = 17

%inh@25 uM = 10

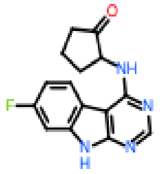

Closest published hit
dist: 0.1
Ki:12 µM

6 analogs, including parent molecule, of CACHE3HI_1718_58 chemotype were submitted for round 2.

2 compounds showed a dose depended displacement of ADP-ribose peptide by HTRF and binding affinity by SPR.

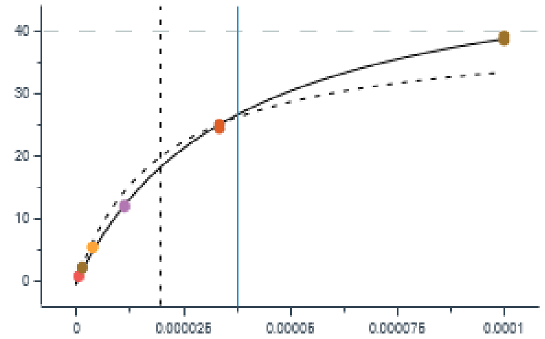

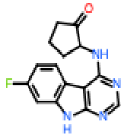

Closest published hit
dist: 0.1
Ki:12 µM

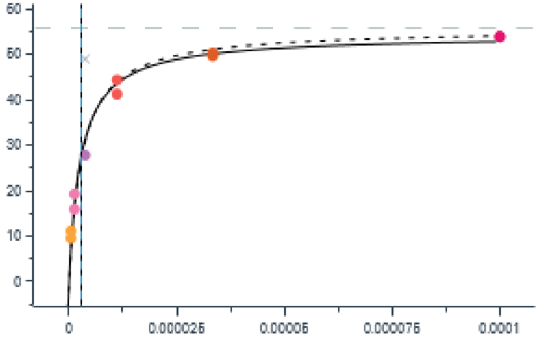

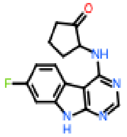

Closest published hit
dist: 0.1
Ki:12 µM

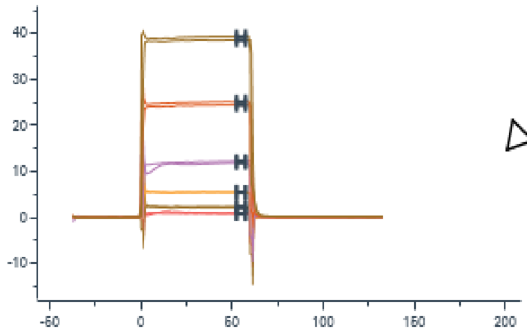

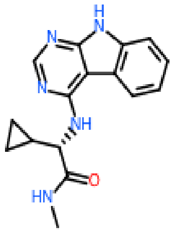

CACHE3-HO_1718_6

K_D = 38 µM – 133% binding

Selectivity for NSP3 (against PARP14a protein) – mild binding

DLS (solub@50 µM)

HTRF_displacement hit confirmation (4% DMSO):

%inh@100 uM = 61

%inh@30 uM = 27

%inh@10 uM = 21

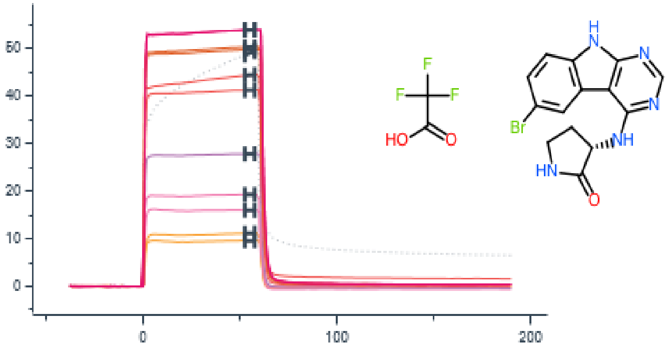

CACHE3-HO_1718_5

K_D (run 1) = 3 µM – 97% binding

K_D (run 1) = 3 µM – 81% binding

Selectivity for NSP3 (against PARP14a protein) – 14% binding

DLS (solub@100 µM)

HTRF_displacement hit confirmation (4% DMSO):

%inh@100 uM = 82

%inh@30 uM = 60

%inh@10 uM = 45

Tested analogs

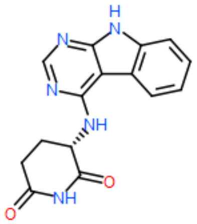

CACHE_ID CACHE3-HO_1718_21
Parent CACHE3HI_1718_58
distance 0

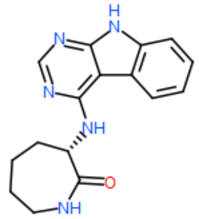

CACHE_ID CACHE3-HO_1718_3
Parent CACHE3HI_1718_58
distance 0.04615

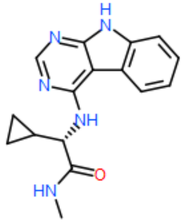

confirmed

CACHE_ID CACHE3-HO_1718_6
Parent CACHE3HI_1718_58
distance 0.0916

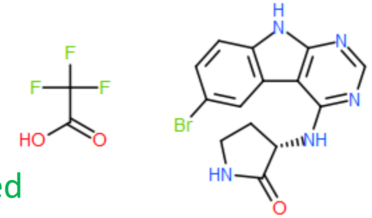

confirmed

CACHE_ID CACHE3-HO_1718_5
Parent CACHE3HI_1718_58
distance 0.0989

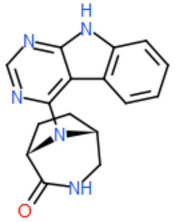

CACHE_ID CACHE3-HO_1718_2
Parent CACHE3HI_1718_58
distance 0.3724

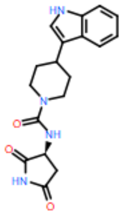

CACHE_ID CACHE3-HO_1718_19
Parent CACHE3HI_1718_58
distance 0.5559

PARENT MOLECULE

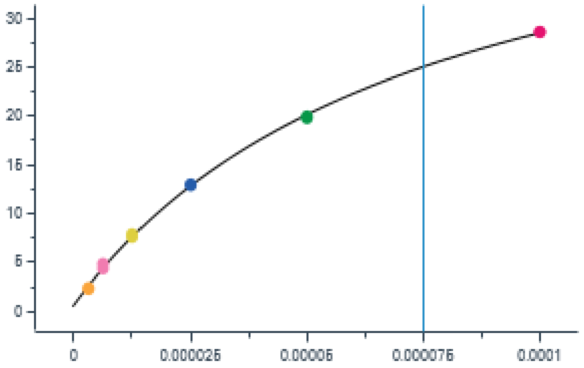

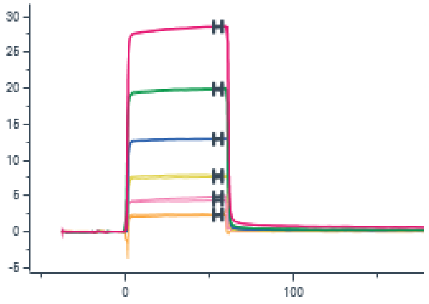

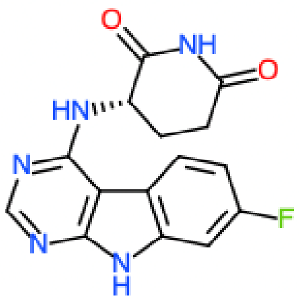

CACHE3HI_1718_59

K_D = 75 µM – 125% binding

Selectivity for NSP3 (against PARP14a protein) – 41% binding

DLS (solub@100 µM)

HTRF_displacement (2% DMSO):

%inh@100 uM = 44

%inh@50 uM = 19

%inh@25 uM = 9

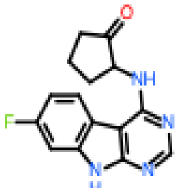

Closest published hit
dist: 0.1
Ki:12 µM

13 analogs, including parent molecule, of CACHE3HI_1718_59 chemotype were submitted for round 2.
5 compounds, including a re-supplied parent molecule, showed a dose depended displacement of ADP-ribose peptide by HTRF and binding affinity by SPR.

CACHE3-HO_1718_22

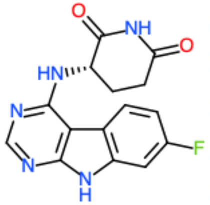

K_D = 69 µM – 128% binding

DLS (solub@100 µM)

HTRF_displacement hit confirmation (4% DMSO):

%inh@100 uM = 34

%inh@30 uM = 12

%inh@10 uM = 7

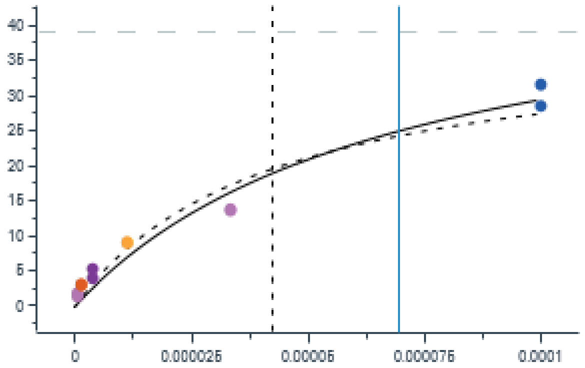

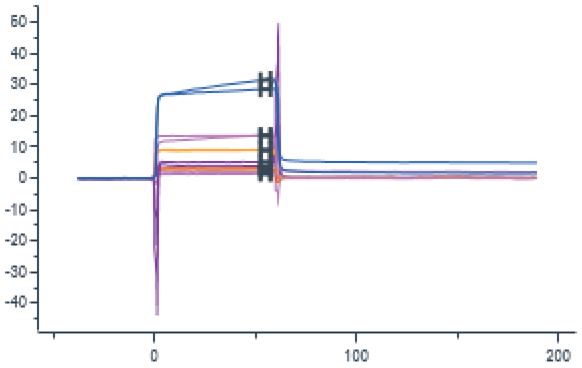

Tested analogs

confirmed

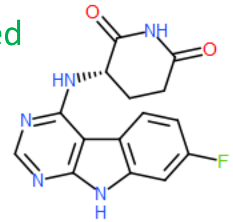

CACHE_ID CACHE3-HO_1718_22
Parent CACHE3HI_1718_59
distance 0

confirmed

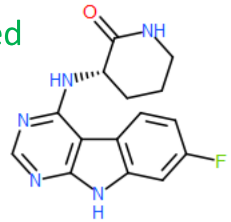

CACHE_ID CACHE3-HO_1718_17
Parent CACHE3HI_1718_59
distance 0.02247

confirmed

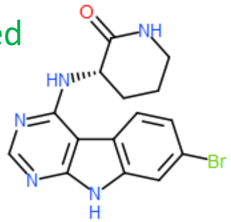

CACHE_ID CACHE3-HO_1718_16
Parent CACHE3HI_1718_59
distance 0.05147

confirmed

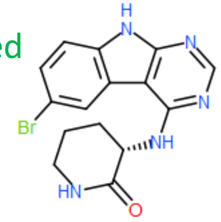

CACHE_ID CACHE3-HO_1718_4
Parent CACHE3HI_1718_59
distance 0.06909

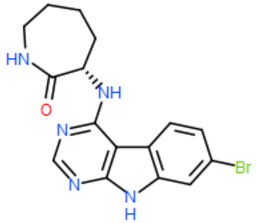

CACHE_ID CACHE3-HO_1718_14
Parent CACHE3HI_1718_59
distance 0.06909

confirmed

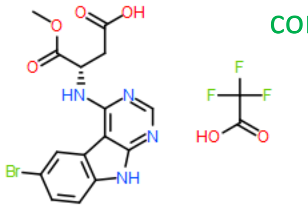

CACHE_ID CACHE3-HO_1718_7
Parent CACHE3HI_1718_59
distance 0.2516

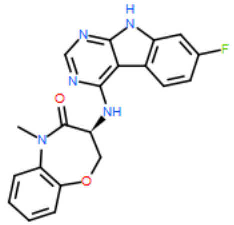

CACHE_ID CACHE3-HO_1718_8
Parent CACHE3HI_1718_59
distance 0.3173

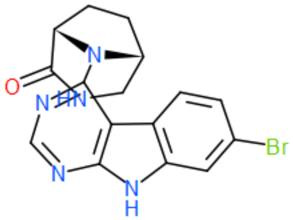

CACHE_ID CACHE3-HO_1718_1
Parent CACHE3HI_1718_59
distance 0.3689

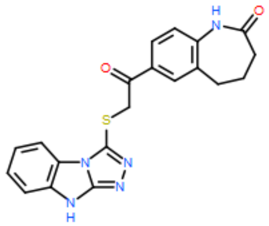

CACHE_ID CACHE3-HO_1718_10
Parent CACHE3HI_1718_59
distance 0.5782

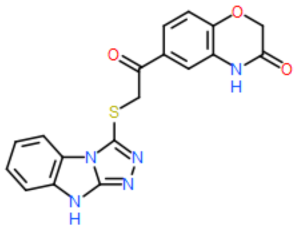

CACHE_ID CACHE3-HO_1718_9
Parent CACHE3HI_1718_59
distance 0.6109

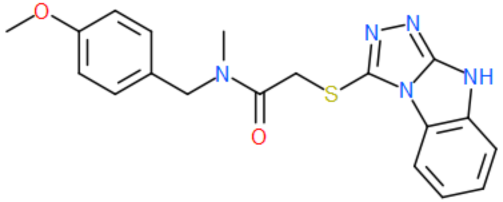

CACHE_ID CACHE3-HO_1718_11
Parent CACHE3HI_1718_59
distance 0.6388

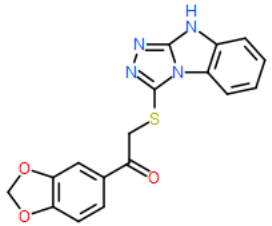

CACHE_ID CACHE3-HO_1718_12
Parent CACHE3HI_1718_59
distance 0.6403

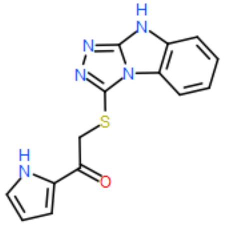

CACHE_ID CACHE3-HO_1718_18
Parent CACHE3HI_1718_59
distance 0.6494

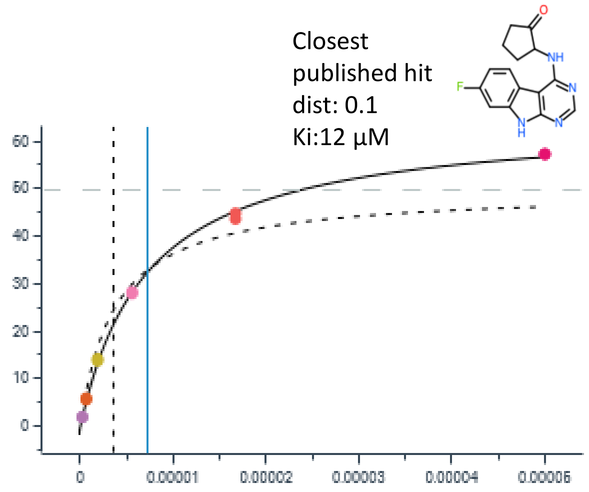

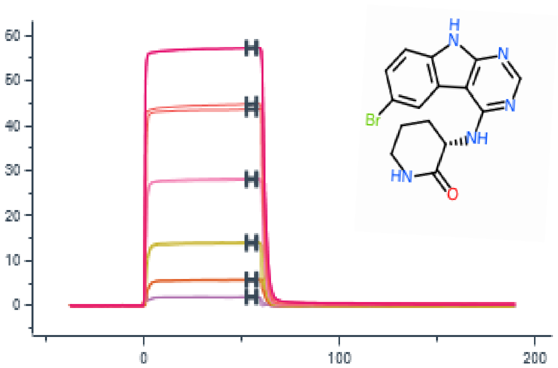

CACHE3-HO_1718_4

K_D = 7 μM – 131% binding
Selectivity for NSP3 (against PARP14a protein) – 44% binding
DLS (solub@50 μM)
HTRF_displacement hit confirmation (4% DMSO):
%inh@100 uM = 82
%inh@30 uM = 52
%inh@10 uM = 37

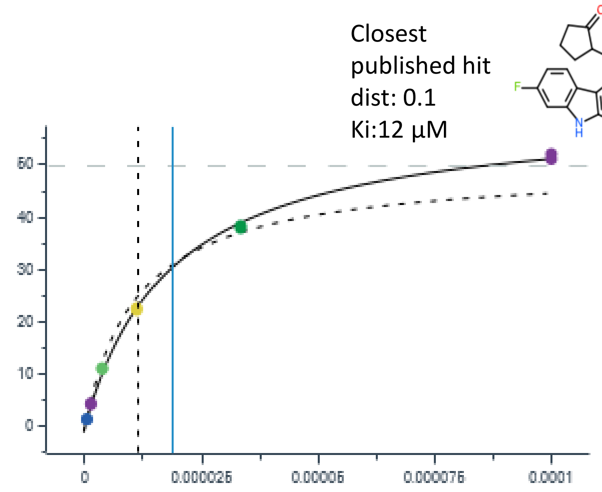

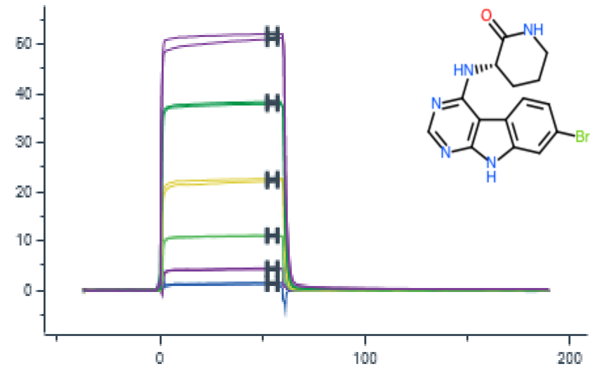

CACHE3-HO_1718_16

K_D (run 1) = 23 μM – 154% binding
K_D (run 1) = 19 μM – 122% binding
Selectivity for NSP3 (against PARP14a protein) – weak binding
DLS (solub@100 μM)
HTRF_displacement hit confirmation (4% DMSO):
%inh@100 uM = 63
%inh@30 uM = 34
%inh@10 uM = 18

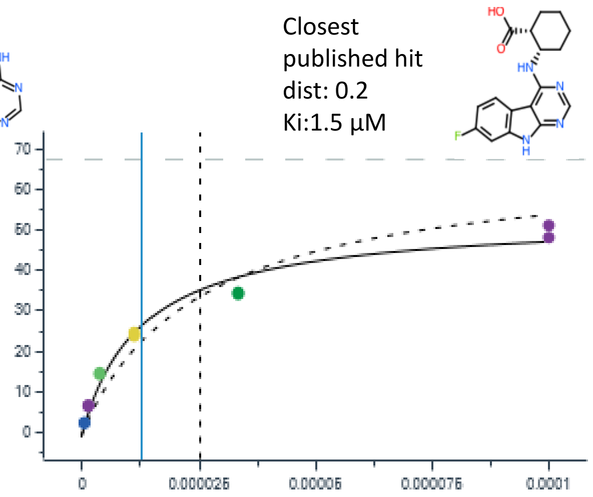

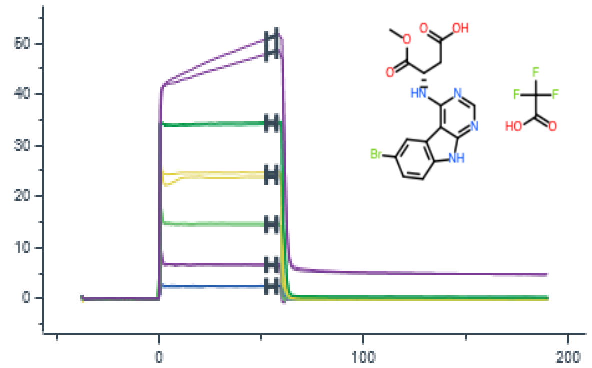

CACHE3-HO_1718_7

K_D = 13 μM – 79% binding
Selectivity for NSP3 (against PARP14a protein) – Yes
DLS (solub@100 μM)
HTRF_displacement hit confirmation (4% DMSO):
%inh@100 uM = 74
%inh@30 uM = 43
%inh@10 uM = 31

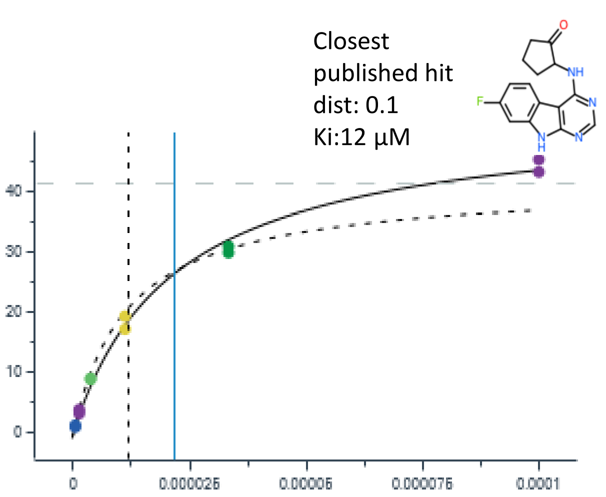

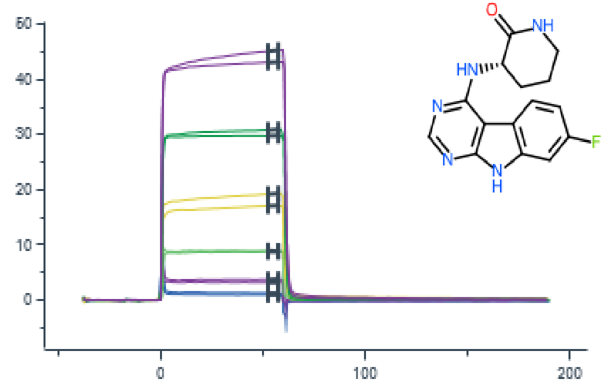

CACHE3-HO_1718_17

K_D (run 1) = 20 μM – 131% binding
K_D (run 2) = 22 μM – 129% binding
Selectivity for NSP3 (against PARP14a protein) – Yes
DLS (solub@100 μM)
HTRF_displacement hit confirmation (4% DMSO):
%inh@100 uM = 66
%inh@30 uM = 28
%inh@10 uM = 21
